# Supplementary material for: Values and Diagnostic Accuracy of Electrodiagnostic Findings in Carpal Tunnel Syndrome Based on Age, Gender, and Diabetes
Source: Diagnostics (Basel). 2024 Jun 28;14(13):1381. doi: 10.3390/diagnostics14131381 (PMC11240809; doi:10.3390/diagnostics14131381)
Supplement: Supplementary file 1 [file diagnostics-14-01381-s001.zip › Table S1 Values of median nerve and comparative latency studies (COLSs) based on gender.pdf]

Table S1 values of median nerve and comparative latency studies (COLS) based on gender

	Whole cohort			female			Male		
	No CTS ms median (IQR)/95% ULN	CTS ms median (IQR)	p value	No CTS ms median (IQR)/95% ULN	CTS ms median (IQR)	p value	No CTS ms median (IQR)/95% ULN	CTS ms median (IQR)	p value
Medial sensory latency at digit-II									
All age groups	3.2 (3-3.4)/2.8	3.8 (3.3-4.8)	0.000	3.1 (2.9-3.3)/4	3.8 (3.3-4.6)	0.000	3.2 (3-3.5)/4.3	4.0 (3.5-5.2)	0.000
Group1 < 30 years	3 (2.8-3.2)/4.3	3 (2.8-3.5)	0.52	3 (2.9-3.1)/3.4	3 (2.8-3.5)	0.61	2.9 (2.7-3.5)/4	2.9 (2.8-3.5)	0.69
Group2 30-39 years	3.1 (2.9-3.2)/3.6	3.5 (3.1-4.4)	0.0001	3.1 (2.9-3.2)/4.2	3.3 (3.1-4.2)	0.021	3.1 (2.9-3.2)/3.5	4.1 (3.7-4.6)	0.0017
Group3 40-49 years	3 (2.9-3.3)/3.9	3.6 (3.3-4.5)	0.000	3 (2.9-3.2)/3.3	3.6 (3.3-4.5)	0.000	3.2 (2.9-3.5)/4	3.6 (3.5-4)	0.037
Group4 50-59 years	3.3 (3.1-3.6)/ 4.3	4.1 (3.5-5.3)	0.000	3.2 (3.1-3.6)/4	4 (3.5-5.2)	0.000	3.3 (3.1-3.5)/4.3	4.4 (3.4-5.5)	0.001
Group5 >60 years	3.4 (3.2-3.8)/ 4.6	4.4 (3.7-5.2)	0.000	3.4 (3.3-4)/4.6	4.3 (3.7-5.2)	0.000	3. 5(3.2-3.8)/4.8	4.5 (3.8-5.2)	0.000
Median Motor latency at abductor pollicis brevis (APB)									
All age groups	3.3(3.0-3.6)/2.8	4.2 (3.5-5.7)	0.000	3.3 (3-3.5)/2.8	4.2 (3.5-5.7)	0.000	3.3 (3-3.8)/4.9	4.6 (3.7-5.9)	0.000
Group1 < 30 years	3.1 (3-3.3)3.5	3.1 (2.8-3.9)	0.64	3.2 (3-3.3)/3.4	3.1 (2.8-3.9)	0.87	3 (2.8-3.4)/3.7	3.2 (2.9-3.6)	0.60
Group2 30-39 years	3.2 (3-3.4)/4	3.7 (3.2-4.5)	0.000	3.2 (3-3.4)/2.4	3.7 (3.2-4.4)	0.16	3.2 (3-3.4)/3.8	4.0 (3.7-4.6)	0.000
Group3 40-49 years	3.3 (3.1-3.6)/4	4 (3.4-5.4)	0.000	3.2(3.1-3.5)/3.7	4.0 (3.4-5.5)	0.000	3.3 (3.1-3.7)/4.3	4 (3.8-5.3)	0.000
Group4 50-59 years	3.3 (3.1-3.8)/ 5.3	4.4 (3.6-5.9)	0.000	3.3(3-3.6)/4.5	4.4 (3.7-6)	0.000	3.5 (3.1-3.9)/5.4	4.4 (3.5-5.5)	0.007
Group5 >60 years	3.8 (3.3-4.1)/4.9	5.2 (4.3-6.5)	0.000	3.8(3.4-4.1)/4.8	5.2 (4.3-6.3)	0.000	3.8 (3.3-4.1)/5.1	5.6 (4.6-7.5)	0.000
Palmdiff: Median (palm)-Ulnar(palm)									
All age groups	0.1 (0-0.3)/0	0.7 (0.3-1.3)	0.000	0.1 (0-0.3)/0.7	0.7 (0.3-1.2)	0.000	0.1 (0-0.3)/0.9	0.7 (0.4-1.5)	0.000

Group1 < 30 years	0.1 (0-0.2)/0.3	0.25 (0-0.4)	0.036	0.1 (0-0.2)/0.3	0.15 (0-0.3)	0.15	0 (0-0.3)0.4	0.4 (0.1-0.6)	0.09
Group2 30-39 years	0.1 (0-0.3)/0.4	0.45 (0.2-1)	0.000	0.1 (0-0.3)/0.7	0.4 (0.1-0.9)	0.007	0.1 (0-0.2)/0.4	0.5 (0.4-1.4)	0.000
Group3 40-49 years	0.1 (0-0.2)/0.6	0.8 (0.3-1.3)	0.000	0.1 (0-0.2)/0.3	0.8 (0.3-1.3)	0.000	0.1 (0.1-0.3)/0.9	0.7 (0.3-2.1)	0.000
Group4 50-59 years	0.2 (0-0.4)/0.8	0.7 (0.4-1.4)	0.000	0.2(0-0.4)/0.8	0.7 (0.4-1.3)	0.000	0.1 (0-0.4)/1.1	0.5 (0.4-1.4)	0.000
Group5 >60 years	0.3 (0.1-0.4)/1.2	0.9 (0.6-1.5)	0.000	0.3(0.2-0.5)/1.3	0.8 (0.6-1.4)	0.001	0.3 (0-0.4)/1.1	1.0 (0.8-1.7)	0.000
Thumbdiff: Median (D1)-Radial(D1)									
All age groups	0.4 (0.2-0.6)/0	1.0 (0.6-1.7	0.000	0.3 (0.2-0.5)/1.1	0.9 (0.6-1.6)	0.000	0.4 (0.2-0.7)/1.3	1.1 (0.7-2)	0.000
Group1 < 30 years	0.3 (0.2-0.4)/0.6	0.6 (0.4-1.1)	0.0001	0.3 (0.2-0.4)/0.6	0.6 (0.4-0.9)	0.0013	0.2 (0.2-0.3)/0.5	0.8 (0.4-1.1)	0.019
Group2 30-39 years	0.35 (0.1-0.6)/1	0.8 (0.4-1.2)	0.0002	0.4 (0.2-0.6)/1.3	0.7 (0.4-1.2)	0.49	0.3 (0.1-0.5)/0.8	0.9 (0.6-1.9)	0.001
Group3 40-49 years	0.3 (0.2-0.6)/1.1	1 (0.6-1.6)	0.000	0.2 (0.1-0.3)/0.6	0.9 (0.5-1.7)	0.000	0.6 (0.2-0.7)/1.3	1 (0.9-1.3	0.003
Group4 50-59 years	0.4 (0.3-0.6)/1.1	1 (0.7-1.8)	0.000	0.3 (0.1-0.5)/1.1	1 (0.7-1.8)	0.000	0.5 (0.4-0.7)/0.9	1.2 (0.6-2.1)	0.000
Group5 >60 years	0.5 (0.3-0.9)/1.4	1.4 (0.8-2.3)	0.000	0.6 (0.5-1)/1.4	1.1 (0.8-2.1)	0.002	0.4 (0.2-0.9)/1.7	1.7 (1.1-2.5)	0.000
Ringdiff: Median (Digit IV)-Ulnar(Digit IV)									
All age groups	0.1 (0-0.3)/1	0.7 (0.3-1.5)	0.000	0.1 (0-0.3)/1	0.7 (0.2-1.5)	0.000	0.1 (0-0.3)/0.9	0.6 (0.5-1.7)	0.000
Group1 < 30 years	3 (2.8-3.2)/4.3	3 (2.8-3.5)/	0.52	3 (2.9-3.1)/3.4	3 (2.8-3.5)	0.61	2.9 (2.7-3.5)/4	2.9 (2.8-3.5)	0.69
Group2 30-39 years	0.1 (0-0.2)/0.5	0.4 (0.1-1.2)	0.003	0.2 (0-0.3)/0.9	0.35 (0.1-1.2)	0.05	0.1 (0-0.2)/0.4	0.5 (0.9-1.8)	0.004
Group3 40-49 years	0 (0-0.2)/0.7	0.5 (0.3-1.4)	0.000	0 (0-0.2)/0.4	0.5 (0.3-1.5)	0.000	0 (0-0.3)/0.7	0.6 (0.2-0.9	0.006
Group4 50-59 years	0.1 (0-0.5)/1.4	0.8 (0.35-1.8)	0.000	0.2 (0-0.5)/1	0.8 (0.3-1.7)	0.000	0.1 (0-0.5)/1.4	0.8 (0.4-2.3)	0.001
Group5 >60 years	0.2 (0-0.6)/1.4	1 (0.6-1.4)	0.000	0.4 (0.1-1)/1.1	1.1 (0.6-1.5)	0.001	0.2 (0-0.5)/1.4	0.8 (0.5-1.3	0.011

CSI	Combined sensory index								
All age groups	0.6 (0.4-1.1)/2.6	2.4 (1.3-4.25)	0.000	0.6 (0.3-1)/2.7	2.4 (1.3-4.1)/7	0.000	0.65 (0.4-1.2)/2.6	2.9 (1.5-4.8)	0.000
Group1 < 30 years	0.5 (0.3-0.7)/1.1	1.1 (0.6-1.6)	0.000	0.55 (0.3-0.7)/0.8	1 (0.6-1.6)/4	0.002	0.5 (0.2-0.7)/1.1	1.55 (1-2.4)	0.025
Group2 30-39 years	0.5 (0.3-0.9)/1.4	1.6 (0.5-3.3)	0.000	0.7 (0.3-1)/2.9	1.5 (0.5-3.2)	0.031	0.45 (0.2-0.9)/1.2	1.8 (1.3-4.7)	0.000
Group3 40-49 years	0.6 (0.2-0.8)/2.3	2.3 (1.3-4.4)	0.000	0.4 (0.2-0.7)/1	2.3 (1.3-4.6)/6	0.000	0.75 (0.5-1.2)/2.6	2.3 (2.2-4.3)	0.002
Group4 50-59 years	0.7 (0.4-1.4)/2.6	2.6 (1.6-4.6)	0.000	0.75 (0.4-1.25)	2.5 (1.6-4.1)	0.000	0.75 (0.4-1.7)/2.6	2.8 (1.3-5.5)	0.000
Group5 >60 years	1.1 (0.6-1.9)/4.2	3.2 (2.3-4.7)	0.000	1.3 (0.7-2.6)/3.8	3.05 (2.1-4.8)	0.001	0.8 (0.5-1.9)/4.2	3.7 (3-4.4)	0.000

IQR: Interquartile range. ULN: upper limit of normal
