# Supplementary material for: Values and Diagnostic Accuracy of Electrodiagnostic Findings in Carpal Tunnel Syndrome Based on Age, Gender, and Diabetes
Source: Diagnostics (Basel). 2024 Jun 28;14(13):1381. doi: 10.3390/diagnostics14131381 (PMC11240809; doi:10.3390/diagnostics14131381)
Supplement: Supplementary file 1 [file diagnostics-14-01381-s001.zip › Table S2 Cut-off values and diagnostic accuracy of median nerve and comparative latency studies (COLSs), females.pdf]

Table S2 Cut-off values and diagnostic accuracy of median nerve and comparative latency studies (COLS), Female.

Age group	Cuf off	ROC	Sensitivity	Specificity	PPV	NPV
	Median sensory latency at Digit II					
Whole cohort	3.5 ms	0.755 (0.711-0.8)	67.6% (61.8%-73%)	83.5% (75.2%-89.9%)	91.4% (86.8%-94.8%)	49.7% (42.3%-57.2%)
Group1 < 30 years	3.5 ms	0.658 (0.551-0.765)	31.6% (12.6%-56.6%)	100% (85.2%-100%)	100% (54.1%-100%)	63.9% (46.2%-79.2%)
Group2 30-39 years	3.6 ms	0.657 (0.549-0.765)	43.2% (28.3%-59%)	88.2% (63.6%-98.5%)	90.5% (69.6%-98.8%)	37.5% (22.7%-54.2%)
Group3 40-49 years	3.5 ms	0.799 (0.733-0.865)	63.5% (51.5%-74.4%)	96.3% (81%-99.9%)	97.9% (88.9%-99.9%)	49.1% (35.1%-63.2%)
Group4 50-59 years	3.5 ms	0.74 (0.643-0.836)	77.6% (68.5%-85.1%)	70.4% (49.8%-86.2%)	91.2% (83.4%-96.1%)	44.2% (29.1%-60.1%)
Group4 >60 years	4.3 ms	0.754 (0.653-0.856)	57.5% (40.9%-73%)	93.3% (68.1%-99.8%)	95.8% (78.9%-99.9%)	45.2% (27.3%-64%)
Palmdiff	Mixed palmar studies (palmdiff): Median latency (palm)-Ulnar latency(palm)					
Whole cohort	0.4	0.784 (0.739- 0.829)	71.8% (65.8%- 77.3%)	85% (76.5%- 91.4%)	92.3% (87.7%- 95.7%)	54.5% (46.3%- 62.5%)
Group1 < 30 years	0.4	0.611 (0.512- 0.71)	22.2% (6.41%- 47.6%)	100% (85.2%- 100%)	100% (39.8%- 100%)	62.2% (44.8%- 77.5%)
Group2 30-39 years	0.4	0.731 (0.632- 0.831)	52.5% (36.1%- 68.5%)	93.8% (69.8%- 99.8%)	95.5% (77.2%- 99.9%)	44.1% (27.2%- 62.1%)
Group3 40-49 years	0.4	0.835 (0.767- 0.904)	71.4% (59.4%- 81.6%)	95.7% (78.1%- 99.9%)	98% (89.6%- 100%)	52.4% (36.4%- 68%)
Group4 50-59 years	0.4	0.761 (0.658- 0.864)	85.6% (76.6%- 92.1%)	66.7% (44.7%- 84.4%)	90.6% (82.3%- 95.8%)	55.2% (35.7%- 73.6%)
Group4 >60 years	0.8	0.693 (0.566- 0.821)	52.9% (35.1%- 70.2%)	85.7% (57.2%- 98.2%)	90% (68.3%- 98.8%)	42.9% (24.5%- 62.8%)
Thumdiff	Median to radial comparison, thumbdiff: Median latency (D1)-Radial latency (D1)					
Whole cohort	0.7	0.758	68%	83.7%	91%	51.8%

		(0.712- 0.804)	(61.9%- 73.7%)	(75.1%- 90.2%)	(86%- 94.7%)	(44%- 59.5%)
Group1 < 30 years	0.8	0.667 (0.555- 0.779)	33.3% (13.3%- 59%)	100% (84.6%- 100%)	100% (54.1%- 100%)	64.7% (46.5%- 80.3%)
Group2 30-39 years	0.8	0.676 (0.562- 0.789)	47.6% (32%- 63.6%)	87.5% (61.7%- 98.4%)	90.9% (70.8%- 98.9%)	38.9% (23.1%- 56.5%)
Group3 40-49 years	0.5	0.844 (0.766- 0.922)	80.3% (69.1%- 88.8%)	88.5% (69.8%- 97.6%)	95% (86.1%- 99%)	62.2% (44.8%- 77.5%)
Group4 50-59 years	0.5	0.8 (0.702- 0.897)	92% (84.1%- 96.7%)	68% (46.5%- 85.1%)	90.9% (82.9%- 96%)	70.8% (48.9%- 87.4%)
Group4 >60 years	1.4	0.695 (0.589- 0.801)	45.7% (28.8%- 63.4%)	93.3% (68.1%- 99.8%)	94.1% (71.3%- 99.9%)	42.4% (25.5%- 60.8%)
Ringdiff	Median to ulnar comparison at ring finger study (ringdiff): Median latency (Digit IV)-Ulnar latency(Digit IV)					
Whole cohort	0.6	0.711 (0.666- 0.756)	54.5% (47.9%- 61%)	87.7% (79.9%- 93.3%)	90.7% (84.6%- 95%)	46.7% (39.6%- 53.9%)
Group1 < 30 years	0.7	0.611 (0.512- 0.71)	22.2% (6.41%- 47.6%)	100% (85.2%- 100%)	100% (39.8%- 100%)	62.2% (44.8%- 77.5%)
Group2 30-39 years	0.6	0.694 (0.596- 0.793)	44.7% (28.6%-61.7%)	94.1% (71.3%- 99.9%)	94.4% (72.7%- 99.9%)	43.2% (27.1%- 60.5%)
Group3 40-49 years	0.4	0.768 (0.681- 0.856)	65.6% (52.7%- 77.1%)	88% (68.8%- 97.5%)	93.3% (81.7%- 98.6%)	50% (34.6%- 65.4%)
Group4 50-59 years	0.4	0.682 (0.593- 0.771)	51.8% (40.6%- 62.9%)	84.6% (65.1%- 95.6%)	91.5% (79.6%- 97.6%)	35.5% (23.7%- 48.7%)
Group4 >60 years	1.2	0.733 (0.643- 0.824)	46.7% (28.3%-65.7%)	100% (78.2%-100%)	100% (76.8%-100%)	48.4% (30.2%-66.9%)
CSI	Combined sensory index: sum of palmdiff + thumbdiff + ringdiff					
Whole cohort	1.1	0.791 (0.743-00.839)	79.6% (74.1%- 84.4%)	78.6% (69.1% -86.2%)	90.5% (85.8%-94%)	60.2% (51.1%-68.7%)
Group1 < 30 years	1.0	0.755 (0.629- 0.881)	55.6% (30.8%- 78.5%)	95.5% (77.2%- 99.9%)	90.9% (58.7%- 99.8%)	72.4% (52.8%- 87.3%)
Group2 30-39 years	1.3	0.707(0.59- 0.824)	54.8% (38.7%- 70.2%)	86.7% (59.5%- 98.3%)	92% (74% -99%)	40.6% (23.7%- 59.4%)
Group3 40-49 years	0.9	0.876	83.8%	91.3%	96.6%	65.6%

		(0.802- 0.949)	(72.9%- 91.6%)	(72%- 98.9%)	(88.3%- 99.6%)	(46.8%- 81.4%)
Group4 50-59 years	1.1	0.793<br>(0.694-0.892)	87.8%<br>(79.2%-93.7%)	70.8%<br>(48.9%-87.4%)	91.9%<br>(83.9%-96.7%)	60.7%<br>(40.6%-78.5%)
Group4 >60 years	3.5	0.652<br>(0.542- 0.762)	37.5%<br>(21.1%- 56.3%)	92.9%<br>(66.1%- 99.8%)	92.3%<br>(64%- 99.8%)	39.4%<br>(22.9%- 57.9%)
