# Supplementary material for: Values and Diagnostic Accuracy of Electrodiagnostic Findings in Carpal Tunnel Syndrome Based on Age, Gender, and Diabetes
Source: Diagnostics (Basel). 2024 Jun 28;14(13):1381. doi: 10.3390/diagnostics14131381 (PMC11240809; doi:10.3390/diagnostics14131381)
Supplement: Supplementary file 1 [file diagnostics-14-01381-s001.zip › Table S4 Diagnostic accuracy of median sensory latency at Digit II and comparative latency studies (COLSs), all cutoff values (whole cohort).pdf]

Table S4 Diagnostic accuracy of median sensory latency at Digit II, All cutoff values

Age group	ROC	Sensitivity	Specificity	PPV	NPV
Cut off	3.5 (ms)				
Whole cohort	.729 (.692-.766)	69.6% (64.5%-74.4%)	76.2% (70.1%-81.6%)	81.9% 77.1% 86.1%	61.8% (55.8%-67.5%)
Group1 < 30 years	.608 (.5-.716)	30.4% (13.2%-52.9%)	91.2% (76.3%-98.1%)	81.9% (77.1%-86.1%)	61.8% (55.8%-67.5%)
Group2 30-39 years	.697 (.612-.781)	51% (36.6%-65.2%)	88.4% (74.9%-96.1%)	83.9% (66.3%-94.5%)	60.3% (47.2%-72.4%)
Group3 40-49 years	.746 (.675-.817)	65.5% (54.3%-75.5%)	83.6% (71.2%-92.2%)	85.9% (75%-93.4%)	61.3% (49.4%-72.4%)
Group4 50-59 years	.724 (.65-.797)	76.1% (68.1%-82.9%)	68.6% (54.1%-80.9%)	86.8% (79.4%-92.2%)	51.5% (39%-63.8%)
Group4 >60 years	.726 (.644-.808)	92.9% (82.7%-98%)	52.3% (36.7%-67.5%)	71.2% (59.4%-81.2%)	85.2% (66.3%-95.8%)
Cut off	3.6 (ms)				
Whole cohort	.706 (.669-.742)	60.5% (55.2%-65.7%)	80.6% (74.9%-85.5%)	82.9% (77.7%-87.3%)	56.8% (51.2%-62.3%)
Group1 < 30 years	.579 (.484-.674)	21.7% (7.46%-43.7%)	94.1% (80.3%-99.3%)	71.4% (29%-96.3%)	64% (49.2%-77.1%)
Group2 30-39 years	.71 (.631-.789)	49% (34.8%-63.4%)	93% (80.9%-98.5%)	89.3% (71.8%-97.7%)	60.6% (47.8%-72.4%)
Group3 40-49 years	.707 (.636-.778)	56% (44.7%-66.8%)	85.5% (73.3%-93.5%)	85.5% (73.3%-93.5%)	56% (44.7%-66.8%)
Group4 50-59 years	.691 (.619-.764)	63.8% (55.2%-71.8%)	74.5% (60.4%-85.7%)	87.1% (79%-93%)	43.2% (32.7%-54.2%)
Group4 >60 years	.724 (.637-.811)	85.7% (73.8%-93.6%)	59.1% (43.2%-73.7%)	72.7% (60.4%-83%)	76.5% (58.8%-89.3%)
Cut off	3.7 (ms)				
Whole cohort	.714 (.68-.749)	57.4% (52%-62.6%)	85.5% (80.2%-89.8%)	86% (80.8%-90.1%)	56.4% (51%-61.7%)
Group1 < 30 years	.579 (.484-.674)	21.7% (7.46%-43.7%)	94.1% (80.3%-99.3%)	71.4% (29%-96.3%)	64% (49.2%-77.1%)
Group2 30-39 years	.702 (.626-.778)	45.1% (31.1%-59.7%)	95.3% (84.2%-99.4%)	92% (74%-99%)	59.4% (46.9%-71.1%)
Group3 40-49 years	.68 (.611-.75)	48.8% (37.7%-60%)	87.3% (75.5%-94.7%)	85.4% (72.2%-93.9%)	52.7% (42%-63.3%)
Group4 50-59 years	.737 (.672-.801)	63% (54.4%-71.1%)	84.3% (71.4%-93%)	91.6% (84.1%-96.3%)	45.7% (35.4%-56.3%)
Group4 >60 years	.752 (.666-.838)	82.1% (69.6%-91.1%)	68.2% (52.4%-81.4%)	76.7% (64%-86.6%)	75% (58.8%-87.3%)

Cut off	3.8 (ms)				
Whole cohort	.7(.666-.734)	52.8%(47.5%-58.2%)	87.2%(82.2%-91.3%)	86.5%(81.2%-90.8%)	54.4%(49.1%-59.6%)
Group1 < 30 years	.594(.503-.685)	21.7% (7.46%-43.7%)	97.1%(84.7%-99.9%)	83.3%(35.9%-99.6%)	64.7% (50.1%-77.6%)
Group2 30-39 years	.683 (.607-.758)	41.2%(27.6%-55.8%)	95.3% (84.2%-99.4%)	91.3%(72%-98.9%)	57.7%(45.4%-69.4%)
Group3 40-49 years	.681 (.615-.747)	45.2%(34.3%-56.5%)	90.9%(80%-97%)	88.4% (74.9%-96.1%)	52.1%(41.6%-62.4%)
Group4 50-59 years	.721 (.658-.784)	58% (49.3%-66.3%)	86.3%(73.7%-94.3%)	92%(84.1%-96.7%)	43.1% (33.4%-53.3%)
Group4 >60 years	.716 (.626-.806)	75%(61.6%-85.6%)	68.2%(52.4%-81.4%)	75% (61.6%-85.6%)	68.2% (52.4%-81.4%)
Cut off	3.9 (ms)				
Whole cohort	.7 (.666-.734)	52.8% (47.5%-58.2%)	87.2% (82.2%-91.3%)	86.5% (81.2%-90.8%)	54.4% (49.1%-59.6%)
Group1 < 30 years	.594 (.503-.685)	21.7% (7.46%-43.7%)	97.1% (84.7%-99.9%)	83.3% (35.9%-99.6%)	64.7% (50.1%-77.6%)
Group2 30-39 years	.683 (.607-.758)	41.2% (27.6%-55.8%)	95.3% (84.2%-99.4%)	91.3% (72%-98.9%)	57.7% (45.4%-69.4%)
Group3 40-49 years	.681 (.615-.747)	45.2% (34.3%-56.5%)	90.9% (80%-97%)	88.4% (74.9%-96.1%)	52.1% (41.6%-62.4%)
Group4 50-59 years	.721 (.658-.784)	58% (49.3%-66.3%)	86.3% (73.7%-94.3%)	92% (84.1%-96.7%)	43.1% (33.4%-53.3%)
Group4 >60 years	.716 (.626-.806)	75% (61.6%-85.6%)	68.2% (52.4%-81.4%)	75% (61.6%-85.6%)	68.2% (52.4%-81.4%)
Cut off	4.0 (ms)				
Whole cohort	.695 (.664-.727)	47.4% (42.1%-52.8%)	91.6% (87.2%-94.9%)	89.8% (84.5%-93.7%)	52.9% (47.9%-58%)
Group1 < 30 years	.594 (.503-.685)	21.7% (7.46%-43.7%)	97.1% (84.7%-99.9%)	83.3% (35.9%-99.6%)	64.7% (50.1%-77.6%)
Group2 30-39 years	.655 (.586-.724)	33.3% (20.8%-47.9%)	97.7% (87.7%-99.9%)	94.4% (72.7%-99.9%)	55.3% (43.4%-66.7%)
Group3 40-49 years	.678 (.62-.736)	39.3% (28.8%-50.5%)	96.4% (87.5%-99.6%)	94.3% (80.8%-99.3%)	51% (41%-60.9%)
Group4 50-59 years	.719 (.66-.778)	53.6% (44.9%-62.1%)	90.2% (78.6%-96.7%)	93.7% (85.8%-97.9%)	41.8% (32.5%-51.6%)
Group4 >60 years	.726 (.638-.814)	67.9% (54%-79.7%)	77.3% (62.2%-88.5%)	79.2% (65%-89.5%)	65.4% (50.9%-78%)
Cut off	4.1 (ms)				
Whole cohort	.685 (.655-.715)	43.2% (37.9%-48.5%)	93.8% (89.9%-96.6%)	91.6% (86.3%-95.3%)	51.6% (46.6%-56.5%)
Group1 < 30 years	.543 (.485-.602)	8.7% (1.07%-28%)	100% (89.7%-100%)	100% (15.8%-100%)	61.8% (47.7%-74.6%)

Group2 30-39 years	.645 (.577-.713)	31.4% (19.1%-45.9%)	97.7% (87.7%-99.9%)	94.1% (71.3%-99.9%)	54.5% (42.8%-65.9%)
Group3 40-49 years	.658 (.604-.711)	33.3% (23.4%-44.5%)	98.2% (90.3%-100%)	96.6% (82.2%-99.9%)	49.1% (39.4%-58.8%)
Group4 50-59 years	.714 (.658-.77)	50.7% (42.1%-59.3%)	92.2% (81.1%-97.8%)	94.6% (86.7%-98.5%)	40.9% (31.8%-50.4%)
Group4 >60 years	.731 (.645-.816)	64.3% (50.4%-76.6%)	81.8% (67.3%-91.8%)	81.8% (67.3%-91.8%)	64.3% (50.4%-76.6%)
Cut off	4.2 (ms)				
Whole cohort	.677 (.647-.707)	41.2% (36%-46.5%)	94.3% (90.4%-96.9%)	91.8% (86.3%-95.5%)	50.8% (45.9%-55.7%)
Group1 < 30 years	.543 (.485-.602)	8.7% (1.07%-28%)	100% (89.7%-100%)	100% (15.8%-100%)	61.8% (47.7%-74.6%)
Group2 30-39 years	.635 (.568-.703)	29.4% (17.5%-43.8%)	97.7% (87.7%-99.9%)	93.8% (69.8%-99.8%)	53.8% (42.2%-65.2%)
Group3 40-49 years	.658 (.604-.711)	33.3% (23.4%-44.5%)	98.2% (90.3%-100%)	96.6% (82.2%-99.9%)	49.1% (39.4%-58.8%)
Group4 50-59 years	.704 (.648-.76)	48.6% (40%-57.2%)	92.2% (81.1%-97.8%)	94.4% (86.2%-98.4%)	39.8% (30.9%-49.3%)
Group4 >60 years	.715 (.63-.8)	58.9% (45%-71.9%)	84.1% (69.9%-93.4%)	82.5% (67.2%-92.7%)	61.7% (48.2%-73.9%)
Cut off	4.3 (ms)				
Whole cohort	.674 (.646-.702)	38.4% (33.2%-43.7%)	96.5% (93.2%-98.5%)	94.4% (89.3%-97.6%)	50.2% (45.4%-55%)
Group1 < 30 years	.543 (.485-.602)	8.7% (1.07%-28%)	100% (89.7%-100%)	100% (15.8%-100%)	61.8% (47.7%-74.6%)
Group2 30-39 years	.637 (.575-.699)	27.5% (15.9%-41.7%)	100% (91.8%-100%)	100% (76.8%-100%)	53.8% (42.2%-65%)
Group3 40-49 years	.64 (.587-.692)	29.8% (20.3%-40.7%)	98.2% (90.3%-100%)	96.2% (80.4%-99.9%)	47.8% (38.3%-57.4%)
Group4 50-59 years	.692 (.639-.744)	44.2% (35.8%-52.9%)	94.1% (83.8%-98.8%)	95.3% (86.9%-99%)	38.4% (29.8%-47.5%)
Group4 >60 years	.749 (.671-.827)	58.9% (45%-71.9%)	90.9% (78.3%-97.5%)	89.2% (74.6%-97%)	63.5% (50.4%-75.3%)
Cut off	4.4 (ms)				
Whole cohort	.662 (.635-.69)	35.5% (30.5%-40.8%)	96.9% (93.7%-98.8%)	94.7% (89.4%-97.8%)	49.2% (44.5%-54%)
Group1 < 30 years	.522 (.479-.564)	4.35% (.11%-21.9%)	100% (89.7%-100%)	100% (2.5%-100%)	60.7% (46.8%-73.5%)
Group2 30-39 years	.627 (.567-.688)	25.5% (14.3%-39.6%)	100% (91.8%-100%)	100% (75.3%-100%)	53.1% (41.7%-64.3%)
Group3 40-49 years	.622 (.571-.672)	26.2% (17.2%-36.9%)	98.2% (90.3%-100%)	95.7% (78.1%-99.9%)	46.6% (37.2%-56%)

Group4 50-59 years	.694 (.645-.744)	42.8% (34.4%-51.5%)	96.1% (86.5%-99.5%)	96.7% (88.7%-99.6%)	38.3% (29.8%-47.3%)
Group4 >60 years	.722 (.644-.801)	53.6% (39.7%-67%)	90.9% (78.3%-97.5%)	88.2% (72.5%-96.7%)	60.6% (47.8%-72.4%)
Cut off	4.5 (ms)				
Whole cohort	.647 (.621-.674)	32.1% (27.3%-37.3%)	97.4% (94.3%-99%)	95% (89.3%-98.1%)	48% (43.4%-52.7%)
Group1 < 30 years					
Group2 30-39 years	.618 (.559-.676)	23.5% (12.8%-37.5%)	100% (91.8%-100%)	100% (73.5%-100%)	52.4% (41.1%-63.6%)
Group3 40-49 years	.622 (.571-.672)	26.2% (17.2%-36.9%)	98.2% (90.3%-100%)	95.7% (78.1%-99.9%)	46.6% (37.2%-56%)
Group4 50-59 years	.672 (.624-.721)	38.4% (30.3%-47.1%)	96.1% (86.5%-99.5%)	96.4% (87.5%-99.6%)	36.6% (28.4%-45.3%)
Group4 >60 years	.698 (.622-.774)	46.4% (33%-60.3%)	93.2% (81.3%-98.6%)	89.7% (72.6%-97.8%)	57.7% (45.4%-69.4%)
Cut off	4.6 (ms)				
Whole cohort	.637(.611-.662)	29.5%(24.8%-34.6%)	97.8% (94.9%-99.3%)	95.4% (89.6%-98.5%)	47.2% (42.6%-51.9%)
Group1 < 30 years					
Group2 30-39 years	.588(.535-.641)	17.6%(8.4%-30.9%)	100% (91.8%-100%)	100% (66.4%-100%)	50.6% (39.5%-61.6%)
Group3 40-49 years	.61 (.561-.659)	23.8% (15.2%-34.3%)	98.2% (90.3%-100%)	95.2%(76.2%-99.9%)	45.8%(36.6%-55.2%)
Group4 50-59 years	.679 (.634-.723)	37.7% (29.6%-46.3%)	98% (89.6%-100%)	98.1%(89.9%-100%)	36.8%(28.7%-45.5%)
Group4 >60 years	.671 (.596-.746)	41.1% (28.1%-55%)	93.2% (81.3%-98.6%)	88.5%(69.8%-97.6%)	55.4%(43.4%-67%)
Cut off	4.7 (ms)				
Whole cohort	.625(.6 -.649)	26.7%(22.2%-31.7%)	98.2% (95.5%-99.5%)	95.9%(89.9%-98.9%)	46.4%(41.8%-50.9%)
Group1 < 30 years					
Group2 30-39 years	.569 (.521-.616)	13.7% (5.7%-26.3%)	100% (91.8%-100%)	100%(59%-100%)	49.4%(38.5%-60.4%)
Group3 40-49 years	.586 (.54-.632)	19% (11.3%-29.1%)	98.2%(90.3%-100%)	94.1%(71.3%-99.9%)	44.3%(35.3%-- 53.5%)
Group4 50-59 years	.671 (.627-.716)	36.2% (28.2%-44.8%)	98% (89.6%-100%)	98% (89.6%-100%)	36.2%(28.2%-44.8%)
Group4 >60 years	.665(.594-.736)	37.5% (24.9%-51.5%)	95.5% (84.5%-99.4%)	91.3%(72%-98.9%)	54.5% (42.8%-65.9%)

Cut off	4.8 (ms)				
Whole cohort	.623 (.599-.647)	25.9% (21.4%-30.8%)	98.7% (96.2%-99.7%)	96.8%(91%-99.3%)	46.2% (41.7%-50.7%)
Group1 < 30 years					
Group2 30-39 years	.569 (.521-.616)	13.7% (5.7%-26.3%)	100% (91.8%-100%)	100%(59%-100%)	49.4% (38.5%-60.4%)
Group3 40-49 years	.589 (.548-.63)	17.9% (10.4%-27.7%)	100% (93.5%-100%)	100% (78.2%-100%)	44.4% (35.4%-53.5%)
Group4 50-59 years	.664 (.62-.708)	34.8% (26.9%-43.4%)	98% (89.6%-100%)	98% (89.1%-99.9%)	35.7%(27.8%-44.2%)
Group4 >60 years	.665 (.594-.736)	37.5% (24.9%-51.5%)	95.5% (84.5%-99.4%)	91.3%(72%-98.9%)	54.5%(42.8%-65.9%)
Cut off	4.9 (ms)				
Whole cohort	.621(.598-.644)	24.7%(20.3%-29.6%)	99.6% (97.6%-100%)	98.9% (93.8%-100%)	46% (41.6%-50.6%)
Group1 < 30 years					
Group2 30-39 years	.569(.521-.616)	13.7%(5.7%-26.3%)	100% (91.8%-100%)	100% (59%-100%)	49.4% (38.5%-60.4%)
Group3 40-49 years	.577(.538-.616)	15.5%(8.51%-25%)	100%(93.5%-100%)	100% (75.3%-100%)	43.7%(34.8%)-52.8%
Group4 50-59 years	.66(.616-.705)	34.1% (26.2%-42.6%)	98% (89.6%-100%)	97.9%(88.9%-99.9%)	35.5%(27.6%-44%)
Group4 >60 years	.679 (.615-.742)	35.7%(23.4%-49.6%)	100% (92%-100%)	100%(83.2%-100%)	55% (43.5%-66.2%)
Cut off	5.0 (ms)				
Whole cohort	.615(.593-.637)	23% (18.7%-27.8%)	100%(98.4%-100%)	100% (95.5%-100%)	45.6%(41.1%-50.1%)
Group1 < 30 years					
Group2 30-39 years	.559 (.514-.603)	11.8%(4.44%-23.9%)	100% (91.8%-100%)	100%(54.1%-100%)	48.9%(38.1%-59.8%)
Group3 40-49 years	.577(.538-.616)	15.5%(8.51%-25%)	100% (93.5%-100%)	100% (75.3%-100%)	43.7%(34.8%-52.8%)
Group4 50-59 years	.659 (.62-.698)	31.9%(24.2%-40.4%)	100% (93%-100%)	100% (92%-100%)	35.2% (27.4%-43.5%)
Group4 >60 years	.661 (.599-.722)	32.1% (20.3%-46%)	100%(92%-100%)	100%(81.5%-100%)	53.7%(42.3%-64.7%)

**********************************palmdiff**********************************

Diagnostic accuracy of palmdiff, All cutoff values , all age groups

Age group	ROC	Sensitivity	Specificity	PPV	NPV
Cut off	0.4				
Whole cohort	.78(.744-.815)	74.1%(68.9%-78.9%)	81.8% (75.9%-86.8%)	86.1% (81.4%-90%)	67.6% (61.4%-73.3%)
Group1 < 30 years	.622(.522-.721)	27.3%(10.7%-50.2%)	97.1%(84.7%-99.9%)	85.7% (42.1%-99.6%)	67.3%(52.5%-80.1%)
Group2 30-39 years	.752(.668-.836)	60.4%(45.3%-74.2%)	90% (76.3%-97.2%)	87.9% (71.8%-96.6%)	65.5%(51.4%-77.8%)
Group3 40-49 years	.796(.727-.864)	71.6%(60.5%-81.1%)	87.5% (74.8%-95.3%)	90.6% (80.7%-96.5%)	64.6% (51.8%-76.1%)
Group4 50-59 years	.763(.688-.838)	84.5%(76.6%-90.5%)	68.1%(52.9%-80.9%)	86.7% (79.1%-92.4%)	64%(49.2%-77.1%)
Group4 >60 years	.79 (.705-.875)	88% (75.7%-95.5%)	70%(53.5%-83.4%)	78.6%(65.6%-88.4%)	82.4% (65.5%-93.2%)
Cut off	0.5				
Whole cohort	.752(.718-.787)	62.5% (56.9%-67.8%)	88%(82.9%-92.1%)	88.8%(83.9%-92.6%)	60.7%(55%-66.3%)
Group1 < 30 years	.614(.524-.703)	22.7%(7.82%-45.4%)	100%(89.7%-100%)	100%(47.8%-100%)	66.7%(52.1%-79.2%)
Group2 30-39 years	.737(.662-.813)	50% (35.2%-64.8%)	97.5%(86.8%-99.9%)	96% (79.6%-99.9%)	61.9%(48.8%-73.9%)
Group3 40-49 years	.779 (.714-.845)	64.2%(52.8%-74.6%)	91.7%(80%-97.7%)	92.9%(82.7%-98%)	60.3% (48.1%-71.5%)
Group4 50-59 years	.726(.652-.799)	66.4%(57%-74.9%)	78.7%(64.3%-89.3%)	88.5%(79.9%-94.3%)	48.7% (37%-60.4%)
Group4 >60 years	775 (.687-.863)	80% (66.3%- 90%)	75%(58.8%-87.3%)	80% (66.3%-90%)	75%(58.8%-87.3%)
Cut off	0.6				
Whole cohort	.748(.715-.781)	57.7%(52.1%-63.2%)	91.9%(87.3%-95.2%)	91.5%(86.7%-95%)	58.9%(53.3%-64.3%)
Group1 < 30 years	.591(.508-.673)	18.2%(5.19%-40.3%)	100%(89.7%-100%)	100%(39.8%-100%)	65.4%(50.9%-78%)
Group2 30-39 years	.706(.631-.781)	43.8%(29.5%-58.8%)	97.5% (86.8%-99.9%)	95.5%(77.2%-99.9%)	59.1% (46.3%-71%)
Group3 40-49 years	.771(.707-.835)	60.5%(49%-71.2%)	93.8%(82.8%-98.7%)	94.2%(84.1%-98.8%)	58.4%(46.6%-69.6%)
Group4 50-59 years	.727(.659-.795)	60.3% (50.8%-69.3%)	85.1%(71.7%-93.8%)	90.9%(82.2%-96.3%)	46.5% (35.7%-57.6%)

Group4 >60 years	.815(.734-.896)	78% (64% -88.5%)	85% (70.2%-94.3%)	86.7%(73.2%-94.9%)	75.6% (60.5%-87.1%)
Cut off	0.7				
Whole cohort	.72 (.687-.752)	51.1% (45.5%-56.7%)	92.8%(88.4%-95.9%)	91.5%(86.4%-95.2%)	55.6%(50.2%-60.9%)
Group1 < 30 years	.591(.508-.673)	18.2%(5.19%-40.3%)	100%(89.7%-100%)	100% (39.8%-100%)	65.4%(50.9%-78%)
Group2 30-39 years	.654 (.582-.726)	33.3%(20.4%-48.4%)	97.5% (86.8%-99.9%)	94.1% (71.3%-99.9%)	54.9% (42.7%-66.8%)
Group3 40-49 years	.751(.689-.812)	54.3%(42.9%-65.4%)	95.8% (85.7%-99.5%)	95.7%(85.2%-99.5%)	55.4% (44.1%-66.3%)
Group4 50-59 years	.699 (.633-.765)	52.6% (43.1%-61.9%)	87.2%(74.3%-95.2%)	91% (81.5%-96.6%)	42.7%(32.7%-53.2%)
Group4 >60 years	.795 (.712-.878)	74% (59.7%-85.4%)	85% (70.2%-94.3%)	86% (72.1%-94.7%)	72.3% (57.4%-84.4%)
Cut off	0.8				
Whole cohort	.698(.667 -.73)	45.4%(39.9%-51.1%)	94.3%(90.2%-97%)	92.3%(86.9%-96%)	53.2%(48%-58.4%)
Group1 < 30 years	.568 (.495-.642)	13.6%(2.91%-34.9%)	100%(89.7%-100%)	100%(29.2%-100%)	64.2%(49.8%-76.9%)
Group2 30-39 years	.667 (.599-.734)	33.3%(20.4%-48.4%)	100% (91.2%-100%)	100% (79.4%-100%)	55.6%(43.4%-67.3%)
Group3 40-49 years	.732 (.67 -.794)	50.6% (39.3%-61.9%)	95.8%(85.7%-99.5%)	95.3%(84.2%-99.4%)	53.5% (42.4%-64.3%)
Group4 50-59 years	.68(.616-.743)	46.6% (37.2%-56%)	89.4% (76.9%-96.5%)	91.5%(81.3%-97.2%)	40.4%(30.9%-50.5%)
Group4 >60 years	.738 (.651-.824)	60%(45.2%-73.6%)	87.5% (73.2%-95.8%)	85.7%(69.7%-95.2%)	63.6%(49.6%-76.2%)
Cut off	0.9				
Whole cohort	.684(.654-.714)	40.7% (35.2%-46.3%)	96.2%(92.6%-98.3%_	94.2%(88.8%-97.4%)	51.7% (46.6%-56.7%)
Group1 < 30 years	.568(.495-.642)	13.6% (2.91%-34.9%)	100%(89.7%-100%)	100% (29.2%-100%)	64.2% (49.8%-76.9%)
Group2 30-39 years	.646 (.581-.711)	29.2%(17%-44.1%)	100% (91.2%-100%)	100% (76.8%-100%)	54.1%(42.1%-65.7%)
Group3 40-49 years	.708(.646-.769)	45.7%(34.6%-57.1%)	95.8% (85.7%-99.5%)	94.9%(82.7%-99.4%)	51.1%(40.3%-61.8%)
Group4 50-59 years	.686(.632-.739)	41.4%(32.3%-50.9%)	95.7%(85.5%-99.5%)	96% (86.3%-99.5%)	39.8%(30.7%-49.5%)
Group4 >60 years	.72 (.636-.804)	54% (39.3%-68.2%)	90% (76.3%-97.2%)	87.1%(70.2%-96.4%)	61% (47.4%-73.5%)
Cut off	1.0				

Whole cohort	.665 (.637-.694)	36% (30.7%-41.5%)	97.1% (93.9%-98.9%)	95%(89.4%-98.1%)	50% (45% -55%)
Group1 < 30 years	.568(.495-.642)	13.6%(2.91%-34.9%)	100%(89.7%-100%)	100%(29.2%-100%)	64.2%(49.8%-76.9%)
Group2 30-39 years	.635(.572-.699)	27.1% (15.3%-41.8%)	100% (91.2%-100%)	100% (75.3%-100%)	53.3% (41.4%-64.9%)
Group3 40-49 years	.681 (.624-.738)	38.3%(27.7%-49.7%)	97.9%(88.9%-99.9%)	96.9%(83.8%-99.9%)	48.5%(38.2%-58.8%)
Group4 50-59 years	.668(.615-.721)	37.9%(29.1%-47.4%)	95.7%(85.5%-99.5%)	95.7%(85.2%-99.5%)	38.5%(29.6%-47.9%)
Group4 >60 years	.693(.611-.774)	46% (31.8%-60.7%)	92.5%(79.6%-98.4%)	88.5%(69.8%-97.6%)	57.8%(44.8%-70.1%)
Cut off	1.1				
Whole cohort	.648 (.62-.676)	32.5% (27.4%-38%)	97.1%(93.9%-98.9%)	94.5%(88.4%-98%)	48.7% (43.8%-53.6%)
Group1 < 30 years	.545 (.484-.607)	9.09%(1.12%-29.2%)	100%(89.7%-100%)	100% (15.8%-100%)	63% (48.7%-75.7%)
Group2 30-39 years	.604(.546-.662)	20.8% (10.5%-35%)	100%(91.2%-100%)	100% (69.2%-100%)	51.3%(39.7%-62.8%)
Group3 40-49 years	.662(.606-.718)	34.6% (24.3%-46%)	97.9%(88.9%-99.9%)	96.6% (82.2%-99.9%)	47% (36.9%-57.2%)
Group4 50-59 years	.664 (.611-.717)	37.1% (28.3%-46.5%)	95.7%(85.5%-99.5%)	95.6%(84.9%-99.5%)	38.1%(29.4%-47.5%)
Group4 >60 years	.663(.582-.743)	40% (26.4%-54.8%)	92.5%(79.6%-98.4%)	87% (66.4%-97.2%)	55.2% (42.6%-67.4%)
Cut off	1.2				
Whole cohort	.634(.607-.661)	28.7%(23.8%-34%)	98.1%(95.2%-99.5%)	95.8%(89.6%-98.8%)	47.6% (42.8%-52.4%)
Group1 < 30 years	.523 (.478-.567)	4.55%(.115%-22.8%)	100% (89.7%-100%)	100%(2.5%-100%)	61.8%(47.7%-74.6%)
Group2 30-39 years	.604 (.546-.662)	20.8%(10.5%-35%)	100%(91.2%-100%)	100% (69.2%-100%)	51.3% (39.7%-62.8%)
Group3 40-49 years	.644 (.589-.698)	30.9% (21.1%-42.1%)	97.9%(88.9%-99.9%)	96.2%(80.4%-99.9%)	45.6%(35.8%-55.7%)
Group4 50-59 years	.653(.605-.701)	32.8%(24.3%-42.1%)	97.9% (88.7%-99.9%)	97.4%(86.5%-99.9%)	37.1%(28.6%-46.2%)
Group4 >60 years	.645(.57-.72)	34% (21.2%-48.8%)	95% (83.1%-99.4%)	89.5%(66.9%-98.7%)	53.5% (41.3%-65.5%)
Cut off	1.3				
Whole cohort	.621 (.595-.647)	26.2%(21.4%-31.4%)	98.1% (95.2%-99.5%)	95.4%(88.6%-98.7%)	46.7%(42%-51.5%)
Group1 < 30 years					

Group2 30-39 years	.594(.538-.65)	18.8% (8.95%-32.6%)	100% (91.2%-100%)	100%(66.4%-100%)	50.6%(39.1%-62.1%)
Group3 40-49 years	.625(.573-.678)	27.2% (17.9%-38.2%)	97.9%(88.9%-99.9%)	95.7% (78.1%-99.9%)	44.3%(34.7%-54.3%)
Group4 50-59 years	.64 (.593-.687)	30.2%(22%-39.4%)	97.9%(88.7%-99.9%)	97.2% (85.5%-99.9%)	36.2%(27.9%-45.2%)
Group4 >60 years	.645 (.57 - .72)	34% (21.2%-48.8%)	95%(83.1%-99.4%)	89.5%(66.9%-98.7%)	53.5%(41.3%-65.5%)
Cut off	1.4				
Whole cohort	.605 (.58 - .629)	22.4% (17.9%-27.4%)	98.6%(95.9%-99.7%)	95.9%(88.6%-99.2%)	45.6%(40.9%-50.3%)
Group1 < 30 years					
Group2 30-39 years	.583(.53 - .637)	16.7%(7.48%-30.2%)	100%(91.2%-100%)	100%(63.1%-100%)	50% (38.6%-61.4%)
Group3 40-49 years	.595(.545-.644)	21% (12.7%-31.5%)	97.9% (88.9%-99.9%)	94.4% (72.7%-99.9%)	42.3%(33%-52.1%)
Group4 50-59 years	.619 (.574-.664)	25.9% (18.2%-34.8%)	97.9% (88.7%-99.9%)	96.8%(83.3%-99.9%)	34.8% (26.8%-43.6%)
Group4 >60 years	.647(.578-.717)	32% (19.5%-46.7%)	97.5% (86.8%-99.9%)	94.1%(71.3%-99.9%)	53.4% (41.4%-65.2%)

********************************Thumbdiff********************************

Diagnostic accuracy of thumbdiff, All cutoff values , all age groups

Age group	ROC	Sensitivity	Specificity	PPV	NPV
Cut off	0.5				
Whole cohort	.715(.676-.753)	84.4%(79.9%-88.2%)	58.6%(51.7%-65.3%)	75.2%(70.4%-79.6%)	71.6% (64.3%-78.1%)
Group1 < 30 years	.728 (.603-.853)	68.2%(45.1%-86.1%)	77.4% (58.9%-90.4%)	68.2% (45.1%-86.1%)	77.4%(58.9%-90.4%)
Group2 30-39 years	.673(.574-.771)	72% (57.5%-83.8%)	62.5%(45.8%-77.3%)	70.6% (56.2%-82.5%)	64.1%(47.2%-78.8%)
Group3 40-49 years	.719(.64-.797)	81.5%(71.3%-89.2%)	62.3%(47.9%-75.2%)	76.7%(66.4%-85.2%)	68.8%(53.7%-81.3%)
Group4 50-59 years	.728 (.652-.803)	90.4% (83.5%-95.1%)	55.1%(40.2%-69.3%)	82.5% (74.8%-88.7%)	71.1%(54.1%-84.6%)
Group4 >60 years	.674(.592-.755)	94.2% (84.1%-98.8%)	40.5% (25.6%-56.7%)	66.2%(54.3%-76.8%)	85% (62.1%-96.8%)
Cut off	0.6				
Whole cohort	.746(.708-.784)	79.4% (74.5%-83.7%)	69.8%(63.2%-75.8%)	79.6%(74.8%-83.9%)	69.4% (62.8%-75.5%)
Group1 < 30 years	.793(.68 -.905)	68.2%(45.1%-86.1%)	90.3% (74.2%-98%)	83.3%(58.6%-96.4%)	80% (63.1%-91.6%)
Group2 30-39 years	.672 (.575-.77)	62% (47.2%-75.3%)	72.5%(56.1%-85.4%)	73.8% (58%-86.1%)	60.4%(45.3%-74.2%)
Group3 40-49 years	.732(.654-.81)	76.5%(65.8%-85.2%)	69.8%(55.7%-81.7%)	79.5%(68.8%-87.8%)	66.1% (52.2%-78.2%)
Group4 50-59 years	.769 (.695-.842)	84.3%(76.4%-90.5%)	69.4%(54.6%-81.7%)	86.6% (78.9%-92.3%)	65.4% (50.9%-78%)
Group4 >60 years	.733(.65-.816)	94.2% (84.1%-98.8%)	52.4% (36.4%-68%)	71% (58.8%-81.3%)	88%(68.8%-97.5%)
Cut off	0.7				
Whole cohort	.745(.708-.782)	70% (64.7%- 75%)	79.1% (73%-84.3%)	83.3%(78.3%-87.5%)	63.9%(57.8%-69.7%)
Group1 < 30 years	.688 (.579-.798)	40.9%(20.7%-63.6%)	96.8%(83.3%-99.9%)	90% (55.5%-99.7%)	69.8%(53.9%-82.8%)
Group2 30-39 years	.693(.601-.784)	56% (41.3%- 70%)	82.5%(67.2%-92.7%)	80%(63.1%-91.6%)	60% (45.9%-73%)
Group3 40-49 years	.77 (.7 -.84)	69.1%(57.9%-78.9%)	84.9%(72.4%-93.3%)	87.5%(76.8%-94.4%)	64.3% (51.9%-75.4%)
Group4 50-59 years	.756(.683-.828)	75.7%(66.8%-83.2%)	75.5%(61.1%-86.7%)	87.9%(79.8%-93.6%)	56.9%(44% -69.2%)
Group4 >60 years	.721(.631 -.811)	84.6%(71.9%-93.1%)	59.5% (43.3%-74.4%)	72.1% (59.2%-82.9%)	75.8%(57.7%-88.9%)

Cut off	0.8				
Whole cohort	.733(.697-.77)	63.4%(57.9%-68.7%)	83.3%(77.6%-88%)	84.9%(79.8%-89.2%)	60.5% (54.7%-66.1%)
Group1 < 30 years	.682(.579-.785)	36.4%(17.2%-59.3%)	100%(88.8%-100%)	100% (63.1% -100%)	68.9%(53.4%-81.8%)
Group2 30-39 years	.697 (.61-.785)	52% (37.4%-66.3%)	87.5%(73.2%-95.8%)	83.9%(66.3%-94.5%)	59.3%(45.7%-71.9%)
Group3 40-49 years	.752(.684-.821)	61.7%(50.3%-72.3%)	88.7%(77%-95.7%)	89.3% (78.1%-96%)	60.3%(48.5%-71.2%)
Group4 50-59 years	.728 (.657-.8)	66.1%(56.7%-74.7%)	79.6%(65.7%-89.8%)	88.4%(79.7%-94.3%)	50% (38.5%-61.5%)
Group4 >60 years	.735(.645-.825)	82.7%(69.7%-91.8%)	64.3%(48%-78.4%)	74.1% (61%-84.7%)	75% (57.8%-87.9%)
Cut off	0.9				
Whole cohort	.719 (.684-.755)	57.8%(52.2%-63.3%)	86% (80.7%-90.4%)	86% (80.7%-90.4%)	57.8% (52.2%-63.3%)
Group1 < 30 years	.659(.559-.759)	31.8%(13.9%-54.9%)	100% (88.8%-100%)	100%(59%-100%)	67.4%(52%-80.5%)
Group2 30-39 years	.702 (.621-.784)	48%(33.7%-62.6%)	92.5% (79.6%-98.4%)	88.9% (70.8%-97.6%)	58.7%(45.6%-71%)
Group3 40-49 years	.727 (.658-.797)	56.8% (45.3%-67.8%)	88.7%(77%-95.7%)	88.5%(76.6%-95.6%)	57.3%(45.9%-68.2%)
Group4 50-59 years	.718 (.649-.787)	60% (50.4%- 69%)	83.7%(70.3%-92.7%)	89.6%(80.6%-95.4%)	47.1%(36.3%-58.1%)
Group4 >60 years	.72 (.628-.813)	75% (61.1%- 86%)	69%(52.9%-82.4%)	75% (61.1%-86%)	69%(52.9%-82.4%)
Cut off	1.0				
Whole cohort	.711(.677-.744)	51.9% (46.2%-57.5%)	90.2%(85.5%-93.9%)	88.8%(83.3%-92.9%)	55.7%(50.4%-61%)
Group1 < 30 years	.636(.541-.732)	27.3%(10.7%-50.2%)	100%(88.8%-100%)	100% (54.1%-100%)	66% (50.7%-79.1%)
Group2 30-39 years	.682(.602-.763)	44%(30%- 58.7%)	92.5%(79.6%-98.4%)	88% (68.8%-97.5%)	56.9%(44%-69.2%)
Group3 40-49 years	.715(.65-.781)	50.6%(39.3%-61.9%)	92.5%(81.8%-97.9%)	91.1%(78.8%-97.5%)	55.1%(44.1%-65.6%)
Group4 50-59 years	.724 (.664-.784)	53% (43.5%-62.4%)	91.8%(80.4%-97.7%)	93.8%(85%-98.3%)	45.5%(35.4%-55.8%)
Group4 >60 years	.727(.636-.818)	69.2%(54.9%-81.3%)	76.2%(60.5%-87.9%)	78.3%(63.6%-89.1%)	66.7% (51.6%-79.6%)
Cut off	1.1				
Whole cohort	.695(.663-.727)	45.9%(40.4%-51.6%)	93% (88.8%-96%)	90.7% (85.2%-94.7%)	53.6%(48.4%-58.8%)
Group1 < 30 years	.636 (.541-.732)	27.3%(10.7%-50.2%)	100%(88.8%-100%)	100% (54.1%-100%)	66% (50.7%-79.1%)

Group2 30-39 years	.658 (.587-.728)	34% (21.2%-48.8%)	97.5% (86.8%-99.9%)	94.4%(72.7%-99.9%)	54.2%(42%-66%)
Group3 40-49 years	.678 (.613-.743)	43.2%(32.2%-54.7%)	92.5%(81.8%-97.9%)	89.7%(75.8%-97.1%)	51.6%(41.1%-62%)
Group4 50-59 years	.713 (.656-.77)	48.7% (39.3%-58.2%)	93.9%(83.1%-98.7%)	94.9% (85.9%-98.9%)	43.8% (34.1%-53.8%)
Group4 >60 years	.734(.647-.821)	63.5%(49%-76.4%)	83.3%(68.6%-93%)	82.5%(67.2%-92.7%)	64.8%(50.6%-77.3%)
Cut off	1.2				
Whole cohort	.681(.65-.711)	41.3%(35.8%-46.9%)	94.9%(91%-97.4%)	92.3%(86.7%-96.1%)	52% (47%-57.1%)
Group1 < 30 years	.614(.524-.703)	22.7%(7.82%-45.4%)	100%(88.8%-100%)	100%(47.8%-100%)	64.6% (49.5%-77.8%)
Group2 30-39 years	.638(.569-.706)	30% (17.9%-44.6%)	97.5%(86.8%-99.9%)	93.8%(69.8%-99.8%)	52.7%(40.7%-64.4%)
Group3 40-49 years	.672(.613-.732)	38.3%(27.7%-49.7%)	96.2%(87%-99.5%)	93.9%(79.8%-99.3%)	50.5%(40.4%-60.6%)
Group4 50-59 years	.706 (.652-.759)	45.2%(35.9%-54.8%)	95.9%(86%-99.5%)	96.3% (87.3%-99.5%)	42.7% (33.3%-52.5%)
Group4 >60 years	.707 (.621-.794)	55.8%(41.3%-69.5%)	85.7%(71.5%-94.6%)	82.9% (66.4%-93.4%)	61% (47.4%-73.5%)
Cut off	1.3				
Whole cohort	.67(.64- .7)	38.1%(32.8%-43.7%)	95.8%(92.2%-98.1%)	93.1%(87.4%-96.8%)	51% (46% -56%)
Group1 < 30 years	.591(.508-.673)	18.2%(5.19%-40.3%)	100%(88.8%-100%)	100% (39.8%-100%)	63.3%(48.3%-76.6%)
Group2 30-39 years	.607 (.543-.672)	24% (13.1%-38.2%)	97.5%(86.8%-99.9%)	92.3%(64%-99.8%)	50.6%(39%-62.2%)
Group3 40-49 years	.666(.607-.725)	37% (26.6%-48.5%)	96.2%(87%-99.5%)	93.8%(79.2%-99.2%)	50% (39.9%-60.1%)
Group4 50-59 years	.698 (.649-.748)	41.7%(32.6%-51.3%)	98% (89.1%-99.9%)	98% (89.1%-99.9%)	41.7%(32.6%-51.3%)
Group4 >60 years	.71 (.625-.794)	53.8% (39.5%-67.8%)	88.1% (74.4%-96%)	84.8% (68.1%-94.9%)	60.7%(47.3%-72.9%)
Cut off	1.4				
Whole cohort	.667(.639-.696)	36.3%(31%-41.8%)	97.2%(94%-99%)	95.1%(89.6%-98.2%)	50.6%(45.7%-55.5%)
Group1 < 30 years	.591(.508-.673)	18.2%(5.19%-40.3%)	100%(88.8%-100%)	100%(39.8%-100%)	63.3%(48.3%-76.6%)
Group2 30-39 years	.62(.56- .68)	24% (13.1%-38.2%)	100%(91.2%-100%)	100%(73.5%-100%)	51.3% (39.7%-62.8%)
Group3 40-49 years	.657(.602-.712)	33.3%(23.2%-44.7%)	98.1%(89.9%-100%)	96.4%(81.7%-99.9%)	49.1%(39.2%-59%)

Group4 50-59 years	.69(.641-.739)	40% (31% -49.6%)	98%(89.1%-99.9%)	97.9%(88.7%-99.9%)	41% (32% -50.5%)
Group4 >60 years	.712 .63 .794	51.9% 37.6% 66%	90.5% 77.4% 97.3%	87.1% 70.2% 96.4%	60.3% 47.2% 72.4%
Cut off	1.5				
Whole cohort	.657 .63 .684	32.8% 27.7% 38.3%	98.6% 96% 99.7%	97.2% 92.1% 99.4%	49.6% 44.8% 54.5%
Group1 < 30 years	.568 .495 .642	13.6% 2.91% 34.9%	100% 88.8% 100%	100% 29.2% 100%	62% 47.2% 75.3%
Group2 30-39 years	.6 .544 .656	20% 10% 33.7%	100% 91.2% 100%	100% 69.2% 100%	50% 38.6% 61.4%
Group3 40-49 years	.648 .598 .698	29.6% 20% 40.8%	100% 93.3% 100%	100% 85.8% 100%	48.2% 38.6% 57.9%
Group4 50-59 years	.681 .632 .73	38.3% 29.4% 47.8%	98% 89.1% 99.9%	97.8% 88.2% 99.9%	40.3% 31.4% 49.7%
Group4 >60 years	.707 .631 .783	46.2% 32.2% 60.5%	95.2% 83.8% 99.4%	92.3% 74.9% 99.1%	58.8% 46.2% 70.6%

**********************************Ringdiff**********************************

Diagnostic accuracy of ringdiff, All cutoff values , all age groups

Age group	ROC	Sensitivity	Specificity	PPV	NPV
Cut off	0.5				
Whole cohort	.724 (.686-.762)	61.6%(55.7%-67.2%)	83.3% (77.5%-88%)	83.6%(77.9%-88.3%)	61.1%(55.1%-66.7%)
Group1 < 30 years	.643 (.539-.748)	31.8% (13.9%-54.9%)	96.9%(83.8%-99.9%)	87.5% (47.3%-99.7%)	67.4% (52%-80.5%)
Group2 30-39 years	.697 (.613-.781)	46.7% (31.7%-62.1%)	92.7%(80.1%-98.5%)	87.5%(67.6%-97.3%)	61.3%(48.1%-73.4%)
Group3 40-49 years	.769 (.701-.837)	62.2% (50.1%-73.2%)	91.7%(80%-97.7%)	92% (80.8%-97.8%)	61.1%(48.9%-72.4%)
Group4 50-59 years	.69 (.613-.768)	66.7%(56.9%-75.4%)	71.4%(56.7%-83.4%)	83.7% (74.2%-90.8%)	49.3%(37.2%-61.4%)
Group4 >60 years	.733 (.636-.831)	80% (64.4%-90.9%)	66.7%(49.8%-80.9%)	71.1% (55.7%-83.6%)	76.5%(58.8%-89.3%)
Cut off	0.6				
Whole cohort	.712 (.676-.748)	54.3% (48.4%-60.2%)	88%(82.9%-92.1%)	86.3%(80.4%-90.9%)	58.2% (52.6%-63.7%)
Group1 < 30 years	.598 (.503-.693)	22.7%(7.82%-45.4%)	96.9%(83.8%-99.9%)	83.3%(35.9%-99.6%)	64.6% (49.5%-77.8%)
Group2 30-39 years	.71 (.633-.787)	44.4%(29.6%-60%)	97.6%(87.1%-99.9%)	95.2% (76.2%-99.9%)	61.5% (48.6%-73.3%)
Group3 40-49 years	.719 (.652-.786)	50% (38.1%-61.9%)	93.8%(82.8%-98.7%)	92.5%(79.6%-98.4%)	54.9%(43.5%-65.9%)
Group4 50-59 years	.694 (.621-.768)	59.3%(49.4%-68.6%)	79.6%(65.7%-89.8%)	86.5%(76.5%-93.3%)	47% (35.9%-58.3%)
Group4 >60 years	.759(.664-.855)	77.5% (61.5%-89.2%)	74.4%(57.9%-87%)	75.6%(59.7%-87.6%)	76.3%(59.8%-88.6%)
Cut off	0.7				
Whole cohort	.698 (.663-.734)	50.2% (44.3%-56.1%)	89.5% (84.5%-93.3%)	86.8%(80.7% -91.6%)	56.5%(51%-61.9%)
Group1 < 30 years	.614(.524-.703)	22.7% (7.82%-45.4%)	100%(89.1%-100%)	100% (47.8%-100%)	65.3% (50.4%-78.3%)
Group2 30-39 years	.677 (.601-.752)	37.8%(23.8%-53.5%)	97.6% (87.1%-99.9%)	94.4% (72.7%-99.9%)	58.8%(46.2%-70.6%)
Group3 40-49 years	.685 (.618-.751)	43.2%(31.8%-55.3%)	93.8%(82.8%-98.7%)	91.4% (76.9%-98.2%)	51.7%(40.8%-62.6%)
Group4 50-59 years	.685 (.611-.759)	57.4%(47.5%-66.9%)	79.6%(65.7%-89.8%)	86.1% (75.9%-93.1%)	45.9%(35%-57%)
Group4 >60 years	.76 (.665-.855)	72.5%(56.1%-85.4%)	79.5%(63.5%-90.7%)	78.4%(61.8%-90.2%)	73.8%(58%-86.1%)

Cut off	0.8				
Whole cohort	.685(.65-.719)	46% (40.2%- 52%)	90.9%(86.2%-94.4%)	87.5%(81.2%-92.3%)	54.9%(49.5%-60.2%)
Group1 < 30 years	.591(.508-.673)	18.2%(5.19%-40.3%)	100%(89.1%-100%)	100% (39.8%-100%)	64% (49.2%-77.1%)
Group2 30-39 years	.677 (.601-.752)	37.8%(23.8%-53.5%)	97.6%(87.1%-99.9%)	94.4%(72.7%-99.9%)	58.8% (46.2%-70.6%)
Group3 40-49 years	.675 (.612-.738)	39.2%(28%-51.2%)	95.8%(85.7%-99.5%)	93.5%(78.6%-99.2%)	50.5%(39.9%-61.2%)
Group4 50-59 years	.678 (.607-.748)	51.9%(42%-61.6%)	83.7%(70.3%-92.7%)	87.5% (76.8%-94.4%)	44.1%(33.8%-54.8%)
Group4 >60 years	.735 (.637-.833)	67.5%(50.9%-81.4%)	79.5%(63.5%-90.7%)	77.1%(59.9%-89.6%)	70.5%(54.8%-83.2%)
Cut off	0.9				
Whole cohort	.672(.638-.705)	41.5% (35.8%-47.4%)	92.8% (88.4%-95.9%)	88.9%(82.3%-93.6%)	53.4% (48.2%-58.7%)
Group1 < 30 years	.591 (.508-.673)	18.2% (5.19%-40.3%)	100% (89.1%-100%)	100% (39.8%-100%)	64% (49.2%-77.1%)
Group2 30-39 years	.666 (.591-.74)	35.6% (21.9%-51.2%)	97.6%(87.1%-99.9%)	94.1%(71.3%-99.9%)	58% (45.5%-69.8%)
Group3 40-49 years	.665 (.607-.724)	35.1%(24.4%-47.1%)	97.9%(88.9%-99.9%)	96.3%(81%-99.9%)	49.5% (39.1%-59.9%)
Group4 50-59 years	.675 (.609-.741)	47.2% (37.5%-57.1%)	87.8% (75.2%-95.4%)	89.5%(78.5%-96%)	43% (33.1%-53.3%)
Group4 >60 years	.698 (.599-.796)	57.5% (40.9%-73%)	82.1%(66.5%-92.5%)	76.7%(57.7%-90.1%)	65.3%(50.4%-78.3%)
Cut off	1.0				
Whole cohort	.673(.641-.705)	39.8% (34.1%-45.7%)	94.7%(90.8%-97.3%)	91.3% (84.9%-95.6%)	53.2%(48%-58.4%)
Group1 < 30 years	.591(.508-.673)	18.2%(5.19%-40.3%)	100%(89.1%-100%)	100%(39.8%-100%)	64%(49.2%-77.1%)
Group2 30-39 years	.667 (.597-.736)	33.3%(20% -49%)	100%(91.4%-100%)	100%(78.2%-100%)	57.7%(45.4%-69.4%)
Group3 40-49 years	.669 (.615-.723)	33.8%(23.2%-45.7%)	100%(92.6%-100%)	100% (86.3%-100%)	49.5%(39.2%-59.8%)
Group4 50-59 years	.686 (.625-.747)	45.4%(35.8%-55.2%)	91.8%(80.4%-97.7%)	92.5%(81.8%-97.9%)	43.3%(33.6%-53.3%)
Group4 >60 years	.685 (.586-.784)	55% (38.5%-70.7%)	82.1%(66.5%-92.5%)	75.9%(56.5%-89.7%)	64% (49.2%-77.1%)
Cut off	1.1				
Whole cohort	.665 (.634-.696)	37.4%(31.8%-43.2%)	95.7% (92%-98%)	92.3% (85.9%-96.4%)	52.5%(47.3%-57.6%)
Group1 < 30 years	.591 (.508-.673)	18.2% (5.19%-40.3%)	100%(89.1%-100%)	100%(39.8%-100%)	64% (49.2%-77.1%)

Group2 30-39 years	.667 (.597-.736)	33.3%(20% -49%)	100%(91.4%-100%)	100%(78.2%-100%)	57.7%(45.4%-69.4%)
Group3 40-49 years	.655(.602-.708)	31.1%(20.8%-42.9%)	100% (92.6%-100%)	100% (85.2%-100%)	48.5%(38.3%-58.7%)
Group4 50-59 years	.687(.629-.745)	43.5%(34%-53.4%)	93.9%(83.1%-98.7%)	94% (83.5%-98.7%)	43% (33.5%-52.9%)
Group4 >60 years	.661 (.563-.758)	47.5% (31.5%-63.9%)	84.6%(69.5%-94.1%)	76% (54.9%-90.6%)	61.1%(46.9%-74.1%)
Cut off	1.2				
Whole cohort	.653 (.624-.683)	33.6%(28.1%-39.3%)	97.1%(93.9%-98.9%)	94.2%(87.8%-97.8%)	51.4%(46.3%-56.4%)
Group1 < 30 years	.591 (.508-.673)	18.2%(5.19%-40.3%)	100%(89.1%-100%)	100% (39.8%-100%)	64% (49.2%-77.1%)
Group2 30-39 years	.633(.568-.699)	26.7% (14.6%-41.9%)	100%(91.4%-100%)	100% (73.5%-100%)	55.4%(43.4%-67%)
Group3 40-49 years	.642 (.59-.694)	28.4%(18.5%-40.1%)	100%(92.6%-100%)	100% (83.9%-100%)	47.5%(37.5%-57.7%)
Group4 50-59 years	.668 (.611-.726)	39.8%(30.5%-49.7%)	93.9% (83.1%-98.7%)	93.5% (82.1%-98.6%)	41.4%(32.2%-51.2%)
Group4 >60 years	.674(.586-.762)	42.5%(27%-59.1%)	92.3%(79.1%-98.4%)	85% (62.1%-96.8%)	61% (47.4%-73.5%)
Cut off	1.3				
Whole cohort	.644(.615-.672)	31.1% (25.8%-36.8%)	97.6% (94.5%-99.2%)	94.7%(88.1%-98.3%)	50.6%(45.6%-55.6%)
Group1 < 30 years	.591 (.508-.673)	18.2%(5.19%-40.3%)	100%(89.1%-100%)	100%(39.8%-100%)	64% (49.2%-77.1%)
Group2 30-39 years	.622 (.559-.686)	24.4%(12.9%-39.5%)	100% (91.4%-100%)	100%(71.5%-100%)	54.7%(42.7%-66.2%)
Group3 40-49 years	.642 (.59-.694)	28.4%(18.5%-40.1%)	100%(92.6%-100%)	100%(83.9%-100%)	47.5% (37.5%-57.7%)
Group4 50-59 years	.655 (.598-.712)	37% (27.9%-46.9%)	93.9%(83.1%-98.7%)	93% (80.9%-98.5%)	40.4%(31.3%-49.9%)
Group4 >60 years	.649(.567-.732)	35% (20.6%-51.7%)	94.9%(82.7%-99.4%)	87.5%(61.7%-98.4%)	58.7%(45.6%-71%)
Cut off	1.4				
Whole cohort	.63 (.602-.658)	28.4%(23.2%-33.9%)	97.6%(94.5%-99.2%)	94.3%(87.1%-98.1%)	49.6%(44.7%-54.6%)
Group1 < 30 years	.591 (.508-.673)	18.2%(5.19%-40.3%)	100% (89.1%-100%)	100% (39.8%-100%)	64% (49.2%-77.1%)
Group2 30-39 years	.589(.532-.645)	17.8%(8% -32.1%)	100% (91.4%-100%)	100%(63.1%-100%)	52.6% (40.9%-64%)
Group3 40-49 years	.635(.584-.686)	27% (17.4%-38.6%)	100%(92.6%-100%)	100% (83.2%-100%)	47.1% (37.1%-57.2%)

Group4 50-59 years	.645 (.589-.702)	35.2%(26.2%-45%)	93.9%(83.1%-98.7%)	92.7%(80.1%-98.5%)	39.7%(30.7%-49.2%)
Group4 >60 years	.624 (.544-.704)	30% (16.6%-46.5%)	94.9%(82.7%-99.4%)	85.7%(57.2%-98.2%)	56.9%(44%-69.2%)
Cut off	1.5				
Whole cohort	.621(.594-.647)	25.6%(20.7%-31%)	98.6%(95.9%-99.7%)	96.1%(89%-99.2%)	48.9%(44.1%-53.8%)
Group1 < 30 years	.568(.495-.642)	13.6%(2.91%-34.9%)	100% (89.1%-100%)	100%(29.2%-100%)	62.7%(48.1%-75.9%)
Group2 30-39 years	.589 (.532-.645)	17.8%(8%-32.1%)	100%(91.4%-100%)	100%(63.1%-100%)	52.6% (40.9%-64%)
Group3 40-49 years	.622 (.572-.671)	24.3%(15.1%-35.7%)	100% (92.6%-100%)	100%(81.5%-100%)	46.2%(36.3%-56.2%)
Group4 50-59 years	.646 (.594-.699)	33.3%(24.6%-43.1%)	95.9%(86%-99.5%)	94.7%(82.3%-99.4%)	39.5% (30.7%-48.9%)
Group4 >60 years	.6 (.529- .67)	22.5% (10.8%-38.5%)	97.4%(86.5%-99.9%)	90% (55.5%-99.7%)	55.1%(42.6%-67.1%)

********************************CSI********************************

Diagnostic accuracy of combined sensory index CSI, All cutoff values , all age groups

Age group	ROC	Sensitivity	Specificity	PPV	NPV
Cut off	1.0				
Whole cohort	.763(.724-.801)	83% (78.4% -87%)	69.5%(62.6%-75.8%)	80.9%(76.2%-85.1%)	72.4% (65.5%-78.6%)
Group1 < 30 years	.763 (.649-.877)	59.1% (36.4%-79.3%)	93.5%(78.6%-99.2%)	86.7%(59.5%-98.3%)	76.3%(59.8%-88.6%)
Group2 30-39 years	.722 (.627-.816)	66% (51.2%-78.8%)	78.4%(61.8%-90.2%)	80.5%(65.1%-91.2%)	63%(47.5%-76.8%)
Group3 40-49 years	.804 (.731-.877)	82.1%(71.7%-89.8%)	78.7%(64.3%-89.3%)	86.5%(76.5%-93.3%)	72.5%(58.3%-84.1%)
Group4 50-59 years	.737 (.659-.815)	88.7%(81.4%-93.8%)	58.7%(43.2%-73%)	84.3%(76.6%-90.3%)	67.5%(50.9%-81.4%)
Group4 >60 years	.718 (.639-.797)	100%(92.5%-100%)	43.6% (27.8%-60.4%)	68.1% (55.8%-78.8%)	100%(80.5%-100%)
Cut off	1.1				
Whole cohort	.775 (.737-.812)	81.4% (76.6%-85.6%)	73.5%(66.8%-79.5%)	82.7%(78%-86.8%)	71.7%(65%-77.8%)
Group1 < 30 years	84.6% (54.6%-98.1%)	50% (28.2%-71.8%)	93.5%(78.6%-99.2%)	84.6%(54.6%-98.1%)	72.5% (56.1%-85.4%)
Group2 30-39 years	.749(.659-.838)	66% (51.2%-78.8%)	83.8%(68%-93.8%)	84.6%(69.5%-94.1%)	64.6%(49.5%-77.8%)
Group3 40-49 years	.808 (.736-.88)	80.8% (70.3%-88.8%)	80.9% (66.7%-90.9%)	87.5% (77.6%-94.1%)	71.7%(57.7%-83.2%)
Group4 50-59 years	.761 (.685-.837)	87% (79.4%-92.5%)	65.2%(49.8%-78.6%)	86.2% (78.6%-91.9%)	66.7%(51%-80%)
Group4 >60 years	.744 (.664-.823)	100%(92.5%-100%)	48.7%(32.4%-65.2%)	70.1%(57.7%-80.7%)	100% (82.4%-100%)
Cut off	1.2				
Whole cohort	.781(.744-.818)	79.2%(74.2%-83.5%)	77% (70.5%-82.6%)	84.3% (79.6%-88.3%)	70.3% (63.8%-76.3%)
Group1 < 30 years	.734 (.622-.845)	50% (28.2%-71.8%)	96.8%(83.3%-99.9%)	91.7%(61.5%-99.8%)	73.2%(57.1%-85.8%)
Group2 30-39 years	.766(.682-.85)	64% (49.2%-77.1%)	89.2%(74.6%-97%)	88.9%(73.9%-96.9%)	64.7%(50.1%-77.6%)
Group3 40-49 years	.8 (.728-.871)	76.9%(66%-85.7%)	83% (69.2%-92.4%)	88.2%(78.1%-94.8%)	68.4%(54.8%-80.1%)

Group4 50-59 years	.778 (.704-.853)	86.1%(78.4%-91.8%)	69.6%(54.2%-82.3%)	87.6% (80.1%-93.1%)	66.7%(51.6%-79.6%)
Group4 >60 years	.735 (.65-.82)	95.7%(85.5%-99.5%)	51.3% (34.8%-67.6%)	70.3%(57.6%-81.1%)	90.9% (70.8%-98.9%)
Cut off	1.3				
Whole cohort	.789(.753-.826)	77.9% (72.9%-82.4%)	80% (73.8%-85.3%)	85.9%(81.3%-89.7%)	69.9%(63.5%-75.7%)
Group1 < 30 years	.734 (.622-.845)	50% (28.2%-71.8%)	96.8%(83.3%-99.9%)	91.7%(61.5% 99.8%)	73.2% (57.1%-85.8%)
Group2 30-39 years	.759 (.678-.841)	60% (45.2%-73.6%)	91.9% (78.1%-98.3%)	90.9% (75.7%-98.1%)	63% (48.7%-75.7%)
Group3 40-49 years	.821(.753-.888)	76.9%(66%-85.7%)	87.2%(74.3%-95.2%)	90.9%(81.3%-96.6%)	69.5%(56.1%-80.8%)
Group4 50-59 years	.78 (.707 .854)	84.3%(76.4%-90.5%)	71.7% (56.5%-84%)	88.2% (80.6%-93.6%)	64.7% (50.1%-77.6%)
Group4 >60 years	.761 (.677-.845)	95.7%(85.5%-99.5%)	56.4% (39.6%-72.2%)	72.6%(59.8%-83.1%)	91.7% (73%-99%)
Cut off	1.4				
Whole cohort	.779 (.743-.816)	74.4%(69.1%-79.1%)	81.5% (75.4%-86.6%)	86.2% (81.5%-90.1%)	67.1%(60.8%-73%)
Group1 < 30 years	.727 (.621-.834)	45.5%(24.4%-67.8%)	100% (88.8%-100%)	100% (69.2%-100%)	72.1%(56.3%-84.7%)
Group2 30-39 years	.739 (.657-.822)	56% (41.3% -70%)	91.9%(78.1%-98.3%)	90.3%(74.2%-98%)	60.7%(46.8%-73.5%)
Group3 40-49 years	.812 (.746-.879)	73.1%(61.8%-82.5%)	89.4% (76.9%-96.5%)	91.9%(82.2%-97.3%)	66.7% (53.7%-78%)
Group4 50-59 years	.774(.7 -.848)	80.9% (72.5%-87.6%)	73.9% (58.9%-85.7%)	88.6%(80.9%-94%)	60.7% (46.8%-73.5%)
Group4 >60 years	.75 (.664-.837)	93.6%(82.5%-98.7%)	56.4%(39.6%-72.2%)	72.1% (59.2%-82.9%)	88% (68.8%-97.5%)
Cut off	1.5				
Whole cohort	.772(.736-.808)	70.8%(65.4%-75.8%)	83.5% (77.6%-88.4%)	87% (82.2%-90.9%)	64.7%(58.6%-70.6%)
Group1 < 30 years	100%(63.1%-100%)	36.4%(17.2%-59.3%)	100% (88.8%-100%)	100%(63.1%-100%)	68.9%(53.4%-81.8%)
Group2 30-39 years	.746(.672-.821)	52% (37.4%-66.3%)	97.3%(85.8%-99.9%)	96.3%(81%-99.9%)	60% (46.5%-72.4%)
Group3 40-49 years	.799 (.732-.867)	70.5%(59.1%-80.3%)	89.4% (76.9%-96.5%)	91.7%(81.6%-97.2%)	64.6% (51.8%-76.1%)
Group4 50-59 years	.763 (.69-.837)	76.5%(67.7%-83.9%)	76.1% (61.2%-87.4%)	88.9% (81%-94.3%)	56.5% (43.3%-69%)
Group4 >60 years	.763 (.677-.849)	93.6%(82.5%-98.7%)	59% (42.1%-74.4%)	73.3%(60.3%-83.9%)	88.5% (69.8%-97.6%)

Cut off	1.6				
Whole cohort	.759 (.722-.795)	68.3%(62.8%-73.4%)	83.5%(77.6%-88.4%)	86.6%(81.7%-90.6%)	62.8%(56.7%-68.6%)
Group1 < 30 years	.659 (.559-.759)	31.8% (13.9%-54.9%)	100% (88.8%=100%)	100% (59%-100%)	67.4%(52%-80.5%)
Group2 30-39 years	.736 (.662-.811)	50% (35.5%-64.5%)	97.3%(85.8%-99.9%)	96.2%(80.4%-99.9%)	59% (45.7%-71.4%)
Group3 40-49 years	.774 (.704-.843)	65.4% (53.8%-75.8%)	89.4%(76.9%-96.5%)	91.1% (80.4%-97%)	60.9%(48.4%-72.4%)
Group4 50-59 years	.759(.685-.832)	75.7%(66.8%-83.2%)	76.1% (61.2%-87.4%)	88.8%(80.8%-94.3%)	55.6% (42.5%-68.1%)
Group4 >60 years	.752 (.664-.84)	91.5%(79.6%-97.6%)	59% (42.1%-74.4%)	72.9%(59.7%-83.6%)	85.2%(66.3%-95.8%)
Cut off	1.7				
Whole cohort	.762 (.726-.797)	66.3%(60.8%-71.6%)	86%(80.4%-90.5%)	88.1%(83.2%-91.9%)	62.1%(56.1%-67.8%)
Group1 < 30 years	.614 (.524-.703)	22.7%(7.82%-45.4%)	100%(88.8%-100%)	100% (47.8%-100%)	64.6%(49.5%-77.8%)
Group2 30-39 years	.736 (.662-.811)	50% (35.5%-64.5%)	97.3%(85.8%-99.9%)	96.2%(80.4%-99.9%)	59% (45.7%-71.4%)
Group3 40-49 years	.784 (.718-.851)	65.4%(53.8%-75.8%)	91.5%(79.6%-97.6%)	92.7%(82.4%-98%)	61.4%(49%-72.8%)
Group4 50-59 years	.752 (.679-.825)	72.2% (63%-80.1%)	78.3%(63.6%-89.1%)	89.2%(81.1%-94.7%)	52.9%(40.4%-65.2%)
Group4 >60 years	.791 (.706-.876)	91.5% (79.6%-97.6%)	66.7%(49.8%-80.9%)	76.8% (63.6%-87%)	86.7%(69.3%-96.2%)
Cut off	2.0				
Whole cohort	.749 (.714-.783)	60.3%(54.6%-65.7%)	89.5%(84.4%-93.4%)	90% (85.1%-93.7%)	59.1%(53.3%-64.7%)
Group1 < 30 years	.614 (.524-.703)	22.7% (7.82%-45.4%)	100%(88.8%-100%)	100%(47.8%-100%)	64.6%(49.5%-77.8%)
Group2 30-39 years	.716(.642-.791)	46% (31.8%-60.7%)	97.3%(85.8%-99.9%)	95.8%(78.9%-99.9%)	57.1%(44%-69.5%)
Group3 40-49 years	.746 (.678-.814)	57.7% (46%-68.8%)	91.5%(79.6%-97.6%)	91.8%(80.4%-97.7%)	56.6%(44.7%-67.9%)
Group4 50-59 years	.754 (.686-.822)	66.1% (56.7%-74.7%)	84.8% (71.1%-93.7%)	91.6%(83.4%-96.5%)	50% (38.5%-61.5%)
Group4 >60 years	.8 (.713-.886)	83% (69.2%-92.4%)	76.9% (60.7%-88.9%)	81.3%(67.4%-91.1%)	78.9%(62.7%-90.4%)
Cut off	2.5				
Whole cohort	.709 (.676-.742)	48.7% (43%-54.4%)	93% (88.5%-96.1%)	91.6%(86.3%-95.3%)	53.8%(48.3%-59.1%)
Group1 < 30 years	.614 (.524-.703)	22.7% (7.82%-45.4%)	100% (88.8%-100%)	100%(47.8%-100%)	64.6%(49.5%-77.8%)

Group2 30-39 years	.676 (.604-.749)	38% (24.7%-52.8%)	97.3% (85.8%-99.9%)	95% (75.1%-99.9%)	53.7%(41.1%-66%)
Group3 40-49 years	.716(.653-.779)	47.4%(36%-59.1%)	95.7%(85.5%-99.5%)	94.9%(82.7%-99.4%)	52.3%(41.3%-63.2%)
Group4 50-59 years	.717 (.656-.779)	52.2%(42.7%-61.6%)	91.3%(79.2%-97.6%)	93.8% (84.8%-98.3%)	43.3%(33.3%-53.7%)
Group4 >60 years	.74(.648-.832)	66% (50.7%-79.1%)	82.1%(66.5%-92.5%)	81.6%(65.7%-92.3%)	66.7%(51.6%-79.6%)
Cut off	3.0				
Whole cohort	.688 (.657-.718)	41% (35.5%-46.7%)	96.5%(92.9%-98.6%)	94.8%(89.6%-97.9%)	51.2%(46%-56.3%)
Group1 < 30 years	.591 (.508-.673)	18.2% (5.19%-40.3%)	100%(88.8%-100%(	100% (39.8%-100%)	63.3%(48.3%-76.6%)
Group2 30-39 years	.65 (.586-.714)	30% (17.9%-44.6%)	100% (90.5%-100%)	100% (78.2%-100%)	51.4%(39.3%-63.3%)
Group3 40-49 years	.692(.638-.747)	38.5%(27.7%-50.2%)	100%(92.5%-100%)	100% (88.4%-100%)	49.5%(39.1%-59.9%)
Group4 50-59 years	.707 (.656-.757)	43.5%(34.3%-53%)	97.8%(88.5%-99.9%)	98% (89.6%-100%)	40.9%(31.6%-50.7%)
Group4 >60 years	.732 (.641-.822)	61.7%(46.4%-75.5%)	84.6%(69.5%-94.1%)	82.9% (66.4%-93.4%)	64.7%(50.1%-77.6%)
Cut off	3.5				
Whole cohort	.655 (.627-.683)	33% (27.8%-38.5%)	98% (95%-99.5%)	96.3%(90.7%-99%)	48.4%(43.4%-53.4%)
Group1 < 30 years	.568 (.495-.642)	13.6%(2.91%-34.9%)	100% (88.8%-100%)	100% (29.2%-100%)	62%(47.2%-75.3%)
Group2 30-39 years	.61 (.552-.668)	22% (11.5% -36%)	100% (90.5%- 100%)	100% (71.5%- 100%)	48.7% (37%- 60.4%)
Group3 40-49 years	.667 (.614-.719)	33.3% (23.1%- 44.9%)	100% (92.5%-100%)	100% (86.8%-100%)	47.5% (37.3%-57.8%)
Group4 50-59 years	.672 (.623-.721)	36.5% (27.7%-46%)	97.8% (88.5%-99.9%)	97.7% (87.7%-99.9%)	38.1% (29.4%-47.5%)
Group4 >60 years	.685 (.602- .768)	44.7% (30.2%-59.9%)	92.3% (79.1%- 98.4%)	87.5% (67.6%- 97.3%)	58.1% (44.8%-70.5%)
