# Supplementary material for: Values and Diagnostic Accuracy of Electrodiagnostic Findings in Carpal Tunnel Syndrome Based on Age, Gender, and Diabetes
Source: Diagnostics (Basel). 2024 Jun 28;14(13):1381. doi: 10.3390/diagnostics14131381 (PMC11240809; doi:10.3390/diagnostics14131381)
Supplement: Supplementary file 1 [file diagnostics-14-01381-s001.zip › Table S5 Diagnostic accuracy of median latency at Digit II and comparative latency studies (COLSs), all cutoff values (participants with no DM).pdf]

Table S5 Diagnostic accuracy of median latency at Digit II, All cutoff values , (no DM)

Age group	ROC	Sensitivity	Specificity	PPV	NPV
Cut off	3.5 (ms)				
Whole cohort	.745(.706-.784)	65.3%(59.4%-71%)	83.7%(77.4%-88.8%)	86.1% (80.6%-90.5%)	61.1%(54.6%-67.2%)
Group1 < 30 years	.597 (.487-.708)	28.6%(11.3%-52.2%)	90.9%(75.7%-98.1%)	66.7%(29.9%-92.5%)	66.7% (51%-80%)
Group2 30-39 years	.701(.614-.788)	52.1%(37.2%-66.7%)	88.1%(74.4%-96%)	83.3%(65.3%-94.4%)	61.7%(48.2%-73.9%)
Group3 40-49 years	.762 (.69-.834)	64.4%(52.3%-75.3%)	88% (75.7%-95.5%)0	88.7%(77%-95.7%)	62.9% (50.5%-74.1%)
Group4 50-59 years	.742 (.657-.826)	71.8% (62.1%-80.3%)	76.5% (58.8%-89.3%)	90.2%(81.7%-95.7%)	47.3%(33.7%-61.2%)
Group4 >60 years	.781(.66-.902)	93.1%(77.2%-99.2%)	63.2%(38.4%-83.7%)	79.4%(62.1%-91.3%)	85.7% (57.2%-98.2%)
Cut off	3.6 (ms)				
Whole cohort	88.4% (82.7%-92.8%)	55.8% (49.7%-61.8%)	88.8% (83.2%-93%)	88.4%(82.7%-92.8%)	56.6% (50.6%-62.5%)
Group1 < 30 years	.565 (.469-.66)	19% (5.45%-41.9%)	93.9%(79.8%-99.3%)	66.7%(22.3%-95.7%)	64.6%(49.5%-77.8%)
Group2 30-39 years	.714 (.633-.796)	50% (35.2%-64.8%)	92.9%(80.5%-98.5%)	88.9%(70.8%-97.6%)	61.9% (48.8%-73.9%)
Group3 40-49 years	.724 (.653-.795)	54.8%(42.7%-66.5%)	90% (78.2%-96.7%)	88.9% (75.9%-96.3%)	57.7%(46%-68.8%)
Group4 50-59 years	.723 (.646-.8)	59.2% (49.1%-68.8%)	85.3%(68.9%-95%)	92.4% (83.2%-97.5%)	40.8%(29.3%-53.2%)
Group4 >60 years	.782(.659-.906)	82.8%(64.2%-94.2%)	73.7% (48.8%-90.9%)	82.8%(64.2%-94.2%)	73.7% (48.8%-90.9%)
Cut off	3.7				
Whole cohort	.727 (.692-.762)	52.2%(46.1%-58.2%)	93.3% (88.5%-96.5%)	92.3% (86.9%-95.9%)	55.9%(50%-61.6%)
Group1 < 30 years	.565(.469-.66)	19% (5.45%-41.9%)	93.9% (79.8%-99.3%)	66.7%(22.3%-95.7%)	64.6%(49.5%-77.8%)
Group2 30-39 years	.705 (.627-.784)	45.8%(31.4%-60.8%)	95.2%(83.8%-99.4%)	91.7%(73%-99%)	60.6%(47.8%-72.4%)
Group3 40-49 years	.683 (.612-.754)	46.6% (34.8%-58.6%)	90% (78.2%-96.7%)	87.2% (72.6%-95.7%)	53.6% (42.4%-64.5%)
Group4 50-59 years	.777(.721-.832)	58.3%(48.1%-67.9%)	97.1% (84.7%-99.9%)	98.4% (91.2%-100%)	43.4% (32.1%-55.3%)
Group4 >60 years	.844 (.741-.947)	79.3% (60.3%-92%)	89.5% (66.9%-98.7%)	92% (74% -99%)	73.9%(51.6%-89.8%)

Cut off	3.9				
Whole cohort	.709 (.675-.743)	47.4%(41.4%-53.5%)	94.4%(89.9%-97.3%)	92.9%(87.3%-96.5%)	53.8% (48.1%-59.5%)
Group1 < 30 years	.58 (.489-.671)	19% 5.45% 41.9%	97% 84.2% 99.9%	80% 28.4% 99.5%	65.3% 50.4% 78.3%
Group2 30-39 years	.685 .607 .762	41.7%(27.6%-56.8%)	95.2%(83.8%-99.4%)	90.9%(70.8%-98.9%)	58.8%(46.2%-70.6%)
Group3 40-49 years	.672 (.604-.741)	42.5%(31%-54.6%)	92% (80.8%-97.8%)	88.6% (73.3%-96.8%)	52.3%(41.4%-63%)
Group4 50-59 years	.752 (.696-.809)	53.4%(43.3%-63.3%)	97.1%(84.7%-99.9%)	98.2%(90.4%-100%)	40.7% (29.9%-52.2%)
Group4 >60 years	.792 (.681-.903)	69% (49.2%-84.7%)	89.5%(66.9%-98.7%)	90.9%(70.8%-98.9%)	65.4%(44.3%-82.8%)
Cut off	4.0				
Whole cohort	.702 (.671-.734)	42.7% (36.8%-48.8%)	97.8%(94.3%-99.4%)	96.7%(91.8%-99.1%)	52.6% (47%-58.1%)
Group1 < 30 years	.58 (.489-.671)	19% (5.45%-41.9%)	97% (84.2%-99.9%)	80% (28.4%-99.5%)	65.3%(50.4%-78.3%)
Group2 30-39 years	.655 (.583-.726)	33.3% (20.4%-48.4%)	97.6%(87.4%-99.9%)	94.1%(71.3%-99.9%)	56.2% (44.1%-67.8%)
Group3 40-49 years	.675 (.616-.734)	37% (26% -49.1%)	98% (89.4%-99.9%)	96.4% (81.7%-99.9%)	51.6% (41.1%-62%)
Group4 50-59 years	.733 (.676-.789)	49.5%(39.5%-59.5%)	97.1% (84.7%-99.9%)	98.1%(89.7%-100%)	38.8%(28.4%-50%)
Group4 >60 years	.828 (.74-.916)	65.5%(45.7%-82.1%)	100% (82.4%-100%)	100%(82.4%-100%)	65.5%(45.7%-82.1%)
Cut off	4.1				
Whole cohort	38.3%(32.5%-44.4%)	38.3% (32.5%-44.4%)	98.9%(96%-99.9%)	98.1%(93.4%-99.8%)	51% (45.6%-56.4%)
Group1 < 30 years	.548 (.483-.612)	9.52% (1.17%-30.4%)	100%(89.4%-100%)	100% (15.8%-100%)	63.5%(49%-76.4%)
Group2 30-39 years	.644 (.574-.715)	31.3% (18.7%-46.3%)	97.6%(87.4%-99.9%)	93.8%(69.8%-99.8%)	55.4%(43.4%-67%)
Group3 40-49 years	.641 (.584-.697)	30.1%(19.9%-42%)	98% (89.4%-99.9%)	95.7%(78.1%-99.9%)	49% (38.9%-59.2%)
Group4 50-59 years	.733 (.685-.781)	46.6% (36.7%-56.7%)	100% (89.7%-100%)	100%(92.6%-100%)	38.2% (28.1%-49.1%)
Group4 >60 years	.81 (.72 -.9)	62.1% (42.3%-79.3%)	100% (82.4%-100%)	100% (81.5%-100%)	63.3%(43.9%-80.1%)
Cut off	4.2				
Whole cohort	.681 (.651-.71)	37.2% (31.5%-43.2%)	98.9%(96%-99.9%)	98.1% (93.2%-99.8%)	50.6% (45.2%-55.9%)
Group1 < 30 years	.548(.483-.612)	9.52%(1.17%-30.4%)	100% (89.4%-100%)	100% (15.8%-100%)	63.5%(49%-76.4%)

Group2 30-39 years	.644 (.574-.715)	31.3%(18.7%-46.3%)	97.6%(87.4%-99.9%)	93.8% (69.8%-99.8%)	55.4%(43.4%-67%)
Group3 40-49 years	.641 (.584-.697)	30.1%(19.9%-42%)	98% (89.4%-99.9%)	95.7%(78.1%-99.9%)	49% (38.9%-59.2%)
Group4 50-59 years	.723 (.675-.772)	44.7% (34.9%-54.8%)	100% (89.7%-100%)	100% (92.3%-100%)	37.4%(27.4%-48.1%)
Group4 >60 years	.793 (.702-.884)	58.6%(38.9%-76.5%)	100%(82.4%-100%)	100% 80.5% 100%	61.3% 42.2% 78.2%
Cut off	4.3				
Whole cohort	.669 (.64 -.697)	34.3%(28.7%-40.3%)	34.3%(28.7%-40.3%)	98.9%(94.3%-100%)	49.6%(44.3%-54.9%)
Group1 < 30 years	.548 (.483-.612)	9.52%(1.17%-30.4%)	100%(89.4%-100%)	100% (15.8%-100%)	63.5%(49%-76.4%)
Group2 30-39 years	.646 (.581-.711)	29.2%(17%-44.1%)	100%(91.6%-100%)	100% (76.8%-100%)	55.3%(43.4%-66.7%)
Group3 40-49 years	.627 (.572-.682)	27.4%(17.6%-39.1%)	98%(89.4%-99.9%)	95.2% (76.2%-99.9%)	48%(38%-58.2%)
Group4 50-59 years	.699 (.652-.747)	39.8%(30.3%-49.9%)	100% (89.7%-100%)	100% (91.4%-100%)	35.4%(25.9%-45.8%)
Group4 >60 years	.793 (.702-.884)	58.6%(38.9%-76.5%)	100% (82.4%-100%)	100% (80.5%-100%)	61.3%(42.2%-78.2%)
Cut off	4.4				
Whole cohort	.654 (.626-.682)	31.4% (25.9%-37.2%)	99.4%(96.9%-100%)	98.9% (93.8%-100%)	48.5%(43.3%-53.8%)
Group1 < 30 years	.524 (.477-.57)	4.76%(.12%-23.8%)	100%(89.4%-100%)	100% (2.5%-100%)	62.3% (47.9%-75.2%)
Group2 30-39 years	.635 (.572-.699)	27.1% (15.3%-41.8%)	100% (91.6%-100%)	100% (75.3%-100%)	54.5% (42.8%-65.9%)
Group3 40-49 years	.606 (.554-.659)	23.3%(14.2%-34.6%)	98% (89.4%-99.9%)	94.4%(72.7%-99.9%)	46.7% (36.9%-56.7%)
Group4 50-59 years	.689 (.642-.736)	37.9% (28.5%-48%)	100%(89.7%-100%)	100% (91%-100%)	34.7%(25.4%-45%)
Group4 >60 years	.776(.684-.868)	55.2% (35.7%-73.6%)	100%(82.4%-100%)	100%(79.4%-100%)	59.4% (40.6%-76.3%)
Cut off	4.5				
Whole cohort	.638 (.61-.665)	28.1% (22.9%-33.8%)	99.4%(96.9%-100%)	98.7%(93.1%-100%)	47.3%(42.2%-52.5%)
Group1 < 30 years					
Group2 30-39 years	.625 (.563-.687)	25% (13.6%-39.6%)	100% (91.6%-100%)	100%(73.5%-100%)	53.8% (42.2%-65.2%)
Group3 40-49 years	.606 (.554-.659)	23.3%(14.2%-34.6%)	98% (89.4%-99.9%)	94.4%(72.7%-99.9%)	46.7% (36.9%-56.7%)

Group4 50-59 years	.67(.624-.716)	34% (24.9%- 44%)	100%(89.7%-100%)	100% (90%-100%)	33.3%(24.3%-43.4%)
Group4 >60 years	.724 (.632-.816)	44.8%(26.4%-64.3%)	100%(82.4%-100%)	100% (75.3%-100%)	54.3%(36.6%-71.2%)

**********************************Palmdiff**********************************

Diagnostic accuracy of palmdiff, All cutoff values , (no DM).

Age group	ROC	Sensitivity	Specificity	PPV	NPV
Cut off	0.4				
Whole cohort	0.794(0.756- 0.831)	70.7% (64.6%- 76.3%)	88% (82.1%- 92.5%)	89.7% (84.5%- 93.6%)	67.1%(60.5%- 73.3%)
Group1 < 30 years	0.61 (0.508- 0.712)	25% (8.66% - 49.1%)	97% (84.2%- 99.9%)	83.3%(35.9%- 99.6%)	68.1% (52.9%- 80.9%)
Group2 30-39 years	0.749 (0.662- 0.836)	60% (44.3%- 74.3%)	89.7% (75.8%- 97.1%)	87.1% (70.2%- 96.4%)	66% (51.7%- 78.5%)
Group3 40-49 years	0.775 (0.699- 0.851)	68.7% (56.2%- 79.4%)	86.4% (72.6%- 94.8%)	88.5% (76.6%- 95.6%)	64.4% (50.9%- 76.4%)
Group4 50-59 years	0.811 (0.731- 0.891)	82.8% (73.2%- 90%)	79.4% (62.1%- 91.3%)	91.1% (82.6%- 96.4%)	64.3%(48%- 78.4%)
Group4 >60 years	0.886 (0.786- 0.985)	88.9%(70.8%- 97.6%)	88.2% (63.6%- 98.5%)	92.3% (74.9%- 99.1%)	83.3% (58.6%- 96.4%)
Cut off	0.5				
Whole cohort	0.768 (0.733- 0.803)	58.9% (52.5%- 65.2%)	94.6% (90%- 97.5%)	94.2% (89.2%- 97.3%)	61% (54.8%- 67%)
Group1 < 30 years	0.6 (0.51- 0.69)	20% (5.73%- 43.7%)	100% (89.4%- 100%)	100% (39.8%- 100%)	67.3% (52.5%- 80.1%)
Group2 30-39 years	0.732 (0.654-0.81)	48.9% (33.7%- 64.2%)	97.4% (86.5%- 99.9%)	95.7% (78.1%- 99.9%)	62.3% (49%- 74.4%)
Group3 40-49 years	0.753 (0.68- 0.826)	59.7% (47%- 71.5%)	90.9% (78.3%- 97.5%)	90.9% (78.3%- 97.5%)	59.7% (47%- 71.5%)
Group4 50-59 years	0.778 (0.708- 0.848)	64.4% (53.4%- 74.4%)	91.2% (76.3%- 98.1%)	94.9% (85.9%- 98.9%)	50% (37% -63%)
Group4 >60 years	0.897 (0.807- 0.986)	85.2% (66.3%- 95.8%)	94.1% (71.3%- 99.9%)	95.8% (78.9%- 99.9%)	80% (56.3%- 94.3%)
Cut off	0.6				
Whole cohort	755 (0.722- 0.789)	54.1% (47.6%- 60.4%)	97% (93.2%- 99%)	96.4% (91.7%- 98.8%)	58.9% (52.8%- 64.8%)
Group1 < 30 years	0.575(0.495 -0.655)	15% (3.21%- 37.9%)	100% (89.4%- 100%)	100% (29.2%- 100%)	66% (51.2%- 78.8%)

Group2 30-39 years	0.709 (0.632-0.787)	44.4% (29.6%-60%)	97.4%(86.5%-99.9%)	95.2%(76.2%-99.9%)	60.3% (47.2%-72.4%)
Group3 40-49 years	0.742 (0.671 -0.813)	55.2%(42.6%-67.4%)	93.2% (81.3%-98.6%)	92.5% (79.6%-98.4%)	57.7% (45.4%-69.4%)
Group4 50-59 years	0.778(0.719-0.838)	58.6% (47.6%-69.1%)	97.1% (84.7%-99.9%)	98.1% (89.7%-100%)	47.8% (35.6%-60.2%)
Group4 >60 years	0.907 (0.833 -0.982)	81.5%(61.9% -93.7%)	100%(80.5%-100%)	100%(84.6%-100%)	77.3% (54.6%-92.2%)
Cut off	0.7				
Whole cohort	0.72(0.686-0.753)	46.3% (40%-52.8%)	97.6%(94% -99.3%)	96.6% (91.5%-99.1%)	55.3% (49.4%-61%)
Group1 < 30 years	0.575(0.495-0.655)	15% (3.21%-37.9%)	100% (89.4%-100%)	100% (29.2%-100%)	66% (51.2%-78.8%)
Group2 30-39 years	0.665(0.59-0.74)	35.6%(21.9%-51.2%)	97.4% (86.5%-99.9%)	94.1% (71.3%-99.9%)	56.7%(44%-68.8%)
Group3 40-49 years	0.724 (0.656-0.791)	49.3% (36.8%-61.8%)	95.5% (84.5%-99.4%)	94.3% (80.8%-99.3%)	55.3%(43.4%-66.7%)
Group4 50-59 years	0.727(0.667-0.787)	48.3%(37.4%-59.2%)	97.1%(84.7%-99.9%)	97.7% (87.7% 99.9%	42.3% (31.2%-54%)
Group4 >60 years	0.87 (0.786-0.955)	74.1% (53.7%-88.9%)	100% (80.5%-100%)	100%(83.2%-100%)	70.8% (48.9%-87.4)
Cut off	0.8				
Whole cohort	0.697(0.665-0.729)	40.7% (34.5%-47.1%)	98.8%(95.7%-99.9%)	98% (93.1%-99.8%)	53.1% (47.3%-58.7%)
Group1 < 30 years	0.575 (0.495-0.655)	15% (3.21%-37.9%)	100% (89.4%-100%)	100% (29.2%-100%)	66% (51.2%-78.8%)
Group2 30-39 years	0.678 (0.607-0.748)	35.6% (21.9%-51.2%)	100%(91%-100%)	100% (79.4%-100%)	57.4% (44.8%-69.3%)
Group3 40-49 years	0.709 (0.641-0.776)	46.3% (34% -58.9%)	95.5% (84.5%-99.4%)	93.9% (79.8%-99.3%)	53.8% (42.2%-65.2%)
Group4 50-59 years	0.701 (0.649-0.753)	40.2% (29.9%-51.3%)	100% (89.7%-100%)	100% (90%-100%)	39.5% (29.2%-50.7%)
Group4 >60 years	0.778 (0.682-0.873)	55.6% (35.3%-74.5%)	100%(80.5%-100%)	100% (78.2%-100%)	58.6% (38.9%-76.5%)

Cut off	0.9				
Whole cohort	.675 (.644-.706)	36.2% (30.2%-42.5%)	98.8% (95.7%-99.9%)	97.8% (92.3%-99.7%)	51.2% (45.6%-56.8%)
Group1 < 30 years	0.575 (0.495-0.655)	15% (3.21%-37.9%)	100% (89.4%-100%)	100% (29.2%-100%)	66% (51.2%-78.8%)
Group2 30-39 years	0.656(0.587-0.724)	31.1% (18.2%-46.6%)	100% (91%-100%)	100%(76.8%-100%)	55.7%(43.3%-67.6%)
Group3 40-49 years	0.679 (0.612-0.746)	40.3% (28.5%-53%)	95.5% (84.5%-99.4%)	93.1% (77.2%-99.2%)	51.2%(39.9%-62.4%)
Group4 50-59 years	0.684(0.633-0.735)	36.8% (26.7%-47.8%)	100% (89.7%-100%)	100%(89.1%-100%)	38.2% (28.1%-49.1%)
Group4 >60 years	0.741 (0.645-0.837)	48.1% (28.7%-68.1%)	100%(80.5%-100%)	100% (75.3%-100%)	54.8%(36%-72.7%)
Cut off	1.0				
Whole cohort	0.654 (0.624-0.683)	31.3% (25.6%-37.5%)	99.4% (96.7%-100%)	98.7% (93.1%-100%)	49.6% (44.1%-55%)
Group1 < 30 years	575(0.495-0.655)	15% (3.21%-37.9%)	100%(89.4%-100%)	100% (29.2%-100%)	66% (51.2%-78.8%)
Group2 30-39 years	0.644 (0.577-0.711)	28.9% (16.4%-44.3%)	100%(91%-100%)	100% (75.3%-100%)	54.9% (42.7%-66.8%)
Group3 40-49 years	0.645(0.585-0.706)	31.3% (20.6%-43.8%)	97.7%(88%-99.9%)	95.5% (77.2%-99.9%)	48.3% (37.6%-59.2%)
Group4 50-59 years	0.672(0.622-0.723)	34.5% (24.6% -45.4%)	100% (89.7%-100%)	100%(88.4%-100%)	37.4%(27.4%-48.1%)
Group4 >60 years	0.685 (0.592-0.778)	37% (19.4%-57.6%)	100%(80.5%-100%)	100%(69.2%-100%)	50% (32.4%-67.6%)
Cut off	1.1				
Whole cohort	0.635(0.607-0.664)	27.6% (22.2%-33.7%)	99.4% (96.7%-100%)	98.6%(92.2%-100%)	48.3% (42.9%-53.7%)
Group1 < 30 years	0.55(0.483-0.617)	10% (1.23%-31.7%)	100% (89.4%-100%)	100% (15.8%-100%)	64.7% (50.1%-77.6%)
Group2 30-39 years	0.611 (0.55 -0.673)	22.2% (11.2%-37.1%)	100% (91%-100%)	100% (69.2%-100%)	52.7%(40.7%-64.4%)

Group3 40-49 years	0.623 (0.565-0.681)	26.9% (16.8%-39.1%)	97.7%(88%-99.9%)	94.7%(74%-99.9%)	46.7% (36.3%-57.4%)
Group4 50-59 years	0.667 (0.617-0.716)	33.3% (23.6%-44.3%)	100%(89.7%-100%)	100% (88.1%-100%)	37% (27.1%-47.7%)
Group4 >60 years	0.667(0.576-0.757)	33.3% (16.5%-54%)	100% (80.5%-100%)	100%(66.4%-100%)	48.6% (31.4%-66%)
Cut off	1.2				
Whole cohort	0.617 (0.59 -0.644)	24% (18.8%-29.8%)	99.4% (96.7%-100%)	98.3% (91.1%-100%)	47% (41.7%-52.4%)
Group1 < 30 years	0.525 (0.476-0.574)	5%(.127%-24.9%)	100%(89.4%-100%)	100%(2.5%-100%)	63.5% (49%-76.4%)
Group2 30-39 years	0.611 (0.55-0.673)	22.2% (11.2%-37.1%)	100% (91%-100%)	100%(69.2%-100%)	52.7%(40.7%-64.4%)
Group3 40-49 years	0.608 (0.552-0.664)	23.9%(14.3%-35.9%)	97.7%(88%-99.9%)	94.1% (71.3%-99.9%)	45.7% (35.4%-56.3%)
Group4 50-59 years	0.638 (0.591-0.685)	27.6% (18.5%-38.2%)	100% (89.7%-100%)	100% (85.8% -100%)	35.1% (25.6%-45.4%)
Group4 >60 years	0.648(0.56-0.736)	29.6% (13.8%-50.2%)	100%(80.5%-100%)	100%(63.1%-100%)	47.2% (30.4%-64.5%)
Cut off	1.3				
Whole cohort	0.603 (0.576-0.629)	21.1% (16.2%-26.8%)	99.4% (96.7%-100%)	98.1% (89.9%-100%)	46.1%(40.9%-51.4%)
Group1 < 30 years					
Group2 30-39 years	6 (0.541-0.659)	20% (9.58%-34.6%)	100% (91%-100%)	100%(66.4%-100%)	52%(40.2%-63.7%)
Group3 40-49 years	0.586 (0.533-0.638)	19.4% (10.8%-30.9%)	97.7% (88% -99.9%)	92.9% (66.1%-99.8%)	44.3% (34.2%-54.8%)
Group4 50-59 years	0.626 (0.581-0.672)	25.3% (16.6%-35.7%)	100%(89.7%-100%)	100%(84.6%-100%)	34.3% (25.1%-44.6%)
Group4 >60 years	0.648 (0.56-0.736)	29.6% (13.8%-50.2%)	100%(80.5%-100%)	100%(63.1%-100%)	47.2% (30.4%-64.5%)
Cut off	1.4				

Whole cohort	0.591 (0.565-0.616)	18.7%(14%-24.1%)	99.4% (96.7%-100%)	97.9% (88.7%-99.9%)	45.4% (40.2%-50.6%)
Group1 < 30 years					
Group2 30-39 years	0.589 (0.532-0.645)	17.8% (8% -32.1%)	100% (91%-100%)	100%(63.1%-100%)	51.3%(39.6%-63%)
Group3 40-49 years	0.556(0.509-0.603)	13.4% (6.33%-24%)	97.7% (88%-99.9%)	90% (55.5%-99.7%)	42.6% (32.8%-52.8%)
Group4 50-59 years	0.621 (0.575-0.666)	24.1%(15.6%-34.5%)	100% (89.7%-100%)	100%(83.9%-100%)	34% (24.8%-44.2%)
Group4 >60 years	0.648(0.56-0.736)	29.6% *13.8%-50.2%)	100% (80.5%-100%)	100% (63.1%-100%)	47.2% (30.4%-64.5%)

******************************Thumbdiff********************************

Diagnostic accuracy of thumbdiff, All cutoff values , (no DM).

Age group	ROC	Sensitivity	Specificity	PPV	NPV
Cut off	0.5				
Whole cohort	.742(.699-.785)	82.1% (76.8%-86.6%)	66.3%(58.6%-73.4%)	78.3%(72.9%-83.2%)	71.3% (63.6%-78.3%)
Group1 < 30 years	.708(.576-.84)	65%(40.8%-84.6%)	76.7% (57.7%-90.1%)	65% (40.8%-84.6%)	76.7% (57.7%-90.1%)
Group2 30-39 years	.669 (.569-.77)	72.3% (57.4%-84.4%)	61.5%(44.6%-76.6%)	69.4%(54.6%-81.7%)	64.9%(47.5%-79.8%)
Group3 40-49 years	.73 (.647-.813)	79.4% (67.9%-88.3%)	66.7% (51.6%-79.6%)	77.1% (65.6%-86.3%)	69.6%(54.2%-82.3%)
Group4 50-59 years	.781(.694-.867)	88.5%(79.9%-94.3%)	67.6% (49.5%-82.6%)	87.5% (78.7%-93.6%)	69.7% (51.3%-84.4%)
Group4 >60 years	.761(.638-.883)	96.6% (82.2%-99.9%)	55.6% (30.8%-78.5%)	77.8%(60.8%-89.9%)	90.9% (58.7%-99.8%)
Cut off	0.6				
Whole cohort	.762(.721-.804)	76.1% (70.3%-81.2%)	76.3%(69.2%-82.5%)	82.7% (77.2%-87.3%)	68.3% (61.1% -74.8%)
Group1 < 30 years	.775(.655-.895)	65% (40.8%-84.6%)	90% (73.5%-97.9%)	81.3%(54.4%-96%)	79.4%(62.1%-91.3%)
Group2 30-39 years	.667(.567-.768)	61.7%(46.4%-75.5%)	71.8% (55.1%-85%)	72.5% (56.1%-85.4%)	60.9%(45.4%-74.9%)
Group3 40-49 years	.743(.661-.824)	73.5%(61.4%-83.5%)	75% (60.4%-86.4%)	80.6%)68.6%-89.6%)	66.7% (52.5%-78.9%)
Group4 50-59 years	.79 (.707-.874)	81.6% (71.9%-89.1%)	76.5% (58.8%-89.3%)	89.9%(81%-95.5%)	61.9% (45.6%-76.4%)
Group4 >60 years	.816(.699-.933)	96.6% (82.2%-99.9%)	66.7%(41%-86.7%)	82.4%(65.5%-93.2%)	92.3%(64%-99.8%)
Cut off	0.7				
Whole cohort	.756(.716-.796)	66.5%(60.3%-72.3%)	84.6%(78.3%-89.7%)	86.5% (80.9%-91%)	63% (56.4%-69.3%)
Group1 < 30 years	.658 (.546-.77)	35% (15.4%-59.2%)	96.7% (82.8%-99.9%)	87.5%(47.3%-99.7%)	69%(52.9%-82.4%)
Group2 30-39 years	.687(.593-.781)	55.3% (40.1%-69.8%)	82.1% (66.5%-92.5%)	78.8% (61.1%-91%)	60.4%(46%-73.5%)
Group3 40-49 years	.768(.695-.842)	66.2% (53.7%-77.2%)	87.5% (74.8%-95.3%)	88.2% (76.1%-95.6%)	64.6% (51.8%-76.1%)
Group4 50-59 years	.753(.669-.837)	71.3%(60.6%-80.5%)	79.4% (62.1%-91.3%)	89.9%(80.2%-95.8%)	51.9% (37.6%-66%)

Group4 >60 years	.827(.71-.943)	93.1% (77.2% - 99.2%)	72.2%(46.5%-90.3%)	84.4%(67.2%-94.7%)	86.7% (59.5%-98.3%)
Cut off	0.8				
Whole cohort	.738(.699-.777)	59.4% (53%-65.5%)	88.2% (82.3%-92.6%)	88.2% (82.3%-92.6%)	59.4%(53%-65.5%)
Group1 < 30 years	.65 (0.547-0.753)	30% (11.9%- 54.3%)	100% (88.4%- 100%)	100% (54.1%- 100%)	68.2% (52.4%-81.4%)
Group2 30-39 years	.691(.602-.781)	51.1%(36.1%-65.9%)	87.2% (72.6%-95.7%)	82.8% (64.2%-94.2%)	59.6% (45.8%-72.4%)
Group3 40-49 years	0.752 (0.682- .823)	58.8% (46.2%- 70.6%)	91.7% (80%- 97.7%)	90.9% (78.3%- 97.5%)	61.1% (48.9%-72.4%)
Group4 50-59 years	.716 (.633-.799)	60.9% (49.9%-71.2%)	82.4% (65.5%-93.2%)	89.8% (79.2%-96.2%)	45.2%(32.5%-58.3%)
Group4 >60 years	.809(.689-.93)	89.7%(72.6%-97.8%)	72.2% (46.5%-90.3%)	83.9% (66.3%-94.5%)	81.3% (54.4%-96%)
Cut off	0.9				
Whole cohort	.719 (.681-.756)	52.6%(46.2% - 58.9%)	91.1%(85.8%-94.9%0	89.8%(83.7%-94.2%)	56.4% (50.3%-62.4%)
Group1 < 30 years	.625 (.528-.722)	25% (8.66%-49.1%)	100% (88.4%-100%)	100% (47.8%-100%)	66.7%(51%-80%)
Group2 30-39 years	.696) .612-.779)	46.8% (32.1%-61.9%)	92.3% (79.1%-98.4%)	88% (68.8%-97.5%)	59% (45.7%-71.4%)
Group3 40-49 years	.723(.651-.795)	52.9% (40.4%-65.2%)	91.7%(80%-97.7%)	90% (76.3%-97.2%)	57.9% (46% -69.1%)
Group4 50-59 years	0.752 (0.682- .823)	58.8% (46.2%- 70.6%)	91.7% (80%- 97.7%)	90.9% (78.3%- 97.5%)	61.1% (48.9%-72.4%)
Group4 >60 years	.768 (.642-.895)	75.9% (56.5%-89.7%)	77.8% (52.4%-93.6%)	84.6%(65.1%-95.6%)	66.7%(43%-85.4%)
Cut off	1.0				
Whole cohort	0.706 (0.671-0.742)	46.6% (40.3%- 53%)	94.7% (90.1%- 97.5%)	92.9% (86.9%- 96.7%)	54.4% (48.5%-60.2%)
Group1 < 30 years	.6(.51- .69)	20% (5.73%-43.7%)	100%(88.4%-100%)	100% (39.8%-100%)	65.2% (49.8%-78.6%)
Group2 30-39 years	.685(.602-.768)	44.7%(30.2%-59.9%)	92.3%(79.1%-98.4%)	87.5% (67.6%-97.3%)	58.1%(44.8%-70.5%)
Group3 40-49 years	.697 (.628-.766)	45.6% (33.5%-58.1%)	93.8% (82.8%-98.7%)	91.2% (76.3%-98.1%)	54.9% (43.5%-65.9%)
Group4 50-59 years	.721(.661-.781)	47.1% (36.3%-58.1%)	97.1%(84.7%-99.9%)	97.6% (87.4%-99.9%)	41.8% (30.8%-53.4%)

Group4 >60 years	0.789 (0.676- .903)	69% (49.2%- 84.7%)	88.9% (65.3%- 98.6%)	90.9% (70.8%- 98.9%)	64% (42.5%- 82%)
Cut off	1.1				
Whole cohort	.682 (.65- .715)	39.4%(33.4%- 45.8%)	97%(93.2%- 99%)	95.2% (89.1%- 98.4%)	51.9% (46.2%- 57.5%)
Group1 < 30 years	.6 (.51- .69)	20%(5.73%- 43.7%)	100%(88.4%- 100%)	100% (39.8%- 100%)	65.2% (49.8%- 78.6%)
Group2 30-39 years	0.657 (0.584-0.73)	34% (20.9%- 49.3%)	97.4% (86.5%- 99.9%)	94.1% (71.3%- 99.9%)	55.1% (42.6%- 67.1%)
Group3 40-49 years	.66(.592- .728)	38.2% (26.7%- 50.8%)	93.8% (82.8%- 98.7%)	89.7%(72.6%- 97.8%)	51.7% (40.8%- 62.6%)
Group4 50-59 years	.707 (.655- .759)	41.4% (30.9%- 52.4%)	100% (89.7%- 100%)	100%(90.3%- 100%)	40% (29.5%- 51.2%)
Group4 >60 years	.765(.659- .872)	58.6% (38.9%- 76.5%(	94.4%(72.7%- 99.9%)	94.4%(72.7%- 99.9%)	58.6% (38.9%- 76.5%)
Cut off	1.2				
Whole cohort	.665(.634- .697)	35.5%(29.5%- 41.7%)	97.6% (94.1%- 99.4%)	95.7% (89.4%- 98.8%)	50.5% (44.9%- 56%)
Group1 < 30 years	.6 (.51 -.69)	20% (5.73%- 43.7%)	100%(88.4%- 100%)	100% (39.8%- 100%)	65.2%(49.8%- 78.6%)
Group2 30-39 years	.636 (.565- .707)	29.8% (17.3% 44.9%)	97.4%(86.5%- 99.9%)	93.3%(68.1%- 99.8%)	53.5% (41.3%- 65.5%)
Group3 40-49 years	.648 (.585- .712)	33.8% (22.8%- 46.3%)	95.8% (85.7%- 99.5%)	92% (74% - 99%)	50.5% (39.9%- 61.2%)
Group4 50-59 years	.695 (.644- .747)	39.1% (28.8%- 50.1%)	100%(89.7%- 100%)	100%(89.7%- 100%)	39.1% (28.8%- 50.1%)
Group4 >60 years	.714(.606- .821)	48.3% (29.4%- 67.5%)	94.4% (72.7%- 99.9%)	93.3% (68.1%- 99.8%)	53.1% (34.7%- 70.9%)
Cut off	1.3				
Whole cohort	.655(.624- .687)	33.5% (27.7%- 39.7%)	97.6%(94.1%- 99.4%)	95.5%(88.8% - 98.7%)	49.7%(44.2%- 55.2%)
Group1 < 30 years	.6 .(51 -.69)	20%(5.73%- 43.7%)	100%(88.4%- 100%)	100% (39.8%- 100%)	65.2%(49.8%- 78.6%)
Group2 30-39 years	.604 (.538- .67)	23.4% (12.3%- 38%)	97.4% (86.5%- 99.9%)	91.7% (61.5%- 99.8%)	51.4% (39.4%- 63.1%)
Group3 40-49 years	.641(.578- .704)	32.4% (21.5%- 44.8%)	95.8% (85.7%- 99.5%)	91.7%(73%- 99%)	50% (39.4%- 60.6%)
Group4 50-59 years	.69(.638- .741)	37.9% (27.7%- 49%)	100%(89.7%- 100%)	100% (89.4%- 100%)	38.6% (28.4%- 49.6%)
Group4 >60 years	.714(.606- .821)	48.3%(29.4%- 67.5%)	94.4% (72.7%- 99.9%)	93.3% (68.1%- 99.8%)	53.1% (34.7%- 70.9%)

Cut off	1.4				
Whole cohort	.649(.62- .679)	31.1% (25.4%- 37.2%)	98.8% (95.8%- 99.9%)	97.5% (91.3%- 99.7%)	49.1%(43.7%- 54.6%)
Group1 < 30 years	.6 (.51- .69)	20%(5.73%- 43.7%)	100% (88.4%- 100%)	100% (39.8%- 100%)	65.2%(49.8%- 78.6%)
Group2 30-39 years	.617 (.556- .678)	23.4% (12.3% - 38%)	100% (91% - 100%)	100%(71.5%- 100%)	52% (40.2%- 63.7%)
Group3 40-49 years	.629 (.572- .687)	27.9% (17.7%- 40.1%)	97.9% (88.9%- 99.9%)	95% (75.1%- 99.9%)	49%(38.6%- 59.4%)
Group4 50-59 years	.678 (.628- .729)	35.6% (25.6%- 46.6%)	100% (89.7%- 100%)	100%(88.8%- 100%)	37.8% (27.8%- 48.6%)
Group4 >60 years	.696(.589- .803)	44.8% (26.4%- 64.3%)	94.4% (72.7% - 99.9)	92.9% (66.1%- 99.8%)	51.5% (33.5%- 69.2%)

**********************************Ringdiff***********************************

Diagnostic accuracy of ringdiff, All cutoff values , (no DM)..

Age group	ROC	Sensitivity	Specificity	PPV	NPV
Cut off	0.5				
Whole cohort	.739(.699-.778)	58.5% (51.9% -64.9%)	89.2% (83.4%-93.4%)	88.4%(82.3%-93%)	60.4% (54%-66.6%)
Group1 < 30 years	.634 (.526 -.742)	30% (11.9%-54.3%)	96.8% (83.3%-99.9%)	85.7% (42.1%-99.6%)	68.2% (52.4%-81.4%)
Group2 30-39 years	.701(.614-.787)	47.6% (32%-63.6%)	92.5% (79.6%-98.4%)	87% (66.4%-97.2%)	62.7%(49.1%-75%)
Group3 40-49 years	.759 (.686-.831)	60.6% (47.8%-72.4%)	91.1% (78.8%-97.5%)	90.9% (78.3%-97.5%)	61.2%(48.5%-72.9%)
Group4 50-59 years	.705 (.617-.793)	62.2% (50.8%-72.7%)	78.8%(61.1%-91%)	87.9% (76.7%-95%)	45.6% (32.4%-59.3%)
Group4 >60 years	.828 (.708-.949)	83.3%(62.6%-95.3%)	82.4% (56.6%-96.2%)	87%(66.4%-97.2%)	77.8%(52.4%-93.6%)
Cut off	0.6				
Whole cohort	.725 (.689-.761)	50.4%(43.8%-57%)	94.6% (90%-97.5%)	92.9%(87%-96.7%)	57.5% (51.4%-63.4%)
Group1 < 30 years	.584 (.489-.679)	20% (5.73%-43.7%)	96.8%(83.3%-99.9%)	80% (28.4%-99.5%)	65.2%(49.8%-78.6%)
Group2 30-39 years	.714 (.634-.794)	45.2% (29.8%-61.3%)	97.5% (86.8%-99.9%)	95% (75.1%-99.9%)	62.9%(49.7%-74.8%)
Group3 40-49 years	.702 (.631-.772)	47% (34.6%-59.7%)	93.3% (81.7%-98.6%)	91.2%(76.3%-98.1%)	54.5% (42.8%-65.9%)
Group4 50-59 years	.729(.655-.803)	54.9% (43.5%-65.9%)	90.9% (75.7%-98.1%)	93.8% (82.8%-98.7%)	44.8% (32.6%-57.4%)
Group4 >60 years	.866(.765-.967)	79.2%(57.8%-92.9%)	94.1%(71.3%-99.9%)	95% (75.1%-99.9%)	76.2% (52.8%-91.8%)
Cut off	0.7				
Whole cohort	.714(.678-.749)	47% (40.5%-53.6%)	95.8%(91.5%-98.3%)	94% (88.1%-97.6%)	56.2% 50.2% 62%
Group1 < 30 years	.6 (.51- .69)	20%(5.73%-43.7%)	100% (88.8%-100%)	100% (39.8%-100%)	66% (50.7%-79.1%)
Group2 30-39 years	.69 (.611-.769)	40.5% (25.6% -56.7%)	97.5%(86.8%-99.9%)	94.4%(72.7%-99.9%)	60.9% (47.9%-72.9%)
Group3 40-49 years	.679 (.608-.749)	42.4%(30.3%-55.2%)	93.3%(81.7%-98.6%)	90.3% (74.2%-98%)	52.5%(41%-63.8%)
Group4 50-59 years	.723(.649-.797)	53.7% (42.3%-64.7%)	90.9% (75.7%-98.1%)	93.6% (82.5%-98.7%)	44.1% (32.1%-56.7%)
Group4 >60 years	.854(.761-.947)	70.8%(48.9%-87.4%)	100% (80.5%-100%)	100%(80.5%-100%)	70.8% (48.9%-87.4%)

Cut off	0.8				
Whole cohort	.699 (.664-.733)	42.7%(36.3%-49.3%)	97%(93.1%-99%)	95.2%(89.2%-98.4%)	54.6% (48.7%-60.4%)
Group1 < 30 years	.575(.495-.655)	15% (3.21%-37.9%)	100%(88.8%-100%)	100%(29.2%-100%)	64.6%(49.5%-77.8%)
Group2 30-39 years	.69 (.611-.769)	40.5%(25.6%-56.7%)	97.5%(86.8%-99.9%)	94.4% (72.7%-99.9%)	60.9%(47.9%-72.9%)
Group3 40-49 years	.675(.608-.741)	39.4% (27.6%-52.2%)	95.6% (84.9%-99.5%)	92.9%(76.5%-99.1%)	51.8% (40.6%-62.9%)
Group4 50-59 years	.708(.639-.776)	47.6% (36.4%-58.9%)	93.9%(79.8%-99.3%)	95.1% (83.5%-99.4%)	41.9% (30.5%-53.9%)
Group4 >60 years	.813(.714-.911)	62.5% (40.6%-81.2%)	100% (80.5%-100%)	100% (8.2%-100%)	65.4%(44.3%-82.8%)
Cut off	0.9				
Whole cohort	.681(.648-.714)	38% (31.8%-44.6%)	98.2% (94.8%-99.6%)	96.7% (90.8%-99.3%)	52.9%(47.2%-58.6%)
Group1 < 30 years	.575(.495-.655)	15% (3.21%-37.9%)	100% (88.8%-100%)	100%(29.2%-100%)	64.6% (49.5%-77.8%)
Group2 30-39 years	.678 (.6 -.756)	38.1%(23.6%-54.4%)	97.5% (86.8%-99.9%)	94.1%(71.3%-99.9%)	60% (47.1%-72%)
Group3 40-49 years	.663(.601-.725)	34.8% (23.5%-47.6%)	97.8% (88.2%-99.9%)	95.8% (78.9%-99.9%)	50.6%(39.6%-61.5%)
Group4 50-59 years	.698(.637-.76)	42.7% (31.8%-54.1%)	97%(84.2%-99.9%)	97.2% (85.5%-99.9%)	40.5% (29.6%-52.1%)
Group4 >60 years	.75(.648-.852)	50% (29.1%-70.9%)	100% (80.5%-100%)	100% (73.5%-100%)	58.6% (38.9%-76.5%)
Cut off	1.0				
Whole cohort	.682 (.651-.713)	36.3%(30.2%-42.8%)	100%(97.8%-100%)	100%(95.8%-100%)	52.7% (47%-58.3%)
Group1 < 30 years	.575 (.495-.655)	15%(3.21%-37.9%)	100% (88.8%-100%)	100% (29.2%-100%)	64.6%(49.5%-77.8%)
Group2 30-39 years	.679(.605-.752)	35.7% (21.6%-52%)	100% (91.2%-100%)	100% (78.2%-100%)	59.7% (47%-71.5%)
Group3 40-49 years	.667(.609-.724)	33.3%(22.2%-46%)	100% (92.1%-100%)	100% (84.6%-100%)	50.6% (39.8% -61.3%)
Group4 50-59 years	.707(.654-.761)	41.5% (30.7%-52.9%)	100%(89.4%-100%)	100%(89.7%-100%)	40.7% (29.9%-52.2%)
Group4 >60 years	.729(.627-.831)	45.8%(25.6%-67.2%)	100% (80.5%-100%)	100%(71.5%-100%)	56.7%(37.4%-74.5%)
Cut off	1.1				
Whole cohort	.673(.643-.704)	34.6% (28.5%-41.1%)	100%(97.8%-100%)	100%(95.5%-100%)	52% (46.4%-57.6%)

Group1 < 30 years	.575 (.495-.655)	15%(3.21%-37.9%)	100% (88.8%-100%)	100% (29.2%-100%)	64.6% (49.5%-77.8%)
Group2 30-39 years	.679 (.605-.752)	35.7% (21.6%-52%)	100% (91.2%-100%)	100% (78.2%-100%)	59.7% (47%-71.5%)
Group3 40-49 years	.652 (.596-.707)	30.3% (19.6%-42.9%)	100% (92.1%-100%)	100%(83.2%-100%)	49.5%(38.8%-60.1%)
Group4 50-59 years	.701 (.648-.755)	40.2% (29.6%-51.7%)	100%(89.4%-100%)	100%(89.4%-100%)	40.2% (29.6%-51.7%)
Group4 >60 years	.708(.608-.809)	41.7%(22.1%-63.4%)	100% (80.5%-100%)	100%(69.2%-100%)	54.8%(36%-72.7%)
Cut off	1.2				
Whole cohort	.652(.622-.681)	30.3%(24.5%-36.7%)	100%(97.8%-100%)	100%(94.9%-100%)	50.5%(44.9%-56%)
Group1 < 30 years	.575(.495-.655)	15% (3.21%-37.9%)	100%(88.8%-100%)	100% (29.2%-100%)	64.6%(49.5%-77.8%)
Group2 30-39 years	.643 (.574-.712)	28.6%(15.7%-44.6%)	100% (91.2%-100%)	100% (73.5%-100%)	57.1%(44.7%-68.9%)
Group3 40-49 years	.636 (.582-.69)	27.3%(17%-39.6%)	100% (92.1%-100%)	100% 81.5% 100%	48.4% (37.9%-59%)
Group4 50-59 years	.677 (.625-.729)	35.4%(25.1%-46.7%)	100% (89.4%-100%)	100%(88.1%-100%)	38.4(% 28.1%-49.5%)
Group4 >60 years	.688(.589-.786)	37.5% (18.8%-59.4%)	100% (80.5%-100%)	100% (66.4%-100%)	53.1%(34.7%-70.9%)
Cut off	1.3				
Whole cohort	.639(.61-.668)	27.8%(22.1%-34%)	100%(97.8% -100%)	100% (94.5%-100%)	49.6%(44.1%-55%)
Group1 < 30 years	.575 (.495-.655)	15% (3.21%-37.9%)	100%(88.8%-100%)	100% (29.2%-100%)	64.6%(49.5%-77.8%)
Group2 30-39 years	.631 (.564-.698)	26.2%(13.9%-42%)	100% (91.2%-100%)	100% (71.5%-100%)	56.3%(44%-68.1%)
Group3 40-49 years	.636(.582-.69)	27.3% (17%-39.6%)	100%(92.1%-100%)	100%(81.5%-100%)	48.4% (37.9%-59%)
Group4 50-59 years	.659 (.608-.709)	31.7% (21.9%-42.9%)	100%(89.4%-100%)	100% (86.8%-100%)	37.1% (27.1%-48%)
Group4 >60 years	.646(.553-.739)	29.2%(12.6%-51.1%)	100% (80.5%-100%)	100% (59% -100%)	50% (32.4%-67.6%)
Cut off	1.4				
Whole cohort	.628(.6-.656)	25.6% (20.2%-31.7%)	100% (97.8%-100%)	100%(94%-100%)	48.8%(43.4%-54.3%)
Group1 < 30 years	.575 (.495-.655)	15% (3.21%-37.9%)	100% (88.8%-100%)	100%(29.2%-100%)	64.6%(49.5%-77.8%)
Group2 30-39 years	.595 (.535-.655)	19% (8.6%-34.1%)	100%(91.2%-100%)	100%(63.1%-100%)	54.1% (42.1%-65.7%)

Group3 40-49 years	.629 (.576-.682)	25.8%(15.8%-38%)	100% (92.1%-100%)	100% (80.5%-100%)	47.9%(37.5%-58.4%)
Group4 50-59 years	.659 (.608-.709)	31.7% (21.9%-42.9%)	100%(89.4%-100%)	100%(86.8%-100%)	37.1% (27.1%-48%)
Group4 >60 years	.625 (.537-.713)	25% (9.77%-46.7%)	100%(80.5%-100%)	100%(54.1%-100%)	48.6% (31.4%-66%)

********************************CSI*********************************

Diagnostic accuracy of combined sensory index (CSI), All cutoff values , (no DM).

Age group	ROC	Sensitivity	Specificity	PPV	NPV
Cut off	1.0				
Whole cohort	.791 (.75-.831)	79.8% (74.2% - 84.6%)	78.3% (71.1%-84.5%)	85.3%(80%-89.6%)	71.1% (63.7%-77.7%)
Group1 < 30 years	.742(.621-.862)	55% (31.5%-76.9%)	93.3% (77.9%-99.2%)	84.6% (54.6%-98.1%)	75.7% (58.8%-88.2%)
Group2 30-39 years	.719 (.622-.816)	66% (50.7%-79.1%)	77.8%(60.8%-89.9%)	79.5% (63.5%-90.7%)	63.6% (47.8%-77.6%)
Group3 40-49 years	.82(.746-.894)	80.3%(68.7%-89.1%)	83.7% (69.3%-93.2%)	88.3% (77.4%-95.2%)	73.5% (58.9%-85.1%)
Group4 50-59 years	.781 (.694-.868)	86.5%(77.6%-92.8%)	69.7%(51.3%-84.4%)	88.5% (79.9%-94.3%)	65.7% (47.8%-80.9%)
Group4 >60 years	.767 (.636-.897)	100% (86.3%-100%)	53.3%(26.6%-78.7%)	78.1% (60%-90.7%)	100% (63.1%-100%)
Cut off	1.1				
Whole cohort	.798(.758-.838)	78.1% (72.5%-83.1%)	81.5%(74.6%-87.3%)	86.9% (81.8%-91.1%)	70.3% (63.1%-76.9%)
Group1 < 30 years	.692 (.571 -.812)	45%(23.1%-68.5%)	93.3%(77.9%-99.2%)	81.8% (48.2%-97.7%)	71.8% (55.1%-85%)
Group2 30-39 years	.746(.654-.839)	66% (50.7%-79.1%)	83.3%(67.2%-93.6%)	83.8% (68%-93.8%)	65.2% (49.8%-78.6%)
Group3 40-49 years	.813 (.738-.887)	78.8%(67%-87.9%)	83.7% (69.3%-93.2%)	88.1% (77.1%-95.1%)	72% (57.5%-83.8%)
Group4 50-59 years	.806 (.723-.889)	85.4% (76.3%-92%)	75.8% (57.7% 88.9%)	90.5% (82.1%-95.8%)	65.8% (48.6%-80.4%)
Group4 >60 years	.8 (.672-.928)	100% (86.3%-100%)	60% (32.3%-83.7%)	80.6% (62.5%-92.5%)	100% (66.4%-100%)
Cut off	1.2				
Whole cohort	.799(.76-.838)	75.7% (69.9%-80.9%)	84.1%(77.4%-89.4%)	88.2%(83.1%-92.2%)	68.8%(61.7%-75.2%)
Group1 < 30 years	.708 (.592-.825)	45%(23.1%-68.5%)	96.7% (82.8%-99.9%)	90%(55.5%-99.7%)	72.5%(56.1%-85.4%)
Group2 30-39 years	.764 (.677-.85)	63.8% (48.5%-77.3%)	88.9%(73.9%-96.9%)	88.2% (72.5%-96.7%)	65.3% (50.4%-78.3%)
Group3 40-49 years	.79 (.713-.867)	74.2% (62%-84.2%)	83.7% (69.3%-93.2%)	87.5% (75.9%-94.8%)	67.9%(53.7%-80.1%)
Group4 50-59 years	.815 (.735-.896)	84.3% (75%-91.1%)	78.8% (61.1%-91%)	91.5%(83.2%-96.5%)	65% (48.3%-79.4%)

Group4 >60 years	.78 (.646- .914)	96%(79.6%- 99.9%)	60%(32.3%- 83.7%)	80%(61.4%- 92.3%)	90%(55.5%- 99.7%)
Cut off	1.3				
Whole cohort	.804(.765- .842)	74.1%(68.2%- 79.4%)	86.6%(80.3%- 91.5%)	89.7%(84.7%- 93.5%)	68% (61.1%- 74.4%)
Group1 < 30 years	.708 (.592- .825)	45%(23.1%- 68.5%)	96.7%(82.8%- 99.9%)	90% (55.5%- 99.7%)	72.5%(56.1%- 85.4%)
Group2 30-39 years	.756 (.672- .841)	59.6% (44.3%- 73.6%)	91.7%(77.5%- 98.2%)	90.3%(74.2%- 98%)	63.5% (49%- 76.4%)
Group3 40-49 years	.801 (.727- .876)	74.2% (62%- 84.2%)	86% (72.1%- 94.7%)	89.1% (77.8%- 95.9%)	68.5% (54.4%- 80.5%)
Group4 50-59 years	.819 (.741- .897)	82% (72.5%- 89.4%)	81.8%(64.5%- 93%)	92.4%(84.2%- 97.2%)	62.8%(46.7%- 77%)
Group4 >60 years	.813(.684- .943)	96% (79.6%- 99.9%)	66.7% (38.4%- 88.2%)	82.8% (64.2%- 94.2%)	90.9% (58.7%- 99.8%)
Cut off	1.4				
Whole cohort	.792(.753- .83)	70.4% (64.3%- 76.1%)	87.9% (81.7%- 92.6%)	90.2%(85.1%- 94%)	65.4% (58.6%- 71.8%)
Group1 < 30 years	.7 (.59- .81)	40% (19.1%- 63.9%)	100% (88.4%- 100%)	100% (63.1%- 100%)	71.4% (55.4%- 84.3%)
Group2 30-39 years	.735 (.65- .82)	55.3%(40.1%- 69.8%)	91.7% (77.5%- 98.2%)	89.7%(72.6%- 97.8%)	61.1% (46.9%- 74.1%)
Group3 40-49 years	.79(.716- .864)	69.7% (57.1%- 80.4%)	88.4%(74.9%- 96.1%)	90.2% (78.6%- 96.7%)	65.5% (51.9%- 77.5%)
Group4 50-59 years	.802 (.723- .882)	78.7%(68.7%- 86.6%)	81.8%(64.5%- 93%)	92.1%(83.6%- 97%)	58.7% (43.2%- 73%)
Group4 >60 years	.813(.684- .943)	96% (79.6%- 99.9%)	66.7%(38.4%- 88.2%)	82.8%(64.2%- 94.2%)	90.9% (58.7%- 99.8%)
Cut off	1.5				
Whole cohort	.782 (.745- .82)	66%(59.7%- 71.9%)	90.4%(84.7%- 94.6%)	91.6% (86.5%- 95.2%)	62.8% (56.2%- 69.1%)
Group1 < 30 years	.65(.547- .753)	30% (11.9%- 54.3%)	100% (88.4%- 100%)	100% (54.1%- 100%)	68.2% (52.4%- 81.4%)
Group2 30-39 years	.741(.664- .819)	51.1% (36.1%- 65.9%)	97.2% (85.5%- 99.9%)	96% (79.6%- 99.9%)	60.3%(46.6%- 73%)
Group3 40-49 years	.775 (.7 -.85)	66.7% (54%- 77.8%)	88.4%(74.9%- 96.1%)	89.8% (77.8%- 96.6%)	63.3%(49.9%- 75.4%)
Group4 50-59 years	.789 (.712- .867)	73%(62.6%- 81.9%)	84.8%(68.1%- 94.9%)	92.9% (84.1%- 97.6%)	53.8% (39.5%- 67.8%)
Group4 >60 years	.847 (724- .969)	96% (79.6%- 99.9%)	73.3%(44.9%- 92.2%)	85.7%(67.3%- 96%)	91.7%(61.5%- 99.8%)
Cut off	1.6				

Whole cohort	.772 (.734-.81)	64%(57.6%- 70%)	90.4% (84.7%-94.6%)	91.3% (86.1%-95.1%)	61.5% (54.9%-67.8%)
Group1 < 30 years	.65 (.547-.753)	30%(11.9%-54.3%)	100%(88.4%-100%)	100%(54.1%-100%)	68.2% (52.4%-81.4%)
Group2 30-39 years	.731(.654-.808)	48.9% (34.1%-63.9%)	97.2% (85.5%-99.9%)	95.8% (78.9%-99.9%)	59.3% (45.7%-71.9%)
Group3 40-49 years	.752 (.676-.829)	62.1% (49.3%-73.8%)	88.4% (74.9%-96.1%)	89.1% (76.4%-96.4%)	60.3% (47.2%-72.4%)
Group4 50-59 years	.784 (.706-.862)	71.9% (61.4%-80.9%)	84.8%(68.1%-94.9%)	92.8%(83.9%-97.6%)	52.8% (38.6%-66.7%)
Group4 >60 years	.847 (.724-.969)	96% (79.6%-99.9%)	73.3%(44.9%-92.2%)	85.7%(67.3%-96%)	91.7% (61.5%-99.8%)
Cut off	1.7				
Whole cohort	.768(.731-.806)	61.9%(55.6%-68%)	91.7% (86.3%-95.5%)	92.2%(87%-95.8%)	60.5% (54%-66.8%)
Group1 < 30 years	.6 (.51- .69)	20% (5.73%-43.7%)	100% (88.4%-100%)	100% (39.8%-100%)	65.2%(49.8%-78.6%)
Group2 30-39 years	.731(.654-.808)	48.9% (34.1%-63.9%)	97.2%(85.5% -99.9%)	95.8% (78.9%-99.9%)	59.3%(45.7%-71.9%)
Group3 40-49 years	.764 (.691-.838)	62.1% (49.3%-73.8%)	90.7% (77.9%-97.4%)	91.1% (78.8%-97.5%)	60.9% (47.9%-72.9%)
Group4 50-59 years	.782 (.708-.857)	68.5%(57.8%-78%)	87.9% (71.8%-96.6%)	93.8%(85%-98.3%)	50.9% (37.3%-64.4%)
Group4 >60 years	.847 (.724-.969)	96% (79.6%-99.9%)	73.3% (44.9%-92.2%)	85.7% (67.3%-96%)	91.7% (61.5%-99.8%)
Cut off	2.0				
Whole cohort	.753 (.718-.788)	55.1% (48.6%-61.4%)	95.5% (91%-98.2%)	95.1%(90.2%-98%)	57.5% (51.2%-63.5%)
Group1 < 30 years	.6 (.51- .69)	20% (5.73%-43.7%)	100% (88.4%-100%)	100% (39.8%-100%)	65.2%(49.8%-78.6%)
Group2 30-39 years	.72 (.643-.797)	46.8% (32.1%-61.9%)	97.2%(85.5%-99.9%)	95.7% (78.1%-99.9%)	58.3% (44.9%-70.9%)
Group3 40-49 years	.719 (.644-.794)	53% (40.3%-65.4%)	90.7%(77.9%-97.4%)	89.7% (75.8%-97.1%)	55.7%(43.3%-67.6%)
Group4 50-59 years	.773 (.707-.839)	60.7% (49.7%-70.9%)	93.9% (79.8%-99.3%)	96.4%(87.7%-99.6%)	47% (34.6%-59.7%)
Group4 >60 years	.92 (.847-.993)	84% (63.9%-95.5%)	100% (78.2%-100%)	100%(83.9%-100%)	78.9%(54.4%-93.9%)
Cut off	2.5				
Whole cohort	.709 (.676-.742)	43.7%(37.4%-50.2%)	98.1% (94.5%-99.6%)	97.3% (92.3%-99.4%)	52.6% (46.7%-58.4%)
Group1 < 30 years	.6 (.51- .69)	20%(5.73%-43.7%)	100% (88.4%-100%)	100% (39.8%-100%)	65.2%(49.8%-78.6%)

Group2 30-39 years	.688 (.612-.764)	40.4% (26.4%-55.7%)	97.2% (85.5%-99.9%)	95%(75.1%-99.9%)	55.6% (42.5%-68.1%)
Group3 40-49 years	.696 (.628-.765)	43.9% (31.7%-56.7%)	95.3% (84.2%-99.4%)	93.5% (78.6%-99.2%)	52.6% (40.9%-64%)
Group4 50-59 years	.725 (.673-.777)	44.9%(34.4%-55.9%)	100%(89.4%-100%)	100% (91.2%-100%)	40.2% (29.6%-51.7%)
Group4 >60 years	.82 (.724-.916)	64% (42.5% -82%)	100% (78.2%-100%)	100% (79.4%-100%)	62.5% (40.6%-81.2%)
Cut off	3.0				
Whole cohort	.68 (.65 -.71)	36% (30% -42.4%)	100% (97.7%-100%)	100% (95.9%-100%)	49.8%(44.2%-55.5%)
Group1 < 30 years	.575 (.495-.655)	15% (3.21%-37.9%)	100% (88.4%-100%)	100% (29.2%-100%)	63.8% (48.5%-77.3%)
Group2 30-39 years	.66 (.592-.727)	31.9% (19.1%-47.1%)	100% (90.3%-100%)	100% (78.2%-100%)	52.9%(40.4%-65.2%)
Group3 40-49 years	.674(.616-.732)	34.8%(23.5%-47.6%)	100% (91.8%-100%)	100% (85.2%-100%)	50% (39% -61%)
Group4 50-59 years	.685 (.635-.736)	37.1% (27.1%-48%)	100% (89.4%-100%)	100%(89.4%-100%)	37.1% (27.1%-48%)
Group4 >60 years	.8 (.702-.898)	60% (38.7%-78.9%)	100%(78.2%-100%)	100% (78.2%-100%)	60% (38.7%-78.9%)
