# Supplementary material for: Values and Diagnostic Accuracy of Electrodiagnostic Findings in Carpal Tunnel Syndrome Based on Age, Gender, and Diabetes
Source: Diagnostics (Basel). 2024 Jun 28;14(13):1381. doi: 10.3390/diagnostics14131381 (PMC11240809; doi:10.3390/diagnostics14131381)
Supplement: Supplementary file 1 [file diagnostics-14-01381-s001.zip › Table S6 Diagnostic accuracy of median latency at Digit II and comparative latency studies (COLSs) , all cutoff values (participants with DM);.pdf]

Table S6 Diagnostic accuracy of median latency at Digit II, All cutoff values, (participants with DM)

Age group	ROC	Sensitivity	Specificity	PPV	NPV
Cut off	3.5 (ms)				
Whole cohort	.668 (.587-.749)	84.6% (74.7%-91.8%)	49% (34.4%-63.7%)	72.5% (62.2%-81.4%)	66.7% (49%-81.4%)
Group1 < 30 years	.75 (0.0- 1)	50% (1.26%-98.7%)	100% (2.5%-100%)	100% (2.5%-100%)	50% (1.26%-98.7%)
Group2 30-39 years	.667 (0.0-1)	33.3% (.84%-90.6%)	100% (2.5%-100%)	100% (2.5%-100%)	33.3% (.84%-90.6%)
Group3 40-49 years	.564 (.287-.841)	72.7% (39%-94%)	40% (5.27%-85.3%)	72.7% (39%-94%)	40% (5.27%-85.3%)
Group4 50-59 years	.708 (.574-.841)	88.6% (73.3%-96.8%)	52.9% (27.8%-77%)	79.5% (63.5%-90.7%)	69.2% (38.6%-90.9%)
Group4 >60 years	.683 (.572-.794)	92.6% (75.7%-99.1%)	44% (24.4%-65.1%)	64.1% (47.2%-78.8%)	84.6% (54.6%-98.1%)
Cut off	3.6 (ms)				
Whole cohort	.64 (.555-.725)	76.9% (66%-85.7%)	51% (36.3%-65.6%)	71.4% (60.5%-80.8%)	58.1% (42.1%-73%)
Group1 < 30 years	.75 (.-1)	50% (1.26%-98.7%)	100% (2.5%-100%)	100% (2.5%-100%)	50% (1.26%-98.7%)
Group2 30-39 years	.667 (.-1)	33.3% (.84%-90.6%)	100% (2.5%-100%)	100% (2.5%-100%)	33.3% (.84%-90.6%)
Group3 40-49 years	.518 (.236-.801)	63.6% (30.8%-89.1%)	40% (5.27%-85.3%)	70% (34.8%-93.3%)	33.3% (4.33%-77.7%)
Group4 50-59 years	.65 (.509-.792)	77.1% (59.9%-89.6%)	52.9% (27.8%-77%)	77.1% (59.9%-89.6%)	52.9% (27.8%-77%)
Group4 >60 years	.684 (.568-.801)	88.9% (70.8%-97.6%)	48% (27.8%-68.7%)	64.9% (47.5%-79.8%)	80% (51.9%-95.7%)
Cut off	3.7 (ms)				
Whole cohort	.664 (.579-.749)	75.6% (64.6%-84.7%)	57.1% (42.2%-71.2%)	73.8% (62.7%-83%)	59.6% (44.3%-73.6%)
Group1 < 30 years	.75 (.-1)	50% (1.26%-98.7%)	100% (2.5%-100%)	100% (2.5%-100%)	50% (1.26%-98.7%)
Group2 30-39 years	.667 (.-1)	33.3% (.84%-90.6%)	100% (2.5%-100%)	100% (2.5%-100%)	33.3% (.84%-90.6%)
Group3 40-49 years	.618 (.336-.901)	63.6% (30.8%-89.1%)	60% (14.7%-94.7%)	77.8% (40%-97.2%)	42.9% (9.9%-81.6%)
Group4 50-59 years	.68 (.54-.82)	77.1% (59.9%-89.6%)	58.8% (32.9%-81.6%)	79.4% (62.1%-91.3%)	55.6% (30.8%-78.5%)

Group4 >60 years	.686 (.565-.807)	85.2% (66.3%-95.8%)	52% (31.3%-72.2%)	65.7% (47.8%-80.9%)	76.5% (50.1%-93.2%)
Cut off	3.9				
Whole cohort	.665 (.58-.75)	71.8% (60.5%-81.4%)	61.2% (46.2%-74.8%)	74.7% (63.3%-84%)	57.7% (43.2%-71.3%)
Group1 < 30 years	.75 (.-1)	50% (1.26%-98.7%)	100% (2.5%-100%)	100% (2.5%-100%)	50% (1.26%-98.7%)
Group2 30-39 years	.667 (.-1)	33.3% (.84%-90.6%)	100% (2.5%-100%)	100% (2.5%-100%)	33.3% (.84%-90.6%)
Group3 40-49 years	.718 (.472-.964)	63.6% (30.8%-89.1%)	80% (28.4%-99.5%)	87.5% (47.3%-99.7%)	50% (15.7%-84.3%)
Group4 50-59 years	.681 (.541-.82)	71.4% (53.7%-85.4%)	64.7% (38.3%-85.8%)	80.6% (62.5%-92.5%)	52.4% (29.8%-74.3%)
Group4 >60 years	.667 (.543-.792)	81.5% (61.9%-93.7%)	52% (31.3%-72.2%)	64.7% (46.5%-80.3%)	72.2% (46.5%-90.3%)
Cut off	4.0				
Whole cohort	.667 (.583-.752)	64.1% (52.4%-74.7%)	69.4% (54.6%-81.7%)	76.9% (64.8%-86.5%)	54.8% (41.7%-67.5%)
Group1 < 30 years	.75 (.-1)	50% (1.26%-98.7%)	100% (2.5%-100%)	100% (2.5%-100%)	50% (1.26%-98.7%)
Group2 30-39 years	.667 (.-1)	33.3% (.84%-90.6%)	100% (2.5%-100%)	100% (2.5%-100%)	33.3% (.84%-90.6%)
Group3 40-49 years	.673 (.423-.922)	54.5% (23.4%-83.3%)	80% (28.4%-99.5%)	85.7% (42.1%-99.6%)	44.4% (13.7%-78.8%)
Group4 50-59 years	.711 (.58-.842)	65.7% (47.8%-80.9%)	76.5% (50.1%-93.2%)	85.2% (66.3%-95.8%)	52% (31.3%-72.2%)
Group4 >60 years	.652 (.52-.783)	70.4% (49.8%-86.2%)	60% (38.7%-78.9%)	65.5% (45.7%-82.1%)	65.2% (42.7%-83.6%)
Cut off	4.1				
Whole cohort	.679 (.597-.761)	60.3% (48.5%-71.2%)	75.5% (61.1%-86.7%)	79.7% (67.2%-89%)	54.4% (41.9%-66.5%)
Group1 < 30 years					
Group2 30-39 years	.667 (.-1)	33.3% (.84%-90.6%)	100% (2.5%-100%)	100% (2.5%-100%)	33.3% (.84%-90.6%)
Group3 40-49 years	.773 (.618-.927)	54.5% (23.4%-83.3%)	100% (47.8%-100%)	100% (54.1%-100%)	50% (18.7%-81.3%)
Group4 50-59 years	.697 (.565-.829)	62.9% (44.9%-78.5%)	76.5% (50.1%-93.2%)	84.6% (65.1%-95.6%)	50% (29.9%-70.1%)
Group4 >60 years	.673 (.543-.803)	66.7% (46%-83.5%)	68% (46.5%-85.1%)	69.2% (48.2%-85.7%)	65.4% (44.3%-82.8%)
Cut off	4.2				

Whole cohort	.663 (.582-.744)	55.1% (43.4%-66.4%)	77.6% (63.4%-88.2%)	79.6% (66.5%-89.4%)	52.1% (40%-63.9%)
Group1 < 30 years					
Group2 30-39 years					
Group3 40-49 years	.773 (.618-.927)	54.5% (23.4%-83.3%)	100% (47.8%-100%)	100% (54.1%-100%)	50% (18.7%-81.3%)
Group4 50-59 years	.682 (.55-.815)	60% (42.1%-76.1%)	76.5% (50.1%-93.2%)	84% (63.9%-95.5%)	48.1% (28.7%-68.1%)
Group4 >60 years	.656 (.526-.787)	59.3% (38.8%-77.6%)	72% (50.6%-87.9%)	69.6% (47.1%-86.8%)	62.1% (42.3%-79.3%)
Cut off	4.3				
Whole cohort	.691 (.617-.766)	52.6% (40.9%-64%)	85.7% (72.8%-94.1%)	85.4% (72.2%-93.9%)	53.2% (41.6%-64.5%)
Group1 < 30 years					
Group2 30-39 years					
Group3 40-49 years	.727 (.573-.882)	45.5% (16.7%-76.6%)	100% (47.8%-100%)	100% (47.8%-100%)	45.5% (16.7%-76.6%)
Group4 50-59 years	.697 (.572-.823)	57.1% (39.4%-73.7%)	82.4% (56.6%-96.2%)	87% (66.4%-97.2%)	48.3% (29.4%-67.5%)
Group4 >60 years	.716 (.597-.836)	59.3% (38.8%-77.6%)	84% (63.9%-95.5%)	80% (56.3%-94.3%)	65.6% (46.8%-81.4%)
Cut off	4.4				
Whole cohort	.689 (.616-.761)	50% (38.5%-61.5%)	87.8% (75.2%-95.4%)	86.7% (73.2%-94.9%)	52.4% (41.1%-63.6%)
Group1 < 30 years					
Group2 30-39 years					
Group3 40-49 years	.727 (.573-.882)	45.5% (16.7%-76.6%)	100% (47.8%-100%)	100% (47.8%-100%)	45.5% (16.7%-76.6%)
Group4 50-59 years	.727 (.612-.842)	57.1% (39.4%-73.7%)	88.2% (63.6%-98.5%)	90.9% (70.8%-98.9%)	50% (31.3%-68.7%)
Group4 >60 years	.679 (.558-.8)	51.9% (31.9%-71.3%)	84% (63.9%-95.5%)	77.8% (52.4%-93.6%)	61.8% (43.6%-77.8%)
Cut off	4.5				
Whole cohort	.68 (.61-.75)	46.2% (34.8%-57.8%)	89.8% (77.8%-96.6%)	87.8% (73.8%-95.9%)	51.2% (40.1%-62.1%)
Group1 < 30 years					

Group2 30-39 years					
Group3 40-49 years	.727 (.573-.882)	45.5% (16.7%-76.6%)	100% (47.8%-100%)	100% (47.8%-100%)	45.5% (16.7%-76.6%)
Group4 50-59 years	.698 (.583-.814)	51.4% (34%-68.6%)	88.2% (63.6%-98.5%)	90% (68.3%-98.8%)	46.9% (29.1%-65.3%)
Group4 >60 years	.681 (.565-.797)	48.1% (28.7%-68.1%)	88% (68.8%-97.5%)	81.3% (54.4%-96%)	61.1% (43.5%-76.9%)

******************************Palmdiff*********************************

Diagnostic accuracy of palmdiff, All cutoff values, (participants with DM).

Age group	ROC	Sensitivity	Specificity	PPV	NPV
Cut off	0.5				
Whole cohort	.683(.593-.773)	74.6% (62.9% -84.2%)	61.9%(45.6%-76.4%)	76.8% (65.1%-86.1%)	59.1%(43.2%-73.7%)
Group1 < 30 years	.75(. 1-	50% (1.26%-98.7%)	100%(2.5%-100%)	100%(2.5%-100%)	50%(1.26%-98.7%)
Group2 30-39 years	.833(. 1-	66.7% (9.43%-99.2%)	100% (2.5%-100%)	100% (15.8%-100%)	50% (1.26%-98.7%)
Group3 40-49 years	.929 (.833- 1)	85.7% (57.2%-98.2%)	100% (39.8%-100%)	100%(73.5% -100%)	66.7% (22.3%-95.7%)
Group4 50-59 years	.593(.429-.756)	72.4% (52.8%-87.3%)	46.2% (19.2%-74.9%)	75% (55.1%-89.3%)	42.9%(17.7%-71.1%)
Group4 >60 years	.674 (.537-.811)	73.9% (51.6%-89.8%)	60.9%(38.5%-80.3%)	65.4%(44.3%-82.8%)	70% (45.7%-88.1%)
Cut off	0.6				
Whole cohort	.709 (.622-.797)	70.4%(58.4%-80.7%)	71.4% (55.4%-84.3%)	80.6% (68.6%-89.6%)	58.8% (44.2%-72.4%)
Group1 < 30 years	.75(. 1-	50% (1.26%-98.7%)	100%(2.5%-100%)	100% (2.5%-100%)	50%(1.26%-98.7%)
Group2 30-39 years	.667 (. 1-	33.3% (.84%-90.6%)	100%(2.5%-100%)	100%(2.5%-100%)	33.3% (.84%-90.6%)
Group3 40-49 years	.929(.833- 1)	85.7% (57.2%-98.2%)	100% (39.8%-100%)	100% (73.5%-100%)	66.7% (22.3%-95.7%)
Group4 50-59 years	.597 (.431-.763)	65.5%(45.7%-82.1%)	53.8% (25.1%-80.8%)	76% (54.9%-90.6%)	41.2% (18.4%-67.1%)
Group4 >60 years	.739(.609-.869)	73.9% (51.6%-89.8%)	73.9% (51.6%-89.8%)	73.9% (51.6%-89.8%)	73.9%(51.6%-89.8%)
Cut off	0.7				
Whole cohort	.707(.62-.794)	67.6% (55.5%-78.2%)	73.8%(58%-86.1%)	81.4%(69.1%-90.3%)	57.4%(43.2%-70.8%)
Group1 < 30 years	.75(. 1-	50% (1.26%-98.7%)	100%(2.5%-100%)	100%(2.5%-100%)	50% (1.26%-98.7%)
Group2 30-39 years					
Group3 40-49 years	.893(.781- 1)	78.6% (49.2%-95.3%)	100% (39.8%-100%)	100% (71.5%-100%)	57.1% (18.4%-90.1%)
Group4 50-59 years	.635(.472-.799)	65.5% (45.7%-82.1%)	61.5% (31.6%-86.1%)	79.2%(57.8%-92.9%)	44.4%(21.5%-69.2%)
Group4 >60 years	.739(.609-.869)	73.9% (51.6%-89.8%)	73.9% (51.6%-89.8%)	73.9%(51.6%-89.8%)	73.9%(51.6%-89.8%)

Cut off	0.8				
Whole cohort	.691(.604-.777)	62% (49.7%-73.2%)	76.2% (60.5%-87.9%)	81.5% (68.6%-90.7%)	54.2%(40.8%-67.3%)
Group1 < 30 years					
Group2 30-39 years					
Group3 40-49 years	.857 (.734-.98)	71.4%(41.9%-91.6%)	100%(39.8%-100%)	100%(69.2% -100%)	50%(15.7%-84.3%)
Group4 50-59 years	.635(.472-.799)	65.5%(45.7%-82.1%)	61.5%(31.6%-86.1%)	79.2% (57.8%-92.9%)	44.4%(21.5%-69.2%)
Group4 >60 years	.717(.586-.849)	65.2% (42.7%-83.6%)	78.3% (56.3%-92.5%)	75%(50.9%-91.3%)	69.2% (48.2% -85.7%)
Cut off	0.9				
Whole cohort	.71(.631-.789)	56.3%(44%-68.1%)	85.7% (71.5%-94.6%)	87% (73.7%-95.1%)	53.7%(41.1%-66%)
Group1 < 30 years					
Group2 30-39 years					
Group3 40-49 years	.857 (.734-.98)	71.4%(41.9%-91.6%)	100%(39.8%-100%)	100% (69.2%-100%)	50%(15.7%-84.3%)
Group4 50-59 years	.699 (.561-.836)	55.2%(35.7%-73.6%)	84.6% (54.6%-98.1%)	88.9% (65.3%-98.6%)	45.8% (25.6%-67.2%)
Group4 >60 years	.717 (.588-.846)	60.9% (38.5%-80.3%)	82.6%(61.2%-95%)	77.8% (52.4%-93.6%)	67.9% (47.6%-84.1%)
Cut off	1.0				
Whole cohort	.701 (.624-.778)	52.1% (39.9%-64.1%)	88.1% (74.4%-96%)	88.1% (74.4%-96%)	52.1% (39.9%-64.1%)
Group1 < 30 years					
Group2 30-39 years					
Group3 40-49 years	.857 (.734-.98)	71.4% (41.9%-91.6%)	100% (39.8%-100%)	100% (69.2%-100%)	50%(15.7%-84.3%)
Group4 50-59 years	.664(.527-.802)	48.3%(29.4%-67.5%)	84.6% (54.6%-98.1%)	87.5%(61.7%-98.4%)	42.3%(23.4%-63.1%)
Group 2+3+4					
Group4 >60 years	.717 (.592-.843)	56.5% (34.5% -76.8%)	87% (66.4%-97.2%)	81.3% (54.4%-96%)	66.7% (47.2%-82.7%)
Cut off	1.1				
Whole cohort	.687(.61 -.764)	49.3%(37.2%-61.4%)	88.1%(74.4%-96%)	87.5% (73.2%-95.8%)	50.7%(38.7%-62.6%)

Group1 < 30 years					
Group2 30-39 years					
Group3 40-49 years	.857(.734-.98)	71.4% (41.9%-91.6%)	100% (39.8%-100%)	100%(69.2%-100%)	50% (15.7%-84.3%)
Group4 50-59 years	.664 (.527-.802)	48.3% (29.4% -67.5%)	84.6% (54.6%-98.1%)	87.5% (61.7%-98.4%)	42.3% (23.4%-63.1%)
Group4 >60 years	.674 (.548-.8)	47.8% (26.8%-69.4%)	87%(66.4%-97.2%)	78.6%(49.2%-95.3%)	62.5% (43.7%-78.9%)
Cut off	1.2				
Whole cohort	.69(.619-.76)	45.1% (33.2%-57.3%)	92.9% (80.5%-98.5%)	91.4% (76.9%-98.2%)	50% (38.5%-61.5%)
Group1 < 30 years					
Group2 30-39 years					
Group3 40-49 years	.821 (.691-.952)	64.3%(35.1%-87.2%)	100%(39.8%-100%)	100%(66.4%-100%)	44.4%(13.7%-78.8%)
Group4 50-59 years	.703(.584-.822)	48.3% (29.4%-67.5%)	92.3%(64% -99.8%)	93.3%(68.1%-99.8%)	44.4% (25.5%-64.7%)
Group4 >60 years	.652(.534-.77)	39.1% (19.7%-61.5%)	91.3%(72%-98.9%)	81.8%(48.2%-97.7%)	60% (42.1%-76.1%)
Cut off	1.3				
Whole cohort	.683(.612-.753)	43.7% (31.9%-56%)	92.9%(80.5%-98.5%)	91.2%(76.3%-98.1%)	49.4%(37.9%-60.9%)
Group1 < 30 years					
Group2 30-39 years					
Group3 40-49 years	.821(.691-.952)	64.3% (35.1%-87.2%)	100%(39.8%-100%)	100% (66.4%-100%)	44.4% (13.7%-78.8%)
Group4 50-59 years	.686 (.567-.805)	44.8%(26.4% -64.3%)	92.3%(64%-99.8%)	92.9%(66.1%-99.8%)	42.9% (24.5%-62.8%)
Group4 >60 years	.652(.534-.77)	39.1% (19.7%-61.5%)	91.3%(72%-98.9%)	81.8% (48.2%-97.7%0	60%(42.1%-76.1%)
Cut off	1.4				
Whole cohort	.652 (.588-.717)	35.2% (24.2%-47.5%)	95.2% (83.8%-99.4%)	92.6%(75.7%-99.1%)	46.5% (35.7%-57.6%)
Group1 < 30 years					
Group2 30-39 years					

Group3 40-49 years	.786(.651-.92)	57.1% (28.9%-82.3%)	100%(39.8%-100%)	100%(63.1%-100%)	40% (12.2%-73.8%)
Group4 50-59 years	.617(.503-.731)	31% (15.3%-50.8%)	92.3% (64%-99.8%)	90% (55.5%-99.7%)	37.5%(21.1%-56.3%)
Group4 >60 years	.652(.544-.76)	34.8% (16.4%-57.3%)	95.7% (78.1%-99.9%)	88.9% (51.8%-99.7%)	59.5%(42.1%-75.2%)
Cut off	1.5				
Whole cohort	.645(.581-.709)	33.8% (23% -46%)	95.2% (83.8%-99.4%)	92.3%(74.9%-99.1%)	46%(35.2%-57%)
Group1 < 30 years					
Group2 30-39 years					
Group3 40-49 years	.786(.651-.92)	57.1%(28.9%-82.3%)	100% (39.8%-100%)	100% (63.1%-100%)	40% (12.2%-73.8%)
Group4 50-59 years	.617 (.503-.731)	31%(15.3%-50.8%)	92.3%(64%-99.8%)	90% (55.5%-99.7%)	37.5%(21.1%-56.3%)
Group4 >60 years	.63 (.525-.736)	30.4% (13.2%-52.9%)	95.7% (78.1%-99.9%)	87.5% (47.3%-99.7%)	57.9%(40.8%-73.7%)
Cut off	1.6				
Whole cohort	.624(.561-.687)	29.6%(19.3%-41.6%)	95.2%(83.8%-99.4%)	91.3% (72%-98.9%)	44.4%(34%-55.3%)
Group1 < 30 years					
Group2 30-39 years					
Group3 40-49 years	.786 (.651-.92)	57.1%(28.9%-82.3%)	100% (39.8%-100%)	100%(63.1%-100%)	40% (12.2%-73.8%)
Group4 50-59 years	.599(.488-.711)	27.6%(12.7%-47.2%)	92.3%(64%-99.8%)	88.9%(51.8%-99.7%)	36.4% (20.4%-54.9%)
Group4 >60 years	.587(.491-.683)	21.7% (7.46%-43.7%)	95.7%(78.1%-99.9%)	83.3%(35.9%-99.6%0	55% (38.5%-70.7%)
Cut off	1.7				
Whole cohort	.608 (.553-.663)	23.9%(14.6%-35.5%)	97.6% (87.4%-99.9%)	94.4%(72.7%-99.9%)	43.2% (33%-53.7%)
Group1 < 30 years					
Group2 30-39 years					
Group3 40-49 years	.714 (.58-.849)	42.9% (17.7%-71.1%)	100% (39.8%-100%)	100%(54.1%-100%)	33.3% (9.92%-65.1%)

Group4 50-59 years	.621(.541-.7)	24.1%(10.3%-43.5%)	100% (75.3%-100%)	100% (59%-100%)	37.1% (21.5%-55.1%)
Group4 >60 years	.565 (.475-.655)	17.4%(4.95%-38.8%)	95.7%(78.1%-99.9%)	80% (28.4%-99.5%)	53.7%(37.4%-69.3%)

**********************************Thumbdiff***********************************

Diagnostic accuracy of thumbdiff, All cutoff values, (participants with DM).

Age group	ROC	Sensitivity	Specificity	PPV	NPV
Cut off	0.8				
Whole cohort	.717 (.632-.803)	78.3%(66.7%-87.3%)	65.2%(49.8%-78.6%)	77.1%(65.6%-86.3%)	66.7% (51%-80%)
Group1 < 30 years	1(. 1-	100% (15.8%-100%)	100%(2.5%-100%)	100%(15.8%-100%)	100%(2.5%-100%)
Group2 30-39 years	.833 (. 1-	66.7%(9.43%-99.2%)	100%(2.5%-100%)	100% (15.8%-100%)	50%(1.26%-98.7%)
Group3 40-49 years	.685 (.417-.953)	76.9%(46.2%-95%)	60% (14.7%-94.7%)	83.3%(51.6%-97.9%)	50%(11.8%-88.2%)
Group4 50-59 years	.777(.641-.914)	82.1% (63.1%-93.9%)	73.3%(44.9%-92.2%)	85.2% (66.3%-95.8%)	68.8% (41.3%-89%)
Group4 >60 years	.661 (.525-.797)	73.9% (51.6%-89.8%)	58.3% (36.6%-77.9%)	63% (42.4%-80.6%)	70% (45.7%-88.1%)
Cut off	0.9				
Whole cohort	.721(.636-.806)	76.8% (65.1%-86.1%)	67.4% (52%-80.5%)	77.9% (66.2%-87.1%)	66% (50.7%-79.1%)
Group1 < 30 years	1(. 1-	100%(15.8%-100%)	100%(2.5%-100%)	100% (15.8%-100%)	100%(2.5%-100%)
Group2 30-39 years	.833(. 1-	66.7% (9.43%-99.2%)	100%(2.5%-100%)	100% (15.8%-100%)	50% (1.26%-98.7%)
Group3 40-49 years	.685 (.417-.953)	76.9%(46.2% -95%)	60% (14.7%-94.7%)	83.3% (51.6%-97.9%)	50% (11.8%-88.2%)
Group4 50-59 years	.76 (.62 -.899)	78.6% (59% -91.7%)	73.3% (44.9%-92.2%)	84.6%(65.1%-95.6%)	64.7% (38.3%-85.8%)
Group4 >60 years	.682(.547-.817)	73.9%(51.6%-89.8%)	62.5% (40.6%-81.2%)	65.4% (44.3%-82.8%)	71.4%(47.8%-88.7%)
Cut off	1.0				
Whole cohort	.725(.641-.808)	71% (58.8%-81.3%)	73.9%(58.9%-85.7%)	80.3%(68.2%-89.4%)	63%(48.7%-75.7%)
Group1 < 30 years	1 (. 1-	100%(15.8%-100%)	100% (2.5%-100%)	100%(15.8%-100%)	100%(2.5%-100%)
Group2 30-39 years	.667(. -1-)	33.3% (.84%-90.6%)	100% (2.5%-100%)	100% (2.5%-100%)	33.3%(.84%-90.6%)
Group3 40-49 years	.785(.555-1)	76.9%(46.2%-95%)	80% (28.4%-99.5%)	90.9%(58.7%-99.8%)	57.1% (18.4%-90.1%)
Group4 50-59 years	.757(.622-.892)	71.4%(51.3%-86.8%)	80% (51.9%-95.7%)	87% (66.4%-97.2%)	60% (36.1%-80.9%)
Group4 >60 years	.681(.545-.817)	69.6% (47.1%-86.8%)	66.7% (44.7%-84.4%)	66.7%(44.7%-84.4%)	69.6%(47.1%-86.8%)

Cut off	1.1				
Whole cohort	.739(.658-.82)	69.6%(57.3%-80.1%)	78.3% (63.6%-89.1%)	82.8%(70.6% -91.4%)	63.2% (49.3%-75.6%)
Group1 < 30 years	1 (. -1-)	100% (15.8%-100%)	100% (2.5%-100%)	100% (15.8%-100%)	100%(2.5%-100%)
Group2 30-39 years	.667(. -1)	33.3%(.84%-90.6%)	100% (2.5%-100%)	100%(2.5%-100%)	33.3%(.84%-90.6%)
Group3 40-49 years	.746 (.511-.982)	69.2% (38.6%-90.9%)	80% (28.4%-99.5%)	90% (55.5%-99.7%)	50% (15.7%-84.3%)
Group4 50-59 years	.757 (.622-.892)	71.4% (51.3% -86.8%)	80% (51.9%-95.7%)	87%(66.4%-97.2%)	60% (36.1%-80.9%)
Group4 >60 years	.723(.592-.853)	69.6% (47.1%-86.8%)	75%(53.3%-90.2%)	72.7% (49.8%-89.3%)	72%(50.6%-87.9%)
Cut off	1.2				
Whole cohort	.736(.658-.813)	62.3% (49.8%-73.7%)	84.8% (71.1%-93.7%)	86% (73.3%-94.2%)	60% (47.1%-72%)
Group1 < 30 years	.75(.- 1)	50% (1.26%-98.7%)	100%(2.5%-100%)	100% (2.5%-100%)	50% (1.26% -98.7%)
Group2 30-39 years	.667(.- 1)	33.3%(.84%-90.6%)	100% (2.5%-100%)	100% (2.5%-100%)	33.3% (.84%-90.6%)
Group3 40-49 years	.808(.67-.945)	61.5% (31.6%-86.1%)	100%(47.8% 100%	100% (63.1%-100%)	50% (18.7%-81.3%)
Group4 50-59 years	.755 (.628-.882)	64.3% (44.1%-81.4%)	86.7% (59.5%-98.3%)	90% (68.3%-98.8%)	56.5% (34.5%-76.8%)
Group4 >60 years	.722(.592-.851)	65.2% (42.7%-83.6%)	79.2% (57.8%-92.9%)	75% (50.9%-91.3%)	70.4%(49.8%-86.2%)
Cut off	1.3				
Whole cohort	.721(.646-.796)	55.1% (42.6%-67.1%)	89.1% (76.4%-96.4%)	88.4% (74.9%-96.1%)	56.9%(44.7%-68.6%)
Group1 < 30 years					
Group2 30-39 years	.667(.- 1)	33.3%(.84%-90.6%)	100%(2.5%-100%)	100% (2.5%-100%)	33.3% (.84%-90.6%)
Group3 40-49 years	.808(.67-.945)	61.5% (31.6%-86.1%)	100%(47.8%-100%)	100%(63.1%-100%)	50% (18.7%-81.3%)
Group4 50-59 years	.735(.62-.849)	53.6% (33.9%-72.5%)	93.3% (68.1%-99.8%)	93.8%(69.8%-99.8%)	51.9%(31.9%-71.3%)
Group 2+3+4					
Group4 >60 years	.721(.594-.848)	60.9% (38.5%-80.3%)	83.3%(62.6%-95.3%)	77.8%(52.4%-93.6%)	69%(49.2%-84.7%)
Cut off	1.4				
Whole cohort	.732(.66-.804)	55.1% (42.6%-67.1%)	91.3% (79.2%-97.6%)	90.5% (77.4%-97.3%)	57.5%(45.4%-69%)

Group1 < 30 years					
Group2 30-39 years	.667(.- 1)	33.3% (.84% - 90.6%)	100%(2.5% - 100%)	100%(2.5%- 100%)	33.3% (.84%- 90.6%)
Group3 40-49 years	.808 (.67 - .945)	61.5% (31.6%- 86.1%)	100%(47.8%- 100%)	100%(63.1%- 100%)	50% (18.7%- 81.3%)
Group4 50-59 years	.735(.62- .849)	53.6% (33.9%- 72.5%)	93.3%(68.1%- 99.8%)	93.8% (69.8% - 99.8%)	51.9% (31.9%- 71.3%)
Group4 >60 years	.742(.62- .864)	60.9% (38.5%- 80.3%)	87.5%(67.6%- 97.3%)	82.4% (56.6%- 96.2%)	70% (50.6%- 85.3%)
Cut off	1.5				
Whole cohort	.721(.652- .791)	50.7% (38.4%- 63%)	93.5% (82.1%- 98.6%)	92.1%(78.6%- 98.3%)	55.8%(44.1%- 67.2%)
Group1 < 30 years					
Group2 30-39 years					
Group3 40-49 years	.769(.628- .91)	53.8%(25.1%- 80.8%)	100%(47.8%- 100%)	100%(59%- 100%)	45.5% (16.7%- 76.6%)
Group4 50-59 years	.735 (.62- .849)	53.6% (33.9%- 72.5%)	93.3%(68.1%- 99.8%)	93.8%(69.8%- 99.8%)	51.9% (31.9%- 71.3%)
Group4 >60 years	.741(.623- .859)	56.5% (34.5%- 76.8%)	91.7% (73%- 99%)	86.7% (59.5%- 98.3%)	68.8% (50% - 83.9%)
Cut off	1.6				
Whole cohort	.692(.623- .761)	44.9%(32.9%- 57.4%)	93.5% (82.1%- 98.6%)	91.2% (76.3%- 98.1%)	53.1% (41.7%- 64.3%)
Group1 < 30 years					
Group2 30-39 years					
Group3 40-49 years	.769(.628- .91)	53.8%(25.1%- 80.8%)	100%(47.8%- 100%)	100%(59% - 100%)	45.5% (16.7%- 76.6%)
Group4 50-59 years	.699(.584- .813)	46.4% (27.5%- 66.1%)	93.3% 68.1% 99.8%	92.9%(66.1%- 99.8%)	48.3%(29.4%- 67.5%)
Group4 >60 years	.697 (.579- .816)	47.8% (26.8%- 69.4%)	91.7%(73%- 99%)	84.6%(54.6%- 98.1%)	64.7%(46.5%- 80.3%)
Cut off	1.7				
Whole cohort	.678(.609- .746)	42% (30.2%- 54.5%)	93.5% (82.1%- 98.6%)	90.6%(75%- 98%)	51.8%(40.6%- 62.9%)
Group1 < 30 years					
Group2 30-39 years					

Group3 40-49 years	.731(.59-.872)	46.2% (19.2%-74.9%)	100%(47.8%-100%)	100%(54.1%-100%)	41.7% (15.2%-72.3%)
Group4 50-59 years	.699(.584-.813)	46.4% (27.5%-66.1%)	93.3%(68.1%-99.8%)	92.9% (66.1%-99.8%)	48.3%(29.4%-67.5%)
Group4 >60 years	.676 (.558-.794)	43.5% (23.2%-65.5%)	91.7%(73%-99%)	83.3%(51.6%-97.9%)	62.9%(44.9%-78.5%)
Cut off	1.8				
Whole cohort	.667(.602-.732)	37.7%(26.3%-50.2%)	95.7%(85.2%-99.5%)	92.9% (76.5%-99.1%)	50.6%(39.6%-61.5%)
Group1 < 30 years					
Group2 30-39 years					
Group3 40-49 years	.692(.555-.83)	38.5%(13.9%-68.4%)	100%(47.8%-100%)	100% (47.8%-100%)	38.5% (13.9%-68.4%)
Group4 50-59 years	.681(.567-.795)	42.9% (24.5%-62.8%)	93.3%(68.1%-99.8%)	92.3% (64% -99.8%)	46.7%(28.3%-65.7%)
Group4 >60 years	.675 (.565-.785)	39.1% (19.7%-61.5%)	95.8% (78.9%-99.9%)	90% (55.5%-99.7%)	62.2%(44.8%-77.5%)
Cut off	1.9				
Whole cohort	.667(.602-.732)	37.7% (26.3%-50.2%)	95.7%(85.2%-99.5%)	92.9% (76.5%-99.1%)	92.9% (76.5%-99.1%)
Group1 < 30 years					
Group2 30-39 years					
Group3 40-49 years	.692 (.555-.83)	38.5% (13.9%-68.4%)	100%(47.8%-100%)	100% (47.8%-100%)	38.5% (13.9%-68.4%)
Group4 50-59 years	.681(.567-.795)	42.9%(24.5%-62.8%)	93.3%(68.1%-99.8%)	92.3%(64%-99.8%)	46.7% (28.3%-65.7%)
Group4 >60 years	.675(.565-.785)	39.1% (19.7%-61.5%)	95.8% (78.9%-99.9%)	90% (55.5%-99.7%)	62.2%(44.8%-77.5%)
Cut off	2.0				
Whole cohort	.652 (.588-.716)	34.8%(23.7%-47.2%)	95.7%(85.2%-99.5%)	92.3% (74.9%-99.1%)	49.4% (38.7%-60.2%)
Group1 < 30 years					
Group2 30-39 years					
Group3 40-49 years	.692(.555-.83)	38.5% (13.9%-68.4%)	100% (47.8%-100%)	100%(47.8%-100%)	38.5%(13.9%-68.4%)
Group4 50-59 years					
Group4 >60 years					

******************************Ringdiff*******************************

Diagnostic accuracy of ringdiff, All cutoff values, (participants with DM).

Age group	ROC	Sensitivity	Specificity	PPV	NPV
Cut off	0.5				
Whole cohort	.675(.581-.769)	74.5% (61%-85.3%)	60.5%(44.4%-75%)	70.7% (57.3%-81.9%)	65% (48.3%-79.4%)
Group1 < 30 years	.75(. -1)	50% (1.26%-98.7%)	100% (2.5%-100%)	100% (2.5%-100%)	50% (1.26%-98.7%)
Group2 30-39 years	.667(.- 1)	33.3% (.84%-90.6%)	100%(2.5%-100%)	100% (2.5%-100%)	33.3%).84%-90.6%)
Group3 40-49 years	.875(.715-1)	75% (34.9%-96.8%)	100% (29.2%-100%)	100%(54.1%-100%)	60% (14.7%-94.7%)
Group4 50-59 years	.685(.538-.832)	80.8%(60.6%-93.4%)	56.3%(29.9%-80.2%)	75%(55.1%-89.3%)	64.3% (35.1%-87.2%)
Group4 >60 years	.648 (.495-.801)	75% (47.6%-92.7%)	54.5% (32.2%-75.6%)	54.5% (32.2%-75.6%)	75%(47.6%-92.7%)
Cut off	0.6				
Whole cohort	.668 (.574-.763	70.9%(57.1%-82.4%)	62.8% (46.7%-77%)	70.9% (57.1%-82.4%)	62.8%(46.7%-77%)
Group1 < 30 years	.75(. -1)	50%(1.26%-98.7%)	100% (2.5%-100%)	100%(2.5%-100%)	50% (1.26%-98.7%)
Group2 30-39 years	.667(.- 1)	33.3%(.84%-90.6%)	100% (2.5% -100%)	100%(2.5%-100%)	33.3%(.84%-90.6%)
Group3 40-49 years	.875(.715-1)	75% (34.9%-96.8%)	100% (29.2%-100%)	100% (54.1%-100%)	60% (14.7%-94.7%)
Group4 50-59 years	.647(.494-.799)	73.1% (52.2%-88.4%)	56.3% (29.9%-80.2%)	73.1%(52.2%-88.4%)	56.3%(29.9%-80.2%)
Group4 >60 years	.67 (.519-.822)	75%(47.6%-92.7%)	59.1%(36.4%-79.3%)	57.1%(34%-78.2%)	76.5% (50.1%-93.2%)
Cut off	0.7				
Whole cohort	.644(.547-.74)	63.6%(49.6%-76.2%)	65.1% (49.1%-79%)	70%(55.4%-82.1%)	58.3%(43.2%-72.4%)
Group1 < 30 years	.75(.- 1)	50%(1.26%-98.7%)	100%(2.5%-100%)	100% (2.5%-100%)	50%(1.26%-98.7%)
Group2 30-39 years					
Group3 40-49 years	.75(.565-.935)	50% (15.7%-84.3%)	100% (29.2%-100%)	100%(39.8%-100%)	42.9%(9.9%-81.6%)
Group4 50-59 years	.627(.473-.782)	69.2% (48.2%-85.7%)	56.3% (29.9%-80.2%)	72%(50.6%-87.9%)	52.9% (27.8%-77%)

Group4 >60 years	.693 (.543 - .843)	75% (47.6%- 92.7%)	63.6%(40.7%- 82.8%)	60% (36.1% - 80.9%)	77.8% (52.4%- 93.6%)
Cut off	0.8				
Whole cohort	.637(.541- .734)	60%(45.9%- 73%)	67.4% (51.5%- 80.9%)	70.2% (55.1%- 82.7%)	56.9%(42.2%- 70.7%)
Group1 < 30 years	.75(.- 1)	50% (1.26%- 98.7%)	100%(2.5%- 100%)	100%(2.5%- 100%)	50% (1.26%- 98.7%)
Group2 30-39 years					
Group3 40-49 years	.688(.508- .867)	37.5%(8.52%- 75.5%)	100% (29.2%- 100%)	100%(29.2%- 100%)	37.5%(8.52%- 75.5%)
Group4 50-59 years	.639 (.485- .793)	65.4% (44.3%- 82.8%)	62.5%(35.4%- 84.8%)	73.9% (51.6%- 89.8%)	52.6%(28.9%- 75.6%)
Group4 >60 years	.693(.543- .843)	75%(47.6%- 92.7%)	63.6% (40.7%- 82.8%)	60% (36.1%- 80.9%)	77.8% (52.4%- 93.6%)
Cut off	0.9				
Whole cohort	.642(.548- .737)	56.4% (42.3%- 69.7%)	72.1% (56.3%- 84.7%)	72.1% (56.3%- 84.7%)	56.4%(42.3%- 69.7%)
Group1 < 30 years	.75(.- 1)	50% (1.26%- 98.7%)	100% (2.5%- 100%)	100% (2.5%- 100%)	50%(1.26%- 98.7%)
Group2 30-39 years					
Group3 40-49 years	.688(.508- .867)	37.5%(8.52%- 75.5%)	100% (29.2%- 100%)	100%(29.2%- 100%)	37.5%(8.52%- 75.5%)
Group4 50-59 years	.651(.5- .803)	61.5%(40.6%- 79.8%)	68.8% (41.3%- 89%)	76.2% (52.8%- 91.8%)	52.4% (29.8%- 74.3%)
Group4 >60 years	.685 (.531- .839)	68.8% (41.3%- 89%)	68.2%(45.1%- 86.1%)	61.1%(35.7%- 82.7%)	75%(50.9%- 91.3%)
Cut off	1.0				
Whole cohort	.645 (.551- .738)	54.5% (40.6%- 68%)	74.4% (58.8%- 86.5%)	73.2%(57.1%- 85.8%)	56.1% (42.4%- 69.3%)
Group1 < 30 years	.75(.- 1)	50% (1.26%- 98.7%)	100%(2.5%- 100%)	100%(2.5%- 100%)	50%(1.26%- 98.7%)
Group2 30-39 years					
Group3 40-49 years	.688(.508- .867)	37.5% (8.52%- 75.5%)	100%(29.2%- 100%)	100%(29.2%- 100%)	37.5% (8.52%- 75.5%)
Group4 50-59 years	.663(.517- .81)	57.7%(36.9%- 76.6%)	75% (47.6%- 92.7%)	78.9% (54.4%- 93.9%)	52.2%(30.6%- 73.2%)
Group4 >60 years	.685(.531- .839)	68.8%(41.3%- 89%)	68.2% (45.1%- 86.1%)	61.1% (35.7%- 82.7%)	75% (50.9%- 91.3%)
Cut off	1.1				

Whole cohort	.641 (.55-.732)	49.1% (35.4%-62.9%)	79.1%(64%-90%)	75% (57.8%-87.9%)	54.8% (41.7%-67.5%)
Group1 < 30 years	.75(.- 1)	50% (1.26%-98.7%)	100%(2.5%-100%)	100%(2.5%-100%)	50% (1.26%-98.7%)
Group2 30-39 years					
Group3 40-49 years	.688 (.508-.867)	37.5%(8.52%-75.5%)	100% (29.2%-100%)	100%(29.2%-100%)	37.5%(8.52%-75.5%)
Group4 50-59 years	.675 (.537-.814)	53.8%(33.4%-73.4%)	81.3%(54.4%-96%)	82.4% (56.6%-96.2%)	52% (31.3%-72.2%)
Group4 >60 years	.645 (.487-.802)	56.3%(29.9%-80.2%)	72.7% (49.8%-89.3%)	60% (32.3%-83.7%)	69.6%(47.1%-86.8%)
Cut off	1.2				
Whole cohort	.667(.582-.751)	47.3%(33.7%-61.2%)	86% (72.1%-94.7%)	81.3%(63.6%-92.8%)	56.1%(43.3%-68.3%)
Group1 < 30 years	.75(. -1)	50%(1.26%-98.7%)	100% (2.5%-100%)	100% (2.5%-100%)	50%(1.26%-98.7%)
Group2 30-39 years					
Group3 40-49 years	.688 (.508-.867)	37.5%(8.52%-75.5%)	100% (29.2%-100%)	100% (29.2%-100%)	37.5%(8.52%-75.5%)
Group4 50-59 years	.675 (.537-.814)	53.8%(33.4%-73.4%)	81.3%(54.4%-96%)	82.4% (56.6%-96.2%)	52% (31.3%-72.2%)
Group4 >60 years	.682(.536-.828)	50%(24.7%-75.3%)	86.4%(65.1%-97.1%)	72.7% (39%-94%)	70.4%(49.8%-86.2%)
Cut off	1.3				
Whole cohort	.669(.587-.751)	45.5%(32%-59.4%)	88.4% (74.9%-96.1%)	83.3%(65.3%-94.4%)	55.9% (43.3%-67.9%)
Group1 < 30 years	.75(.- 1)	50% (1.26%-98.7%)	100%(2.5%-100%)	100(2.5%-100%)	50%(1.26%-98.7%)
Group2 30-39 years					
Group3 40-49 years	.688(.508-.867)	37.5%(8.52%-75.5%)	100% (29.2%-100%)	100% (29.2%-100%)	37.5% (8.52%-75.5%)
Group4 50-59 years	.675(.537-.814)	53.8% (33.4%-73.4%)	81.3%(54.4%-96%)	82.4% (56.6%-96.2%)	52% (31.3%-72.2%)
Group4 >60 years	.673 (.534-.813)	43.8% (19.8%-70.1%)	90.9% (70.8%-98.9%)	77.8% (40%-97.2%)	69%(49.2%-84.7%)
Cut off	1.4				
Whole cohort	.642(.561-.723)	40% (27% -54.1%)	88.4%(74.9%-96.1%)	81.5%(61.9%-93.7%)	53.5%(41.3%-65.5%)
Group1 < 30 years	.75(.- 1)	50%(1.26%-98.7%)	100% (2.5%-100%)	100%(2.5%-100%)	50%(1.26%-98.7%)

Group2 30-39 years					
Group3 40-49 years	.688 (.508-.867)	37.5%(8.52%-75.5%)	100%(29.2%-100%)	100%(29.2%-100%)	37.5% (8.52%-75.5%)
Group4 50-59 years	.637 (.498-.776)	46.2% (26.6%-66.6%)	81.3% (54.4%-96%)	80% (51.9%-95.7%)	48.1%(28.7%-68.1%)
Group4 >60 years	.642(.505-.779)	37.5%(15.2%-64.6%)	90.9% (70.8%-98.9%)	75% (34.9%-96.8%)	66.7%(47.2%-82.7%)
Cut off	1.5				
Whole cohort	.629 (.555-.702)	32.7%(20.7%-46.7%)	93% (80.9%-98.5%)	85.7% (63.7%-97%)	51.9% (40.3%-63.5%)
Group1 < 30 years					
Group2 30-39 years					
Group3 40-49 years	688(.508-.867)	37.5% (8.52%-75.5%)	100%(29.2%-100%)	100% (29.2%-100%)	37.5%(8.52%-75.5%)
Group4 50-59 years	.649(.521-.777)	42.3% (23.4%-63.1%)	87.5%(61.7%-98.4%)	84.6%(54.6%-98.1%)	48.3% (29.4%-67.5%)
Group4 >60 years	.602(.484-.721)	25% (7.27%-52.4%)	95.5% (77.2%-99.9%)	80% (28.4%-99.5%)	63.6% (45.1%-79.6%)
Cut off	1.6				
Whole cohort	.629 (.555-.702)	32.7%(20.7%-46.7%)	93% (80.9%-98.5%)	85.7%(63.7%-97%)	51.9%(40.3%-63.5%)
Group1 < 30 years					
Group2 30-39 years					
Group3 40-49 years	.688(.508-.867)	37.5% (8.52%-75.5%)	100% (29.2%-100%)	100% (29.2%-100%)	37.5%(8.52%-75.5%)
Group4 50-59 years	.649(.521-.777)	42.3%(23.4%-63.1%)	87.5% (61.7%-98.4%)	84.6%(54.6%-98.1%)	48.3%(29.4%-67.5%)
Group4 >60 years	.602 (.484-.721)	25% (7.27%-52.4%)	95.5%(77.2% -99.9%)	80%(28.4%-99.5%)	63.6% (45.1%-79.6%)
Cut off	1.7				
Whole cohort	.631 (.562-.701)	30.9% (19.1%-44.8%)	95.3%(84.2%-99.4%)	89.5% (66.9%-98.7%)	51.9%(40.4%-63.3%)
Group1 < 30 years					
Group2 30-39 years					
Group3 40-49 years	.688 (.508-.867)	37.5% (8.52%-75.5%)	100% (29.2%-100%)	100% (29.2%-100%)	37.5%(8.52%-75.5%)
Group4 50-59 years	.68 (.566-.795)	42.3% (23.4%-63.1%)	93.8%(69.8%-99.8%)	91.7%(61.5%-99.8%)	50%(31.3%-68.7%)
Group4 >60 years					

********************************CSI*********************************

Diagnostic accuracy of combined sensory index, All cutoff values, (participants with DM).

Age group	ROC	Sensitivity	Specificity	PPV	NPV
Cut off	1.0				
Whole cohort	.663(.585-.74)	95.4% (87.1%-99%)	37.2%(23%-53.3%	69.7% (59%-79%)	84.2% (60.4%-96.6%)
Group1 < 30 years	1(.- 1)	100% (15.8%-100%)	100%(2.5%-100%)	100% (15.8%-100%)	100% (2.5%-100%)
Group2 30-39 years	.833(.- 1)	66.7% (9.43%-99.2%)	100% (2.5%-100%)	100%(15.8%-100%)	50% (1.26%-98.7%)
Group3 40-49 years	.583(.325-.842)	91.7% (61.5% 99.8%	25% (.631%-80.6%)	78.6% (49.2%-95.3%)	50% (1.26%-98.7%)
Group4 50-59 years	.635 (.499-.771)	96.2%(80.4%-99.9%)	30.8%(9.09%-61.4%)	73.5% (55.6%-87.1%)	80% (28.4%-99.5%)
Group4 >60 years	.688 (.589-.786)	100%(84.6%-100%)	37.5%(18.8%-59.4%)	59.5% (42.1%-75.2%)	100% (66.4%-100%)
Cut off	1.1				
Whole cohort	.69(.61 .-771)	93.8% (85%-98.3%)	44.2%(29.1%-60.1%)	71.8%(61%-81%)	82.6%(61.2%-95%)
Group1 < 30 years	1(.- 1)	100% (15.8%-100%)	100% (2.5%-100%)	100% (15.8%-100%)	100%(2.5%-100%)
Group2 30-39 years	.833(.- 1)	66.7% (9.43%-99.2%)	100% (2.5%-100%)	100% (15.8%-100%)	50% (1.26%-98.7%)
Group3 40-49 years	.708 (.414-1)	91.7%(61.5%-99.8%)	50%(6.76%-93.2%)	84.6%(54.6%-98.1%)	66.7% (9.43%-99.2%)
Group4 50-59 years	.654(.507-.801)	92.3% (74.9%-99.1%)	38.5% (13.9%-68.4%)	75%(56.6%-88.5%)	71.4% (29%-96.3%)
Group4 >60 years	.708 (.608-.809)	100% (84.6%-100%)	41.7% (22.1%-63.4%)	61.1%(43.5%-76.9%)	100%(69.2%-100%)
Cut off	1.2				
Whole cohort	.717(.635-.8)	92.3%(83%-97.5%)	51.2%(35.5%-66.7%)	74.1% (63.1%-83.2%)	81.5%(61.9%-93.7%)
Group1 < 30 years	1(.- 1)	100% (15.8%-100)	100%(2.5%-100%)	100%(15.8%-100%)	100%(2.5%-100%)
Group2 30-39 years	.833 (.- 1)	66.7% (9.43%-99.2%)	100% (2.5%-100%)	100% (15.8%-100%)	50%(1.26%-98.7%)
Group3 40-49 years	.833(.575-1)	91.7%(61.5%-99.8%)	75% (19.4%-99.4%)	91.7%(61.5%-99.8%)	75% (19.4%-99.4%)

Group4 50-59 years	.692(.542-.843)	92.3%(74.9%-99.1%)	46.2% (19.2%-74.9%)	77.4% (58.9%-90.4%)	75% (34.9%-96.8%)
Group4 >60 years	.706(.595-.818)	95.5%(77.2%-99.9%)	45.8% (25.6%-67.2%)	61.8%(43.6%-77.8%)	91.7% (61.5%-99.8%)
Cut off	1.3				
Whole cohort	.741 (.659-.822)	92.3% (83%-97.5%)	55.8%(39.9%-70.9%)	75.9%(65%-84.9%)	82.8% (64.2%-94.2%)
Group1 < 30 years	1(.- 1)	100% (15.8%-100%)	100% (2.5%-100%)	100% (15.8%-100%)	100% (2.5%-100%)
Group2 30-39 years	.833(.- 1)	66.7%(9.43%-99.2%)	100% (2.5%-100%)	100% (15.8%-100%)	50% (1.26%-98.7%)
Group3 40-49 years	.958(.877-1)	91.7%(61.5%-99.8%)	100% (39.8%-100%)	100% (71.5%-100%)	80% (28.4%-99.5%)
Group4 50-59 years	.692(.542-.843)	92.3%(74.9%-99.1%)	46.2% (19.2%-74.9%)	77.4%(58.9%-90.4%)	75% 34.9% 96.8%
Group4 >60 years	.727 (.616-.839)	95.5% (77.2%-99.9%)	50% (29.1%-70.9%)	63.6% (45.1%-79.6%)	92.3%(64%-99.8%)
Cut off	1.4				
Whole cohort	.737(.653-.821)	89.2% (79.1%-95.6%)	58.1% (42.1%-73%)	76.3% (65.2%-85.3%)	78.1%(60%-90.7%)
Group1 < 30 years	1(.- 1)	100% (15.8%-100%)	100%(2.5%-100%)	100%(15.8%-100%)	100% (2.5%-100%)
Group2 30-39 years	.833(.- 1)	66.7%(9.43%-99.2%)	100%(2.5%-100%)	100% (15.8%-100%)	50% (1.26%-98.7%)
Group3 40-49 years	.958(.877-1)	91.7% (61.5%-99.8%)	100% (39.8%-100%)	100% (71.5%-100%)	80% (28.4%-99.5%)
Group4 50-59 years	.712 (.557-.866)	88.5% (69.8%-97.6%)	53.8% (25.1%-80.8%)	79.3% (60.3%-92%)	70% (34.8%-93.3%)
Group4 >60 years	.705 (.585-.824)	90.9% (70.8%-98.9%)	50% (29.1%-70.9%)	62.5% (43.7%-78.9%)	85.7%(57.2%-98.2%)
Cut off	1.5				
Whole cohort	.737 (.653-.821)	89.2% (79.1%-95.6%)	58.1% (42.1%-73%)	76.3% (65.2%-85.3%)	78.1% (60%-90.7%)
Group1 < 30 years	1(. -1)	100% (15.8%-100%)	100%(2.5%-100%)	100% (15.8%-100%)	100% (2.5%-100%)
Group2 30-39 years	.833(.- 1)	66.7% (9.43%-99.2%)	100% (2.5%-100%)	100% (15.8%-100%)	50% (1.26%-98.7%)
Group3 40-49 years	.958 (.877-1)	91.7% (61.5%-99.8%)	100% (39.8%-100%)	100% (71.5%-100%)	80% (28.4%-99.5%)
Group4 50-59 years	.712 (.557-.866)	88.5% (69.8%-97.6%)	53.8%(25.1%-80.8%)	79.3%(60.3%-92%)	70% (34.8%-93.3%)
Group4 >60 years	.705 (.585-.824)	90.9% (70.8%-98.9%)	50% (29.1%-70.9%)	62.5% (43.7%-78.9%)	85.7%(57.2%-98.2%)

Cut off	1.6				
Whole cohort	.714 (.627-.8)	84.6% (73.5%-92.4%)	58.1% (42.1%-73%)	75.3% (63.9%-84.7%)	71.4% (53.7%-85.4%)
Group1 < 30 years	.75 (. -1)	50% (1.26%-98.7%)	100% (2.5%-100%)	100% (2.5%-100%)	50%(1.26%-98.7%)
Group2 30-39 years	.833(.- 1)	66.7%(9.43%-99.2%)	100% (2.5%-100%)	100% (15.8%-100%)	50% (1.26%-98.7%)
Group3 40-49 years	.917 (.807-1)	83.3%(51.6%-97.9%)	100% (39.8%-100%)	100% (69.2%-100%)	66.7% (22.3%-95.7%)
Group4 50-59 years	.712 (.557-.866)	88.5% (69.8%-97.6%)	53.8% (25.1%-80.8%)	79.3% (60.3%-92%)	70%(34.8%-93.3%)
Group4 >60 years	.682 (.556-.808)	86.4% (65.1% -97.1%)	50% (29.1%-70.9%)	61.3%(42.2%-78.2%)	80% (51.9%-95.7%)
Cut off	1.7				
Whole cohort	.741(.656-.826)	83.1% (71.7%-91.2%)	65.1% (49.1%-79%)	78.3%(66.7%-87.3%)	71.8% (55.1%-85%)
Group1 < 30 years	.75(.- 1)	50% (1.26%-98.7%)	100% (2.5%-100%)	100% (2.5% -100%)	50%(1.26%-98.7%)
Group2 30-39 years	.833(. -1)	66.7% (9.43%-99.2%)	100% (2.5%-100%)	100%(15.8%-100%)	50% (1.26%-98.7%)
Group3 40-49 years	.917 (.807-1)	83.3% (51.6%-97.9%)	100% (39.8%-100%)	100%(69.2%-100%)	66.7% (22.3%-95.7%)
Group4 50-59 years	.692(.535-.85)	84.6% (65.1%-95.6%)	53.8% (25.1%-80.8%)	78.6% (59%-91.7%)	63.6% (30.8%-89.1%)
Group4 >60 years	.744(.621-.867)	86.4% (65.1%-97.1%)	62.5% (40.6%-81.2%)	67.9% (47.6%-84.1%)	83.3(58.6%-96.4%)
Cut off	2.0				
Whole cohort	.737(.651-.823)	80%(68.2%-88.9%)	67.4% (51.5%-80.9%)	78.8%(67%-87.9%)	69% (52.9%-82.4%)
Group1 < 30 years	.75(.- 1)	50% (1.26%-98.7%)	100%(2.5%-100%)	100% (2.5%-100%)	50%(1.26%-98.7%)
Group2 30-39 years	.667(.- 1)	33.3%(.84%-90.6%)	100%(2.5%-100%)	100%(2.5%-100%)	33.3%(.84%-90.6%)
Group3 40-49 years	.917(.807-1)	83.3% (51.6%-97.9%)	100% (39.8%-100%)	100%(69.2%-100%)	66.7% (22.3%-95.7%)
Group4 50-59 years	.731(.576-.886)	84.6%(65.1%-95.6%)	61.5% (31.6%-86.1%)	81.5%(61.9%-93.7%)	66.7% (34.9%-90.1%)
Group4 >60 years	.722 (.593-.85)	81.8% (59.7%-94.8%)	62.5%(40.6%-81.2%)	66.7% (46%-83.5%)	78.9% (54.4%-93.9%)
Cut off	2.5				
Whole cohort	.711 (.623-.798)	67.7% (54.9%-78.8%)	74.4% (58.8%-86.5%)	80% (67%-89.6%)	60.4% (46%-73.5%)
Group1 < 30 years	.75(.- 1)	50%(1.26% -98.7%)	100%(2.5%-100%)	100% (2.5%-100%)	50% (1.26%-98.7%)

Group2 30-39 years					
Group3 40-49 years	.833(.694-.973)	66.7% (34.9%-90.1%)	100% (39.8%-100%)	100%(63.1%-100%)	50% (15.7%-84.3%)
Group4 50-59 years	.731 (.576-.885)	76.9% (56.4%-91%)	69.2% (38.6%-90.9%)	83.3% (62.6%-95.3%)	60% (32.3%-83.7%)
Group4 >60 years	.695 (.559-.831)	68.2% (45.1%-86.1%)	70.8%(48.9%-87.4%)	68.2% (45.1%-86.1%)	70.8% (48.9%-87.4%)
Cut off	3.0				
Whole cohort	.719(.637-.801)	60% (47.1%- 72%)	83.7% (69.3%-93.2%)	84.8% (71.1%-93.7%)	58.1%(44.8%-70.5%)
Group1 < 30 years	.75(. -1)	50% (1.26%-98.7%)	100%(2.5%-100%)	100%(2.5%-100%)	50% (1.26%-98.7%)
Group2 30-39 years					
Group3 40-49 years	.792 (.646-.937)	58.3%(27.7%-84.8%)	100%(39.8%-100%)	100% (59%-100%)	44.4% (13.7%-78.8%)
Group4 50-59 years	.788(.669-.908)	65.4% (44.3%-82.8%)	92.3%(64%-99.8%)	94.4% (72.7%-99.9%)	57.1%(34%-78.2%)
Group4 >60 years	.693 (.557-.829)	63.6%(40.7%-82.8%)	75% (53.3%-90.2%)	70% (45.7%-88.1%)	69.2%(48.2%-85.7%)

Cut off	3.5				
Whole cohort	.707(.632 -.783)	50.8% (38.1%-63.4%)	90.7%(77.9%-97.4%)	89.2% (74.6%-97%)	54.9%(42.7%-66.8%)
Group1 < 30 years					
Group2 30-39 years					
Group3 40-49 years					
Group4 50-59 years					
Group4 >60 years	.71 (.584-.836)	54.5% (32.2%-75.6%)	87.5% (67.6%-97.3%)	80%(51.9%-95.7%)	67.7% (48.6%-83.3%)
