# Supplementary material for: Values and Diagnostic Accuracy of Electrodiagnostic Findings in Carpal Tunnel Syndrome Based on Age, Gender, and Diabetes
Source: Diagnostics (Basel). 2024 Jun 28;14(13):1381. doi: 10.3390/diagnostics14131381 (PMC11240809; doi:10.3390/diagnostics14131381)
Supplement: Supplementary file 1 [file diagnostics-14-01381-s001.zip › Table S7 Diagnostic accuracy of median latency at Digit II and comparative latency studies (COLSs), all cutoff values (female participants);.pdf]

Table S7 Diagnostic accuracy of median latency at Digit II, All cutoff values, (female participants)

Age group	ROC	Sensitivity	Specificity	PPV	NPV
Cut off	3.5 (ms)				
Whole cohort	.755 (.711-.8)	67.6% (61.8%-73%)	83.5% (75.2%-89.9%)	91.4% (86.8%-94.8%)	49.7% (42.3%-57.2%)
Group1 < 30 years	.658 (.551-.765)	31.6% (12.6%-56.6%)	100% (85.2%-100%)	100% (54.1%-100%)	63.9% (46.2%-79.2%)
Group2 30-39 years	.639 (.52-.758)	45.5% (30.4%-61.2%)	82.4% (56.6%-96.2%)	87% (66.4%-97.2%)	36.8% (21.8%-54%)
Group3 40-49 years	.799 (.733-.865)	63.5% (51.5%-74.4%)	96.3% (81%-99.9%)	97.9% (88.9%-99.9%)	49.1% (35.1%-63.2%)
Group4 50-59 years	.74 (.643-.836)	77.6% (68.5%-85.1%)	70.4% (49.8%-86.2%)	91.2% (83.4%-96.1%)	44.2% (29.1%-60.1%)
Group4 >60 years	.75 (.613-.887)	90% (76.3%-97.2%)	60% (32.3%-83.7%)	85.7% (71.5%-94.6%)	69.2% (38.6%-90.9%)
Cut off	3.6 (ms)				
Whole cohort	.721 (.677-.765)	58.8% (52.8%-64.6%)	85.3% (77.3%-91.4%)	91.3% (86.2%-94.9%)	44.3% (37.5%-51.3%)
Group1 < 30 years	.605 (.511-.699)	21.1% (6.05%-45.6%)	100% (85.2%-100%)	100% (39.8%-100%)	60.5% (43.4%-76%)
Group2 30-39 years	.657 (.549-.765)	43.2% (28.3%-59%)	88.2% (63.6%-98.5%)	90.5% (69.6%-98.8%)	37.5% (22.7%-54.2%)
Group3 40-49 years	.759 (.691-.826)	55.4% (43.4%-67%)	96.3% (81%-99.9%)	97.6% (87.4%-99.9%)	44.1% (31.2%-57.6%)
Group4 50-59 years	.693 (.597-.789)	64.5% (54.6%-73.5%)	74.1% (53.7%-88.9%)	90.8% (81.9%-96.2%)	34.5% (22.5%-48.1%)
Group4 >60 years	.725 (.585-.865)	85% (70.2%-94.3%)	60% (32.3%-83.7%)	85% (70.2%-94.3%)	60% (32.3%-83.7%)
Cut off	3.7 (ms)				
Whole cohort	.72 (.678-.762)	56% (50%-61.8%)	88.1% (80.5%-93.5%)	92.4% (87.4%-95.9%)	43.4% (36.8%-50.3%)
Group1 < 30 years	.605 (.511-.699)	21.1% (6.05%-45.6%)	100% (85.2%-100%)	100% (39.8%-100%)	60.5% (43.4%-76%)
Group2 30-39 years	.634 (.527-.742)	38.6% (24.4%-54.5%)	88.2% (63.6%-98.5%)	89.5% (66.9%-98.7%)	35.7% (21.6%-52%)
Group3 40-49 years	.731 (.664-.799)	50% (38.1%-61.9%)	96.3% (81%-99.9%)	97.4% (86.2%-99.9%)	41.3% (29%-54.4%)
Group4 50-59 years	.744 (.661-.826)	63.6% (53.7%-72.6%)	85.2% (66.3%-95.8%)	94.4% (86.4%-98.5%)	37.1% (25.2%-50.3%)

Group4 >60 years	.712 (.571-.854)	82.5% (67.2%-92.7%)	60% (32.3%-83.7%)	84.6% (69.5%-94.1%)	56.3% (29.9%-80.2%)
Cut off	3.9 (ms)				
Whole cohort	.7 (.659-.742)	51.1% (45.1%-57%)	89% (81.6%-94.2%)	92.4% (87%-96%)	41.1% (34.8%-47.7%)
Group1 < 30 years	.605 (.511-.699)	21.1% (6.05%-45.6%)	100% (85.2%-100%)	100% (39.8%-100%)	60.5% (43.4%-76%)
Group2 30-39 years	.623 (.516-.73)	36.4% (22.4%-52.2%)	88.2% (63.6%-98.5%)	88.9% (65.3%-98.6%)	34.9% (21%-50.9%)
Group3 40-49 years	.711 (.644-.779)	45.9% (34.3%-57.9%)	96.3% (81%-99.9%)	97.1% (85.1%-99.9%)	39.4% (27.6%-52.2%)
Group4 50-59 years	.734 (.658-.811)	57.9% (48%-67.4%)	88.9% (70.8%-97.6%)	95.4% (87.1%-99%)	34.8% (23.7%-47.2%)
Group4 >60 years	.663 (.516-.809)	72.5% (56.1%-85.4%)	60% (32.3%-83.7%)	82.9% (66.4%-93.4%)	45% (23.1%-68.5%)
Cut off	4.0				
Whole cohort	.695 (.658-.732)	45.4% (39.5%-51.4%)	93.6% (87.2%-97.4%)	94.9% (89.7%-97.9%)	39.7% (33.7%-46%)
Group1 < 30 years	.605 (.511-.699)	21.1% (6.05%-45.6%)	100% (85.2%-100%)	100% (39.8%-100%)	60.5% (43.4%-76%)
Group2 30-39 years	.607 (.519-.695)	27.3% (15%-42.8%)	94.1% (71.3%-99.9%)	92.3% (64%-99.8%)	33.3% (20.4%-48.4%)
Group3 40-49 years	.703 (.646-.759)	40.5% (29.3%-52.6%)	100% (87.2%-100%)	100% (88.4%-100%)	38% (26.8%-50.3%)
Group4 50-59 years	.725 (.655-.794)	52.3% (42.5%-62.1%)	92.6% (75.7%-99.1%)	96.6% (88.1%-99.6%)	32.9% (22.5%-44.6%)
Group4 >60 years	.704 (.567-.841)	67.5% (50.9%-81.4%)	73.3% (44.9%-92.2%)	87.1% (70.2%-96.4%)	45.8% (25.6%-67.2%)
Cut off	4.1				
Whole cohort	.685 (.65-.72)	41.5% (35.8%-47.5%)	95.4% (89.6%-98.5%)	95.9% (90.8%-98.7%)	38.5% (32.7%-44.6%)
Group1 < 30 years	.553 (.482-.624)	10.5% (1.3%-33.1%)	100% (85.2%-100%)	100% (15.8%-100%)	57.5% (40.9%-73%)
Group2 30-39 years	.607 (.519-.695)	27.3% (15%-42.8%)	94.1% (71.3%-99.9%)	92.3% (64%-99.8%)	33.3% (20.4%-48.4%)
Group3 40-49 years	.676 (.621-.73)	35.1% (24.4%-47.1%)	100% (87.2%-100%)	100% (86.8%-100%)	36% (25.2%-47.9%)
Group4 50-59 years	.729 (.669-.789)	49.5% (39.7%-59.4%)	96.3% (81%-99.9%)	98.1% (90.1%-100%)	32.5% (22.4%-43.9%)
Group4 >60 years	.712 (.583-.842)	62.5% (45.8%-77.3%)	80% (51.9%-95.7%)	89.3% (71.8%-97.7%)	44.4% (25.5%-64.7%)
Cut off	4.2				

Whole cohort	.676 (.641-.711)	39.8% (34.1%-45.7%)	95.4% (89.6%-98.5%)	95.8% (90.4%-98.6%)	37.8% (32.1%-43.8%)
Group1 < 30 years	.553 (.482-.624)	10.5% (1.3%-33.1%)	100% (85.2%-100%)	100% (15.8%-100%)	57.5% (40.9%-73%)
Group2 30-39 years	.607 (.519-.695)	27.3% (15%-42.8%)	94.1% (71.3%-99.9%)	92.3% (64%-99.8%)	33.3% (20.4%-48.4%)
Group3 40-49 years	.676 (.621-.73)	35.1% (24.4%-47.1%)	100% (87.2%-100%)	100% (86.8%-100%)	36% (25.2%-47.9%)
Group4 50-59 years	.715 (.655-.775)	46.7% (37%-56.6%)	96.3% (81%-99.9%)	98% (89.6%-100%)	31.3% (21.6%-42.4%)
Group4 >60 years	.687 (.557-.818)	57.5% (40.9%-73%)	80% (51.9%-95.7%)	88.5% (69.8%-97.6%)	41.4% (23.5%-61.1%)
Cut off	4.3				
Whole cohort	.674 (.643-.705)	36.6% (31%-42.5%)	98.2% (93.5%-99.8%)	98.1% (93.4%-99.8%)	37.3% (31.7%-43.2%)
Group1 < 30 years	.553 (.482-.624)	10.5% (1.3%-33.1%)	100% (85.2%-100%)	100% (15.8%-100%)	57.5% (40.9%-73%)
Group2 30-39 years	.625 (.56-.69)	25% (13.2%-40.3%)	100% (80.5%-100%)	100% (71.5%-100%)	34% (21.2%-48.8%)
Group3 40-49 years	.655 (.602-.708)	31.1% (20.8%-42.9%)	100% (87.2%-100%)	100% (85.2%-100%)	34.6% (24.2%-46.2%)
Group4 50-59 years	.692 (.632-.751)	42.1% (32.6%-52%)	96.3% (81%-99.9%)	97.8% (88.5%-99.9%)	29.5% (20.3%-40.2%)
Group4 >60 years	.754 (.653-.856)	57.5% (40.9%-73%)	93.3% (68.1%-99.8%)	95.8% (78.9%-99.9%)	45.2% (27.3%-64%)
Cut off	4.4				
Whole cohort	.656 (.626-.687)	33.1% (27.7%-38.9%)	98.2% (93.5%-99.8%)	97.9% (92.7%-99.7%)	36% (30.6%-41.8%)
Group1 < 30 years	.526 (.475-.578)	5.26% (.133%-26%)	100% (85.2%-100%)	100% (2.5%-100%)	56.1% (39.7%-71.5%)
Group2 30-39 years	.614 (.551-.676)	22.7% (11.5%-37.8%)	100% (80.5%-100%)	100% (69.2%-100%)	33.3% (20.8%-47.9%)
Group3 40-49 years	.635 (.584-.686)	27% (17.4%-38.6%)	100% (87.2%-100%)	100% (83.2%-100%)	33.3% (23.2%-44.7%)
Group4 50-59 years	.682 (.623-.742)	40.2% (30.8%-50.1%)	96.3% (81%-99.9%)	97.7% (88%-99.9%)	28.9% (19.8%-39.4%)
Group4 >60 years	.717 (.615-.819)	50% (33.8%-66.2%)	93.3% (68.1%-99.8%)	95.2% (76.2%-99.9%)	41.2% (24.6%-59.3%)
Cut off	4.5				
Whole cohort	.64 (.611-.67)	29.9% (24.7%-35.6%)	98.2% (93.5%-99.8%)	97.7% (91.9%-99.7%)	35% (29.6%-40.6%)
Group1 < 30 years					

Group2 30-39 years	.602 (.542-.663)	20.5% (9.8%-35.3%)	100% (80.5%-100%)	100% (66.4%-100%)	32.7% (20.3%-47.1%)
Group3 40-49 years	.635 (.584-.686)	27% (17.4%-38.6%)	100% (87.2%-100%)	100% (83.2%-100%)	33.3% (23.2%-44.7%)
Group4 50-59 years	.659 (.601-.717)	35.5% (26.5%-45.4%)	96.3% (81%-99.9%)	97.4% (86.5%-99.9%)	27.4% (18.7%-37.5%)
Group4 >60 years	.692 (.59-.793)	45% (29.3%-61.5%)	93.3% (68.1%-99.8%)	94.7% (74%-99.9%)	38.9% (23.1%-56.5%)

******************************Palmdiff******************************

Diagnostic accuracy of palmdiff, All cutoff values, (female participants).

Age group	ROC	Sensitivity	Specificity	PPV	NPV
Cut off	0.3				
Whole cohort	.737(.685-.789)	77.4%(71.7%-82.4%)	70% (60%-78.8%)	86.7% (81.5%-90.8%)	55.1% 46% 63.9%
Group1 < 30 years	.635(.493-.777)	44.4% (21.5%-69.2%)	82.6% (61.2%-95%)	66.7%(34.9%-90.1%)	65.5% (45.7%-82.1%)
Group2 40-49 years	.644 (.504-.784)	60% (43.3%-75.1%)	68.8% (41.3%-89%)	82.8% (64.2%-94.2%)	40.7% (22.4%-61.2%)
Group3 50-59 years	.842(.765-.919)	77.1% (65.6%-86.3%)	91.3%(72%-98.9%)	96.4% (87.7%-99.6%)	56.8% (39.5%-- 72.9%)
Group4 50-59 years	.751 (.647-.856)	87.8% (79.2%-93.7%)	62.5% (40.6%-81.2%)	89.8% (81.5%-95.2%)	57.7% (36.9%-76.6%)
Group4 >60 years	.584 (.45-.719)	88.2% (72.5%-96.7%)	28.6% (8.39%-58.1%)	75% (58.8%-87.3%)	50% (15.7%-84.3%)
Cut off	0.4				
Whole cohort	.784 (.739-.829)	71.8%(65.8%-77.3%)	85%(76.5%-91.4%)	92.3%(87.7%-95.7%)	54.5%(46.3%-62.5%)
Group1 < 30 years	.611(.512-.71)	22.2%(6.41%-47.6%)	100% (85.2%-100%)	100% (39.8%-100%)	62.2%(44.8%-77.5%)
Group2 30-39 years	.731(.632-.831)	52.5% (36.1%-68.5%)	93.8% (69.8%-99.8%)	95.5% (77.2%-99.9%)	44.1% (27.2%-62.1%)
Group3 40-49 years	.835 (.767-.904)	71.4% (59.4%-81.6%)	95.7% (78.1%-99.9%)	98% (89.6%-100%)	52.4% (36.4%-68%)
Group4 50-59 years	.761 (.658-.864)	85.6% (76.6%-92.1%)	66.7% (44.7%-84.4%)	90.6%(82.3%-95.8%)	55.2% (35.7%-73.6%)
Group4 >60 years	.748(.604-.891)	85.3% (68.9%-95%)	64.3% (35.1%-87.2%)	85.3% (68.9%-95%)	64.3%(35.1%-87.2%)
Cut off	0.5				
Whole cohort		61.5% (55.2%-67.5%)	90% (82.4%-95.1%)		
Group1 < 30 years	.583 (.495-.672)	16.7% (3.58%-41.4%)	100%(85.2%-100%)	100% (29.2%-100%)	60.5% (43.4%-76%)
Group2 30-39 years	.706(.607 .806)	47.5% (31.5%-63.9%)	93.8% (69.8%-99.8%)	95% (75.1%-99.9%)	41.7%(25.5%-59.2%)
Group3 40-49 years	.814 (.757-.871)	62.9% (50.5%-74.1%)	100% (85.2%-100%)	100% (92%-100%)	46.9% (32.5%-61.7%)
Group4 50-59 years	.74 (.644-.836)	68.9% (58.3%-78.2%)	79.2% (57.8%-92.9%)	92.5% (83.4%-97.5%)	40.4%(26.4%-55.7%)
Group4 >60 years	.754(.613-.895)	79.4% (62.1%-91.3%)	71.4%(41.9%-91.6%)	87.1%(70.2%-96.4%)	58.8% (32.9%-81.6%)

Cut off	0.6				
Whole cohort	.746(.705-.786)	57.1% (50.8%-63.3%)	92% (84.8%-96.5%)	94.7%(89.9%-97.7%)	46% (38.9%-53.2%)
Group1 < 30 years	.583(.495-.672)	16.7%(3.58%-41.4%)	100% (85.2%-100%)	100% (29.2%-100%)	60.5% (43.4%-76%)
Group2 30-39 years	.681 (.582-.78)	42.5% (27%-59.1%)	93.8%(69.8%-99.8%)	94.4% (72.7%-99.9%)	39.5%(24%-56.6%)
Group3 40-49 years	.793 (.735-.851)	58.6% (46.2%-70.2%)	100% (85.2%-100%)	100% (91.4%-100%)	44.2% (30.5%-58.7%)
Group4 50-59 years	.733 .642 .824	63.3% (52.5%-73.2%)	83.3% (62.6%-95.3%)	93.4%(84.1%-98.2%)	37.7% (24.8%-52.1%)
Group4 >60 years	.775(.642-.908)	76.5%(58.8%-89.3%)	78.6% (49.2%-95.3%)	89.7% (72.6%-97.8%)	57.9% (33.5%-79.7%)
Cut off	0.7				
Whole cohort	.717(.677-.757)	50.4% (44.1%-56.7%)	93% (86.1%-97.1%)	94.8%(89.5%-97.9%)	42.7% (36%-49.5%)
Group1 < 30 years	.583 (.495-.672)	16.7% (3.58%-41.4%)	100% (85.2%-100%)	100%(29.2%-100%)	60.5%(43.4%-76%)
Group2 30-39 years	.631 (.536-.727)	32.5% (18.6%-49.1%)	93.8%(69.8%-99.8%)	92.9% (66.1%-99.8%)	35.7%(21.6%-52%)
Group3 40-49 years	.771 (.713-.83)	54.3% (41.9%-66.3%)	100% (85.2%-100%)	100% (90.7%-100%)	41.8% (28.7%-55.9%)
Group4 50-59 years	.71(.625-.795)	54.4% (43.6%-65%)	87.5% (67.6%-97.3%)	94.2% (84.1%-98.8%)	33.9% (22.3%-47%)
Group4 >60 years	.746 (.61-.882)	70.6%(52.5%-84.9%)	78.6% (49.2%-95.3%)	88.9% (70.8%-97.6%)	52.4%)29.8%-74.3%)
Cut off	0.8				
Whole cohort	.704 (.668-.741)	44.8% (38.6%-51.2%)	96% (90.1%-98.9%)	96.6% (91.5%-99.1%)	40.9% (34.5%-47.4%)
Group1 < 30 years	.583 (.495-.672)	16.7% (3.58%-41.4%)	100% (85.2%-100%)	100%(29.2%-100%)	60.5%(43.4%-76%)
Group2 30-39 years	.662 (.589-.736)	32.5% (18.6%-49.1%)	100% (79.4%-100%)	100% (75.3%-100%)	37.2% (23%-53.3%)
Group3 40-49 years	.757 (.698-.816)	51.4% (39.2%-63.6%)	100% (85.2%-100%)	100%(90.3%-100%)	40.4% (27.6%-54.2%)
Group4 50-59 years	.697 (.621-.774)	47.8% (37.1%-58.6%)	91.7% (73%-99%)	95.6% (84.9%-99.5%)	31.9% (21.2%-44.2%)
Group4 >60 years	.693(.566-.821)	52.9% (35.1%-70.2%)	85.7% (57.2%-98.2%)	90%(68.3%-98.8%)	42.9% (24.5%-62.8%)
Cut off	0.9				
Whole cohort	.686(.653-.72)	39.3% (33.2%-45.6%)	98% (93%-99.8%)	98% (93%-99.8%)	39%(33%-45.4%)
Group1 < 30 years	.583 (.495-.672)	16.7% (3.58%-41.4%)	100% (85.2% -100%)	100%(29.2%-100%)	60.5%(43.4%-76%)

Group2 30-39 years	.638 (.567-.708)	27.5% (14.6%-43.9%)	100% (79.4%-100%)	100% (71.5%-100%)	35.6% (21.9%-51.2%)
Group3 40-49 years	.736 (.677-.795)	47.1%(35.1%-59.4%)	100% (85.2%-100%)	100% (89.4%-100%)	38.3% (26.1%-51.8%)
Group4 50-59 years	.706 (.654-.757)	41.1% (30.8%-52%)	100%(85.8%-100%)	100% (90.5%-100%)	31.2% (21.1%-42.7%)
Group4 >60 years	.649(.522-.777)	44.1% (27.2%-62.1%)	85.7% (57.2%-98.2%)	88.2% (63.6%-98.5%)	38.7% (21.8%-57.8%)
Cut off	10				
Whole cohort	.67(.639-.701)	34.9% (29%-41.2%)	99% (94.6%-100%)	98.9%(93.9%-100%)	37.6% (31.8%-43.8%)
Group1 < 30 years	.583(.495-.672)	16.7%(3.58%-41.4%)	100% (85.2%-100%)	100% (29.2%-100%)	60.5% (43.4%-76%)
Group2 40-49 years	.625(.557-.693)	25% (12.7%-41.2%)	100% (79.4%-100%)	100%(69.2%-100%)	34.8%(21.4%-50.2%)
Group3 50-59 years	.693(.635-.75)	38.6% (27.2%-51%)	100% (85.2%-100%)	100% (87.2%-100%)	34.8% (23.5%-47.6%)
Group4 50-59 years	.689 (.639-.739)	37.8% (27.8%-48.6%)	100% (85.8%-100%)	100%(89.7%-100%)	30% (20.3%-41.3%)
Group4 >60 years	.67 (.561-.779)	41.2%(24.6%-59.3%)	92.9% (66.1%-99.8%)	93.3% (68.1%-99.8%)	39.4% (22.9%-57.9%)

******************************Thumbdiff*********************************

Diagnostic accuracy of thumbdiff, All cutoff values, (female participants).

Age group	ROC	Sensitivity	Specificity	PPV	NPV
Cut off	0.5				
Whole cohort	0.747(0.696-0.798)	83% (77.8%- 87.4%)	66.3% (56.4%-75.3%)	85.7% (80.7%-89.8%)	61.6% (51.9%-70.6%)
Group1 < 30 years	0.72 (0.576-0.863)	66.7% (41%- 86.7%)	77.3% (54.6%-92.2%)	70.6 % (44%-89.7%)	73.9% (51.6%-89.8%)
Group2 30-39 years	0.626 (0.482-0.771)	69% (52.9%- 82.4%)	56.3% (29.9%-80.2%)	80.6 % (64%-91.8%)	40.9 % (20.7%-3.6%)
Group3 40-49 years	0.844 (0.766-0.922)	80.3 % (69.1%-88.8%)	88.5% (69.8%-97.6%)	95% (86.1%-99%)	62.2% (44.8%-77.5%)
Group4 50-59 years	0.8 (0.702-0.897)	92% (84.1%- 96.7%)	68% (46.5%- 85.1%)	90.9% (82.9%-96%)	70.8 % (48.9%-87.4%)
Group4 >60 years	0.557 (0.442-0.672)	91.4% (76.9%-98.2%)	20 % (4.33%- 48.1%)	72.7% (57.2%-85%)	50% (11.8%-88.2%)
Cut off	0.6				
Whole cohort	0.774 (0.726-0.822)	77.9% (72.2%-82.8%)	76.9 % (67.6%-84.6%)	89.1% (84.3%-92.9%)	58.8% (50.1%-67.2%)
Group1 < 30 years	0.765(0.631-0.899)	66.7% (41%- 86.7%)	86.4 % (65.1%-97.1%)	80% (51.9%-95.7%)	76% (54.9%-90.6%)
Group2 30-39 years	0.641 (0.502-0.781)	59.5 % (43.3%-74.4%)	68.8 % (41.3%- 89%)	83.3% (65.3%-94.4%)	39.3% (21.5%-59.4%)
Group3 40-49 years	0.835(0.762-0.908)	74.6 % (62.9%-84.2%)	92.3% (74.9%-99.1%)	96.4% (87.5%-99.6%)	57.1 % (41%-72.3%)
Group4 50-59 years	0.811 (0.718-0.904)	86.2% (77.1%-92.7%)	76% (54.9%- 90.6%)	92.6 % (84.6%-97.2%)	61.3% (42.2%-78.2%)
Group4 >60 years	0.69(0.552-0.829)	91.4% (76.9%-98.2%)	46.7% (21.3%-73.4%)	80% (64.4%-90.9%)	70 % (34.8%-93.3%)
Cut off	0.7				
Whole cohort	0.758 (0.712-0.804)	68% (61.9%- 73.7%)	83.7% (75.1%-90.2%)	91% (86%-94.7%)	51.8 % (44%-59.5%)
Group1 < 30 years	0.672(0.548-0.796)	38.9 % (17.3%-64.3%)	95.5 % (77.2%-99.9%)	87.5 % (47.3%-99.7%)	65.6% (46.8%-81.4%)
Group2 30-39 years	0.668(0.543-0.793)	52.4% (36.4%- 68%)	81.3% (54.4%- 96%)	88 % (68.8%-97.5%)	39.4% (22.9%-57.9%)
Group3 40-49 years	0.819 (0.752-0.885)	67.6% (55.5%-78.2%)	96.2 % (80.4%-99.9%)	98% (89.1%-99.9%)	52.1 % (37.2%-66.7%)
Group4 50-59 years	0.779 (0.687-0.871)	75.9% (65.5%-84.4%)	80% (59.3%- 93.2%)	93% (84.3%-97.7%)	48.8% (32.9%-64.9%)
Group4 >60 years	0.681(0.536 -0.826)	82.9 % (66.4%-93.4%)	53.3 % (26.6%-78.7%)	80.6 % (64%-91.8%)	57.1% (28.9%-82.3%)
Cut off	0.8				
Whole cohort	0.74 (0.696-0.784)	60.5% (54.2%-66.5%)	87.5% (79.6%-93.2%)	92.2 % (87%-95.8%)	47.6% (40.4%-55%)

Group1 < 30 years	0.667 (0.555-0.779)	33.3% (13.3%- 59%)	100 % (84.6%-100%)	100 % (54.1%-100%)	64.7% (46.5%-80.3%)
Group2 30-39 years	0.676 (0.562-0.789)	47.6% (32%- 63.6%)	87.5 % (61.7%-98.4%)	90.9% (70.8%-98.9%)	38.9% (23.1%-56.5%)
Group3 40-49 years	0.777(0.708-0.845)	59.2 % (46.8%-70.7%)	96.2 % (80.4%-99.9%)	97.7% (87.7%-99.9%)	46.3 % (32.6%-60.4%)
Group4 50-59 years	0.748(0.659-0.836)	65.5% (54.6%-75.4%)	84% (63.9%- 95.5%)	93.4 % (84.1%-98.2%)	41.2% (27.6%-55.8%)
Group4 >60 years	0.7 (0.555-0.845)	80% (63.1%- 91.6%0	60% (32.3%- 83.7%)	82.4 % (65.5%-93.2%)	56.3% (29.9%-80.2%)
Cut off	10.0				
Whole cohort	0.699 (0.657-0.741)	49.4% (43.1%-55.7%)	90.4% (83%- 95.3%)	92.6 % (86.8%-96.4%)	42.3% (35.8%-49.1%)
Group1 < 30 years	0.611(0.512-0.71)	22.2% (6.41%-47.6%)	100% (84.6%- 100%)	100% (39.8%-100%)	61.1% (43.5%-76.9%)
Group2 30-39 years	0.664 (0.551-0.777)	45.2% (29.8%-61.3%)	87.5% (61.7%-98.4%)	90.5% (69.6%-98.8%)	37.8% (22.5%-55.2%)
Group3 40-49 years	0.739 (0.681-0.798)	47.9 % (35.9%-60.1%)	100 % (86.8%-100%)	100% (89.7%-100%)	41.3% (29%-54.4%)
Group4 50-59 years	0.704 (0.621-0.788)	52.9 % (41.9%-63.7%)	88% (68.8%- 97.5%)	93.9% (83.1%-98.7%)	34.9% (23.3%-48%)
Group4 >60 years	0.648 (0.5-0.795)	62.9% (44.9%-78.5%)	66.7% (38.4%-88.2%)	81.5% (61.9%-93.7%)	43.5 % (23.2%-65.5%)
Cut off	1.3				
Whole cohort	0.665(0.632-0.699)	36% (30.1%- 42.2%)	97.1 % (91.8%-99.4%)	96.8% (91%-99.3%)	38.4% (32.5%-44.6%)
Group1 < 30 years	0.611(0.512-0.71)	22.2 % (6.41%-47.6%)	100% (84.6%- 100%)	100 % (39.8%-100%)	61.1% (43.5%-76.9%)
Group2 30-39 years	0.576 (0.488-0.664)	21.4% (10.3%-36.8%)	93.8 % (69.8%-99.8%)	90% (55.5%-99.7%)	31.3% (18.7%-46.3%)
Group3 40-49 years	0.69(0.633-0.747)	38% (26.8%- 50.3%)	100% (86.8%- 100%)	100 % (87.2%-100%)	37.1% (25.9%-49.5%)
Group4 50-59 years	0.695 (0.644-0.747)	39.1% (28.8%-50.1%)	100% (86.3%- 100%)	100 % (89.7%-100%)	32.1% (21.9%-43.6%)
Group4 >60 years	0.676 (0.554-0.799)	48.6% (31.4%- 66%)	86.7% (59.5%-98.3%)	89.5% (66.9%-98.7%)	41.9% (24.5%-60.9%)
Cut off	1.4				
Whole cohort	0.665 (0.634-0.696)	34% (28.2%- 40.2%)	99% (94.8%- 100%)	98.9% (93.8%-100%)	38.1 % (32.3%-44.2%)
Group1 < 30 years	0.611 (0.512 0.71)	22.2 % (6.41%-47.6%)	100 % (84.6%-100%)	100% (39.8%-100%)	61.1%) 43.5%-76.9%)
Group2 30-39 years	0.607(0.544-0.67)	21.4% (10.3%-36.8%)	100% (79.4%- 100%)	100 % (66.4%-100%)	32.7% (19.9%-47.5%)
Group3 40-49 years	0.676 0.(62-0.732)	35.2% (24.2%-47.5%)	100% (86.8%- 100%)	100 % (86.3%-100%)	36.1 % (25.1%-48.3%)
Group4 50-59 years	0.684(0.633-0.735)	36.8 % (26.7%-47.8%)	100 % (86.3%-100%)	100% (89.1%-100%)	31.3%)21.3%-42.6%)
Group4 >60 years	0.695 (0.589-0.801)	45.7% (28.8%-63.4%)	93.3% (68.1%-99.8%)	94.1% (71.3%-99.9%)	42.4% (25.5%-60.8%)

0.9, 1.1 and 1.2 ms cut off were removed as the number were similar to 0.8 ms, 1.0 ms , 1.3 ms respectively)

******************************Ringdiff ********************************

Diagnostic accuracy of ringdiff, All cutoff values, (female participants).

Age group	ROC	Sensitivity	Specificity	PPV	NPV
Cut off	0.4				
Whole cohort	.712(.661-.763)	67%(60.5%- 73%)	75.5% (66.2%-83.3%)	85.7% (79.8%-90.5%)	51%(42.9%-59%)
Group1 < 30 years	.651 (.521-.781)	38.9% (17.3%-64.3%)	91.3% (72%-98.9%)	77.8% (40%-97.2%)	65.6% (46.8%-81.4%)
Group2 30-39 years	.662 (.538-.785)	50% (33.4%-66.6%)	82.4% (56.6%-96.2%)	86.4% (65.1%-97.1%)	42.4% (25.5%-60.8%)
Group3 40-49 years	.768(.681-.856)	65.6% (52.7%-77.1%)	88% (68.8%-97.5%)	93.3% (81.7%-98.6%)	50% (34.6%-65.4%)
Group4 50-59 years	.681 (.575-.788)	74.7% (64%-83.6%)	61.5%(40.6%-79.8%)	86.1% (75.9%-93.1%)	43.2% (27.1%-60.5%)
Group4 >60 years	.667 (.522-.811)	86.7% (69.3%-96.2%)	46.7% (21.3%-73.4%)	76.5% (58.8%-89.3%)	63.6%(30.8%-89.1%)
Cut off	0.5				
Whole cohort	.72 (.673-.767)	60.1%(53.5%-66.4%)	84% (75.6%-90.4%)	89.2%(83.2%-93.6%)	48.9% (41.4%-56.4%)
Group1 < 30 years	.617 (.502-.732)	27.8% (9.69%-53.5%)	95.7% (78.1%-99.9%)	83.3% (35.9%-99.6%)	62.9% (44.9%-78.5%)
Group2 30-39 years	.665 (.552-.777)	44.7% (28.6%-61.7%)	88.2%(63.6%-98.5%)	89.5% (66.9%-98.7%)	41.7% (25.5%-59.2%)
Group3 40-49 years	.785(.713-.857)	60.9% (47.9%-72.9%)	96% (79.6%-99.9%)	97.5% (86.8%-99.9%)	49% (34.4%-63.7%)
Group4 50-59 years	.697 (.596-.798)	66.3% (55.1%-76.3%)	73.1% (52.2%-88.4%)	88.7% (78.1%-95.3%)	40.4% (26.4%-55.7%)
Group4 >60 years	.7 (.552-.848)	80% (61.4%-92.3%)	60% (32.3%-83.7%)	80% (61.4%-92.3%)	60% (32.3%-83.7%)
Cut off	0.6				
Whole cohort	.711 (.666-.756)	54.5% (47.9%-61%)	87.7% (79.9%-93.3%)	90.7% (84.6%-95%)	46.7% (39.6%-53.9%)
Group1 < 30 years	.589 (.482-.697)	22.2% (6.41%-47.6%)	95.7% (78.1%-99.9%)	80% (28.4%-99.5%)	61.1%(43.5%-76.9%)
Group2 30-39 years	.694(.596-.793)	44.7% 28.6% 61.7%	94.1% (71.3%-99.9%)	94.4%(72.7%-99.9%)	43.2% (27.1%-60.5%)
Group3 40-49 years	.722(.649-.795)	48.4% (35.8%-61.3%)	96% (79.6%-99.9%)	96.9% (83.8%0 99.9%)	42.1%(29.1%-55.9%)
Group4 50-59 years	.692(.594-.79)	61.4% (50.1%-71.9%)	89.5% (78.5%-96%)	89.5% (78.5%-96%)	38.5% (25.3%-53%)
Group4 >60 years	.767(.63-.903)	80%(61.4%-92.3%)	73.3% (44.9%-92.2%)	85.7% (67.3%-96%)	64.7% (38.3%-85.8%)

Cut off	0.7				
Whole cohort	.561 (.485-.65)	50.2% (43.6%-56.8%)	88.7% (81.1%-94%)	90.7% (84.3%-95.1%)	44.8% (37.9%-51.8%)
Group1 < 30 years	.611(.512-.71)	22.2%(6.41%-47.6%)	100% (85.2%-100%)	100% (39.8%-100%)	62.2% (44.8%-77.5%)
Group2 30-39 years	.668 (.57-.766)	39.5%(24%-56.6%)	94.1% (71.3%-99.9%)	93.8% (69.8%-99.8%)	41% (25.6%-57.9%)
Group3 40-49 years	.691 (.618-.763)	42.2% (29.9%-55.2%)	96% (79.6%-99.9%)	96.4% (81.7%-99.9%)	39.3% (27.1%-52.7%)
Group1+2+3	.676 (.628-.725)	38.3%(29.6%-47.6%)	96.9% (89.3%-99.6%)	95.8% (85.7%-99.5%)	46% (37.4%-54.7%)
Group4 50-59 years	.68(.582-.778)	59% (47.7%-69.7%)	76.9% (56.4%-91%)	89.1%(77.8%-95.9%)	37% (24.3%-51.3%)
Group1+2+3+4	.69(.645-.735)	46.8% (39.8%-53.9%)	91.2% (83.4%-96.1%)	92.2%(85.3%-96.6%)	43.5% (36.3%-50.8%)
Group4 >60 years	.733 (.592-.874)	73.3% (54.1%-87.7%)	73.3% (44.9%-92.2%)	73.3%(44.9%-92.2%)	57.9% (33.5%-79.7%)
Cut off	0.8				
Whole cohort	.68(.638-.723)	45.5% (39%-52.1%)	90.6%(83.3%-95.4%)	91.4% (84.7%-95.8%)	43% (36.5%-49.8%)
Group1 < 30 years	.583 (.495-.672)	16.7% (3.58%-41.4%)	100% (85.2%-100%)	100% (29.2%-100%)	60.5% (43.4%-76%)
Group2 30-39 years	.668 (.57-.766)	39.5%(24%-56.6%)	94.1%(71.3%-99.9%)	93.8% (69.8%-99.8%)	41% (25.6%-57.9%)
Group3 40-49 years	.635 (.514-.785)	39.1% (27.1%-52.1%)	96% (79.6%-99.9%)	96.2% (80.4%-99.9%)	38.1%(26.1%-51.2%)
Group4 50-59 years	.682 (.593-.771)	51.8% (40.6%-62.9%)	84.6% (65.1%-95.6%)	91.5% (79.6%-97.6%)	35.5%(23.7%-48.7%)
Group4 >60 years	.7 (.556-.844)	66.7%(47.2%-82.7%)	73.3% (44.9%-92.2%)	83.3% (62.6%-95.3%)	52.4% (29.8%-74.3%)
Cut off	0.9				
Whole cohort	.668 (.626-.709)	42.1% (35.6%-48.7%)	91.5% (84.5%-96%)	91.6% (84.6%-96.1%)	41.8% (35.4%-48.4%)
Group1 < 30 years	.583(.495-.672)	16.7% (3.58%-41.4%)	100%(85.2%-100%)	100%(29.2%-100%)	60.5% (43.4%-76%)
Group2 30-39 years	.655 (.558-.752)	36.8% (21.8%-54%)	94.1% (71.3%-99.9%)	93.3% (68.1%-99.8%)	40% (24.9%-56.7%)
Group3 40-49 years	.66 (.589-.731)	35.9% (24.3%-48.9%)	96% (79.6%-99.9%)	95.8%(78.9%-99.9%)	36.9% (25.3%-49.8%)
Group4 50-59 years	.677 (.595-.76)	47% (35.9%-58.3%)	88.5% (69.8%-97.6%)	92.9% (80.5%-98.5%)	34.3% (23.2%-46.9%)
Group4 >60 years	.683 (.538-.829)	63.3% (43.9%-80.1%)	73.3% (44.9%-92.2%)	82.6% (61.2%-95%)	50% (28.2%-71.8%)

Cut off	10				
Whole cohort	.673 (.635-.712)	40.3%(34%-46.9%)	94.3% (88.1%-97.9%)	94% (87.4%-97.8%)	41.8% (35.5%-48.4%)
Group1 < 30 years	.583 (.495-.672)	16.7% (3.58%-41.4%)	100% (85.2%-100%)	100% (29.2%=100%)	60.5% (43.4%-76%)
Group2 40-49 years	.671 (.595-.747)	34.2% (19.6%-51.4%)	100% (80.5%-100%)	100% (75.3%-100%)	40.5% (25.6%-56.7%)
Group3 50-59 years	.68(.62 -.739)	35.9% (24.3%-48.9%)	100% (86.3%-100%)	100% (85.2%-100%)	37.9% (26.2%-50.7%)
Group4 50-59 years	.684 (.609-.759)	44.6% (33.7%-55.9%)	92.3% (74.9%-99.1%)	94.9% (82.7%-99.4%)	34.3% (23.3%-46.6%)
Group4 >60 years	.667 (.521-.813)	60% (40.6%-77.3%)	73.3%(44.9%-92.2%)	81.8% (59.7%-94.8%)	47.8% (26.8%-69.4%)
	11				
Group4 >60 years	.667 .528 .805	53.3% 34.3% 71.7%	80% 51.9% 95.7%	84.2% 60.4% 96.6%	46.2% 26.6% 66.6%
	12				
Group4 >60 years	.733 (.643- .824)	46.7% (28.3%-65.7%)	100% (78.2%-100%)	100% (76.8%-100%)	48.4% (30.2%-66.9%)

**********************************CSI**********************************

Diagnostic accuracy of combined sensory index (CSI), All cutoff values, (female participants).

Age group	ROC	Sensitivity	Specificity	PPV	NPV
Cut off	0.9				
Whole cohort	.77(.719-.821)	83.6% (78.4%-88%)	70.4% (60.3%-79.2%)	87.8%(83%-91.7%)	62.7%(53%-71.8%)
Group1 < 30 years	.755(.629-.881)	55.6% (30.8% -78.5%)	95.5%(77.2%-99.9%)	90.9% (58.7%-99.8%)	72.4% (52.8%-87.3%)
Group2 30-39 years	.655 (.511-.798)	64.3% (48%-78.4%)	66.7% (38.4%-88.2%)	84.4% (67.2%-94.7%)	40% (21.1%-61.3%)
Group3 40-49 years	.876 (.802-.949)	83.8% (72.9%-91.6%)	91.3% (72%-98.9%)	96.6%(88.3%-99.6%)	65.6% (46.8%-81.4%)
Group4 50-59 years	.732(.626-.837)	92.2% (84.6%-96.8%)	54.2%(32.8%-74.4%)	88.3%(80%-94%)	65% (40.8%-84.6%)
Group4 >60 years	.643 (.52 -.766)	100% (89.1%-100%)	28.6%(8.39%-58.1%)	76.2%(60.5%-87.9%)	100% (39.8%-100%)
Cut off	1.0				
Whole cohort	.773(.723-.824)	81.2% (75.8%-85.8%)	73.5% (63.6%-81.9%)	88.6% (83.8%-92.4%)	60.5% (51.1%-69.3%)
Group1 < 30 years	.755 (.629-.881)	55.6%(30.8%-78.5%)	95.5% (77.2%-99.9%)	90.9% (58.7%-99.8%)	72.4%(52.8%-87.3%)
Group2 30-39 years	.664 (.526-.802)	59.5%(43.3%-74.4%)	73.3% (44.9%-92.2%)	86.2% (68.3%-96.1%)	39.3% (21.5%-59.4%)
Group3 40-49 years	.868 (.794-.943)	82.4% (71.2%-90.5%)	91.3%(72%-98.9%)	96.6%(88.1%-99.6%)	63.6% (45.1%-79.6%)
Group4 50-59 years	.757(.653-.861)	88.9% (80.5%-94.5%)	62.5% (40.6%-81.2%)	89.9%(81.7%-95.3%)	60% 38.7% 78.9%
Group4 >60 years	.643(.52 .766)	100% (89.1% -100)	28.6% (8.39%-58.1%)	76.2% (60.5%-87.9%)	100% (39.8%-100%)
Cut off	1.1				
Whole cohort	0.791 (0.743-0.839)	79.6% (74.1%- 84.4%)	78.6% (69.1% -86.2%)	90.5% (85.8%-94%)	60.2% (51.1%-68.7%)
Group1 < 30 years	.699 (.573- .826)	44.4% (21.5%-69.2%)	95.5% (77.2%- 99.9%)	88.9% (51.8%- 99.7%)	67.7% (48.6%-83.3%)
Group2 30-39 years	.698 (.569-.827)	59.5% (43.3%-74.4%)	80% (51.9%-95.7%)	89.3% (71.8%-97.7%)	41.4% (23.5%-61.1%)
Group3 40-49 years	.883 (.819-.946)	80.9% (69.5%-89.4%)	95.7% (78.1%-99.9%)	98.2% (90.4%-100%)	62.9% (44.9%-78.5%)

Group4 50-59 years	0.793 (0.694-.892)	87.8% (79.2%-93.7%)	70.8% (48.9%-87.4%)	91.9% (83.9%-96.7%)	60.7% (40.6%-78.5%)
Group4 >60 years	.679 (.548-.809)	100% (89.1%-100%)	35.7% (12.8%-64.9%)	78% (62.4%-89.4%)	100% (47.8%-100%)
Cut off	1.3				
Whole cohort	.793(.747-.839)	76%(70.2%-81.2%)	82.7% (73.7%-89.6%)	91.8% (87.2% -95.1%)	57.4%(48.8%-65.7%)
Group1 < 30 years	.699(.573-.826)	44.4% (21.5%-69.2%)	95.5% (77.2%-99.9%)	88.9% (51.8%-99.7%)	67.7% (48.6%-83.3%)
Group2 30-39 years	.707(.59-.824)	54.8% (38.7%-70.2%)	86.7% (59.5%-98.3%)	92% (74% -99%)	40.6% (23.7%-59.4%)
Group3 40-49 years	.861 (.794-.927)	76.5% (64.6% -85.9%)	95.7% (78.1%-99.9%)	98.1% (89.9%-100%)	57.9% (40.8%-73.7%)
Group4 50-59 years	.803 (.707-.898)	85.6% (76.6%-92.1%)	75% (53.3%-90.2%)	92.8%(84.9%-97.3%)	58.1% (39.1%-75.5%)
Group4 >60 years	.719 (.576-.861)	93.8%(79.2%-99.2%)	50% (23%-77%)	81.1% (64.8%-92%)	77.8% (40%-97.2%)
Cut off	1.4				
Whole cohort	.784(.738-.83)	73.2% (67.3%-78.6%)	83.7% (74.8%-90.4%)	92% (87.3%-95.3%)	55% (46.7%-63.2%)
Group1 < 30 years	.694 (.579-.81)	38.9%(17.3%-64.3%)	100% (84.6%-100%)	100% (59%-100%)	66.7%(48.2%-82%)
Group2 30-39 years	.707(.59 -.824)	54.8% (38.7% -70.2%)	86.7%(59.5%-98.3%)	92% (74% -99%)	40.6%(23.7%-59.4%)
Group3 40-49 years	.839 (.77-.907)	72.1% (59.9%-82.3%)	95.7% (78.1%-99.9%)	98% (89.4%-99.9%)	53.7% (37.4%-69.3%)
Group4 50-59 years	.792 (.695-.888)	83.3% (74%-90.4%)	75% (53.3%-90.2%)	92.6% (84.6%-97.2%)	54.5% (36.4%-71.9%)
Group4 >60 years	.703 (.558 -.848)	90.6% (75% -98%)	50% (23%-77%)	80.6%(64%-91.8%)	70% (34.8%-93.3%)
Cut off	1.5				
Whole cohort	.78 (.735-.82)	69.2%(63.1%-74.9%)	86.7% (78.4%-92.7%)	93% (88.3%-96.2%)	52.5% (44.5%-60.4%)
Group1 < 30 years	.639 (.532-.745)	27.8% (9.69%-53.5%)	100%(84.6%-100%)	100% (47.8%-100%)	62.9%(44.9%-78.5%)
Group2 30-39 years	.717(.616-.817)	50% (34.2%-65.8%)	93.3%(68.1%-99.8%)	95.5% (77.2%-99.9%)	40% (23.9%-57.9%)
Group3 40-49 years	.824(.754-.894)	69.1%(56.7%-79.8%)	95.7% (78.1%-99.9%)	97.9%(88.9%-99.9%)	51.2%(35.5%-66.7%)
Group4 50-59 years	.79(.697-.883)	78.9% (69%-86.8%)	79.2% (57.8%-92.9%)	93.4% (85.3%-97.8%)	50% (33.4%-66.6%)
Group4 >60 years	.739 (.595-.883)	90.6% (75% -98%)	57.1% (28.9%-82.3%)	82.9%(66.4%-93.4%)	72.7% (39%-94%)
Cut off	1.6				
Whole cohort	.77(.725-.814)	67.2% (61%- 73%)	86.7% (78.4%-92.7%)	92.8% (88%-96.1%)	50.9% (43.1%-58.7%)

Group1 < 30 years	.639(.532 - .745)	27.8%(9.69%- 53.5%)	100% (84.6%- 100%)	100% (47.8%- 100%)	62.9% (44.9%- 78.5%)
Group2 30-39 years	.705(.604- .805)	47.6% (32%- 63.6%)	93.3%(68.1%- 99.8%)	95.2%(76.2%- 99.9%)	38.9% (23.1%- 56.5%)
Group3 40-49 years	.794 .(723- .866)	63.2% (50.7%- 74.6%)	95.7% (78.1%- 99.9%)	97.7% (88%- 99.9)	46.8% (32.1%- 61.9%)
Group4 50-59 years	.79 (.697- .883)	78.9% (69%- 86.8%)	79.2% (57.8%- 92.9%)	93.4% (85.3%- 97.8%)	50% (33.4%- 66.6%)
Group4 >60 years	.739 (.595- .883)	90.6%(75%- 98%)	57.1% (28.9%- 82.3%)	82.9% (66.4%- 93.4%)	72.7%(39%- 94%)
Cut off	1.7				
Whole cohort	.775 (.733- .817)	65.2%(58.9%- 71.1%)	89.8% (82%- 95%)	94.2%(89.6%- 97.2%)	50.3% (42.6%- 57.9%)
Group1 < 30 years	.611 (.512- .71)	22.2% (6.41%- 47.6%)	100% (84.6%- 100%)	100% (39.8%- 100%)	61.1% (43.5%- 76.9%)
Group2 30-39 years	.705 (.604- .805)	47.6% (32%- 63.6%)	93.3% (68.1%- 99.8%)	95.2% (76.2%- 99.9%)	38.9% (23.1%- 56.5%)
Group3 40-49 years	.816 (.758- .874)	63.2% (50.7%- 74.6%)	100% (85.2%- 100%)	100%(91.8%- 100%)	47.9% (33.3%- 62.8%)
Group4 50-59 years	.789 (.7 - .878)	74.4% (64.2%- 83.1%)	83.3% (62.6%- 95.3%)	94.4% (86.2%- 98.4%)	46.5% (31.2%- 62.3%)
Group4 >60 years	.775(.635- .915)	90.6% (75% -98%)	64.3% (35.1%- 87.2%)	85.3% (68.9%- 95%)	75% (42.8%- 94.5%)
Cut off	2.0				
Whole cohort	.748 (.706- .79)	58.8% (52.4%- 65%)	90.8%(83.3%- 95.7%)	94.2% (89.3%- 97.3%)	46.4% (39.1%- 53.7%)
Group1 < 30 years	.611 (.512- .71)	22.2%(6.41%- 47.6%)	100%(84.6%- 100%)	100%(39.8%- 100%)	61.1%(43.5%- 76.9%)
Group2 30-39 years	.705 (.604- .805)	47.6% (32%- 63.6%)	93.3% (68.1%- 99.8%)	95.2% (76.2%- 99.9%)	38.9% (23.1%- 56.5%)
Group3 40-49 years	.772(.712- .832)	54.4% (41.9%- 66.5%)	100%(85.2%- 100%)	100% (90.5%- 100%)	42.6% (29.2%- 56.8%)
Group4 50-59 years	.756 (.665- .846)	67.8%(57.1%- 77.2%)	83.3% (62.6%-) 95.3%	93.8% (85%- 98.3%)	40.8% (27%- 55.8%)
Group4 >60 years	.748 (.605- .89)	78.1% (60%- 90.7%)	71.4% (41.9%- 91.6%)	86.2%(68.3%- 96.1%)	58.8% (32.9%- 81.6%)
Cut off	2.5				
Whole cohort	.7 (.66- .741)	47.2%(40.9%- 53.6%)	92.9% (85.8%- 97.1%)	94.4%(88.8%- 97.7%)	40.8% (34.3%- 47.6%)
Group1 < 30 years	.611 (.512- .71)	22.2%(6.41%- 47.6%)	100% (84.6%- 100%)	100% (39.8%- 100%)	61.1% (43.5%- 76.9%)
Group2 30-39 years	.657 (.558- .756)	38.1% (23.6%- 54.4%)	93.3% (68.1%- 99.8%)	94.1% (71.3%- 99.9%)	35% (20.6%- 51.7%)
Group3 40-49 years	.743 (.683- .802)	48.5%(36.2%- 61%)	100% (85.2%- 100%)	100% (89.4%- 100%)	39.7%(27%- 53.4%)
Group4 50-59 years	.714 (.637- .791)	51.1% (40.3%- 61.8%)	91.7% (73%- 99%)	95.8% (85.7%- 99.5%)	33.3% (22.2%- 46%)

Group4 >60 years	.654 (.504-.804)	59.4% (40.6%-76.3%)	71.4%(41.9%-91.6%)	82.6%(61.2%-95%)	43.5%(23.2%-65.5%)
Cut off	3.5				
Whole cohort	.649 (.619-.679)	30.8% (25.1%-36.9%)	99% (94.4%-100%)	98.7%(93.1%-100%)	35.9% (30.2%-42%)
Group1 < 30 years	.583 (.495-.672)	16.7%(3.58%-41.4%)	100% (84.6%-100%)	100% (29.2%-100%)	59.5% (42.1%-75.2%)
Group2 30-39 years	.595(.535-.655)	19% (8.6%-34.1%)	100% (78.2%-100%)	100% (63.1%-100%)	30.6% (18.3%-45.4%)
Group3 40-49 years	.669 .(612-.726)	33.8% (22.8%-46.3%)	100% (85.2%-100%)	100%(85.2%-100%)	33.8% (22.8%-46.3%)
Group4 50-59 years	.672 (.623-.722)	34.4% (24.7%-45.2%)	100% (85.8%-100%)	100% (88.8%-100%)	28.9% (19.5% -39.9%
Group4 >60 years	.652 (.542-.762)	37.5% (21.1%-56.3%)	92.9% (66.1%-99.8%)	92.3% (64%-99.8%)	39.4% (22.9%-57.9%)
