# Supplementary material for: Values and Diagnostic Accuracy of Electrodiagnostic Findings in Carpal Tunnel Syndrome Based on Age, Gender, and Diabetes
Source: Diagnostics (Basel). 2024 Jun 28;14(13):1381. doi: 10.3390/diagnostics14131381 (PMC11240809; doi:10.3390/diagnostics14131381)
Supplement: Supplementary file 1 [file diagnostics-14-01381-s001.zip › Table S8 Diagnostic accuracy of median latency at Digit II and comparative latency studies (COLSs), all cutoff values, (male participants).pdf]

**Table S8 Diagnostic accuracy of median latency at Digit II, All cutoff values, (male participants)**

| Age group | ROC | Sensitivity | Specificity | PPV | NPV |
|---|---|---|---|---|---|
| | | | | | |
| Cut off | 3.5 (ms) | | | | |
| Whole cohort | .737( .672-.802) | 77.9% (66.2%-87.1%) | 69.5%( 60.3%-77.6%) | 59.6%( 48.6%-69.8%) | 84.5% (75.8%-91.1%) |
| Group1 < 30 years | .489 .207 .77 | 25%( .631%-80.6%) | 72.7%( 39%-94%) | 25% (.631%-80.6%) | 72.7%( 39%-94%) |
| Group2 30-39 years | .89 (.741- 1) | 85.7%( 42.1%-99.6%) | 92.3% (74.9%-99.1%) | 75% (34.9%-96.8%) | 96% (79.6%-99.9%) |
| Group3 40-49 years | .757 (.601-.913) | 80% (44.4%-97.5%) | 71.4%( 51.3%-86.8%) | 50% (24.7%-75.3%) | 90.9%( 70.8%-98.9%) |
| Group4 50-59 years | .688( .562-.814) | 71% (52% -85.8%) | 66.7%( 44.7%-84.4%) | 73.3%( 54.1%-87.7%) | 64% (42.5%-82%) |
| Group4 >60 years | .741( .649-.834) | 100% (79.4%-100%) | 48.3% (29.4%-67.5%) | 51.6%( 33.1%-69.8%) | 100%( 76.8%-100%) |
| | | | | | |
| Cut off | 3.6 (ms) | | | | |
| Whole cohort | .72 (.652-.788) | 67.6% (55.2%-78.5%) | 76.3%( 67.6%-83.6%) | 62.2% (50.1%-73.2%) | 80.4%( 71.8%-87.3%) |
| Group1 < 30 years | .534 (.261-.807) | 25% (.631%-80.6%) | 81.8%( 48.2%-97.7%) | 33.3%( .84%-90.6%) | 75% (42.8%-94.5%) |
| Group2 30-39 years | .909 (.764 - 1) | 85.7%( 42.1%-99.6%) | 96.2%( 80.4%-99.9%) | 85.7%( 42.1%-99.6%) | 96.2% (80.4%-99.9%) |
| Group3 40-49 years | .675 (.495-.855) | 60%( 26.2%-87.8%) | 75% (55.1%-89.3%) | 46.2% (19.2%-74.9%) | 84% (63.9%-95.5%) |
| Group4 50-59 years | .681 (.557-.806) | 61.3%( 42.2%-78.2%) | 75% (53.3%-90.2%) | 76% (54.9%-90.6%) | 60% (40.6%-77.3%) |
| Group4 >60 years | .731 (.607-.854) | 87.5% (61.7%-98.4%) | 58.6%( 38.9%-76.5%) | 53.8% (33.4%-73.4%) | 89.5%( 66.9%-98.7%) |
| | | | | | |
| Cut off | 3.7 (ms) | | | | |
| Whole cohort | .731 (.664-.798) | 63.2%( 50.7%-74.6%) | 83.1%( 75%-89.3%) | 68.3%( 55.3%-79.4%) | 79.7%( 71.5%-86.4%) |
| Group1 < 30 years | .534 (.261-.807) | 25%( .631%-80.6%) | 81.8%( 48.2%-97.7%) | 33.3%( .84%-90.6%) | 75% (42.8%-94.5%) |
| Group2 30-39 years | .929 (.789-1) | 85.7%( 42.1%-99.6%) | 100%( 86.8%-100%) | 100% (54.1%-100%) | 96.3%( 81%-99.9%) |
| Group3 40-49 years | .593 (.415-.771) | 40% (12.2%-73.8%) | 78.6% (59%-91.7%) | 40% (12.2%-73.8%) | 78.6%( 59%-91.7%) |
| Group4 50-59 years | .723 (.607-.839) | 61.3%( 42.2%-78.2%) | 83.3%( 62.6%-95.3%) | 82.6%( 61.2%-95%) | 62.5%( 43.7%-78.9%) |

| | | | | | |
|---|---|---|---|---|---|
| Group4 >60 years | .768 (.639-.897) | 81.3%( 54.4%-96%) | 72.4% (52.8%-87.3%) | 61.9% (38.4%-81.9%) | 87.5% (67.6%-97.3%) |
| | | | | | |
| Cut off | 3.8 | | | | |
| Whole cohort | .729( .663-.796) | 60.3% (47.7%-72%) | 85.6%( 77.9%-91.4%) | 70.7%( 57.3%-81.9%) | 78.9% (70.8%-85.6%) |
| Group1 < 30 years | .58 (.319-.84) | 25% (.631%-80.6%) | 90.9% (58.7%-99.8%) | 50% (1.26%-98.7%) | 76.9%( 46.2%-- 95%) |
| Group2 30-39 years | .857 (.676-1) | 71.4% (29%-96.3%) | 100% (86.8%-100%) | 100% (47.8%-100%) | 92.9%( 76.5%-99.1%) |
| Group3 40-49 years | .629( .455-.802) | 40% (12.2%-73.8%) | 85.7% (67.3%-96%) | 50% (15.7%-84.3%) | 80% (61.4%-92.3%) |
| Group4 50-59 years | .707 (.59-.824) | 58.1%( 39.1%-75.5%) | 83.3% (62.6%-95.3%) | 81.8% (59.7%-94.8%) | 60.6%( 42.1%-77.1%) |
| Group4 >60 years | .768( .639-.897) | 81.3% (54.4%-96%) | 72.4%( 52.8%-87.3%) | 61.9% (38.4%-81.9%) | 87.5% (67.6%-97.3%) |
| | | | | | |
| Cut off | 3.9 | | | | |
| Whole cohort | .729 (.663-.796) | 60.3% (47.7%-72%) | 85.6%( 77.9%-91.4%) | 70.7%( 57.3%-81.9%) | 78.9%( 70.8%-85.6%) |
| Group1 < 30 years | .58 (.319-.84) | 25% (.631%-80.6%) | 90.9%( 58.7%-99.8%) | 50% (1.26%-98.7%) | 76.9%( 46.2%-95%) |
| Group2 30-39 years | .857 (.676-1) | 71.4%( 29%-96.3%) | 100% (86.8%-100%) | 100%( 47.8%-100%) | 92.9% (76.5%-99.1%) |
| Group3 40-49 years | .629 (.455-.802) | 40% (12.2%-73.8%) | 85.7%( 67.3%-96%) | 50% (15.7%-84.3%) | 80% (61.4%-92.3%) |
| Group4 50-59 years | .707 (.59-.824) | 58.1%( 39.1%-75.5%) | 83.3%( 62.6%-95.3%) | 81.8%( 59.7%-94.8%) | 60.6%( 42.1%-77.1%) |
| Group4 >60 years | .768 (.639-.897) | 81.3% (54.4%-96%) | 72.4%( 52.8%-87.3%) | 61.9%( 38.4%-81.9%) | 87.5% (67.6%-97.3%) |
| | | | | | |
| Cut off | 4.0 | | | | |
| Whole cohort | .729 (.663-.794) | 55.9%( 43.3%-67.9%) | 89.8%( 82.9%-94.6%) | 76% (61.8%-86.9%) | 77.9%( 70%-84.6%) |
| Group1 < 30 years | .58 (.319-.84) | 25%( .631%-80.6%) | 90.9% (58.7%-99.8%) | 50% (1.26%-98.7%) | 76.9% (46.2%-95% |
| Group2 30-39 years | .857( .676-1) | 71.4% (29%-96.3%) | 100% (86.8%-100%) | 100%( 47.8%-100%) | 92.9% (76.5%-99.1%) |
| Group3 40-49 years | .614 (.457-.772) | 30% (6.67%-65.2%) | 92.9%( 76.5%-99.1%) | 60% (14.7%-94.7%) | 78.8%( 61.1%-91%) |
| Group4 50-59 years | .728 (.617-.839) | 58.1%( 39.1%-75.5%) | 87.5% (67.6%-97.3%) | 85.7%( 63.7%-97%) | 61.8%( 43.6%-77.8%) |
| Group4 >60 years | .74 (.601-.88) | 68.8%( 41.3%-89%) | 79.3%( 60.3%-92%) | 64.7%( 38.3%-85.8%) | 82.1% (63.1%-93.9%) |
| | | | | | |
| Cut off | 4.1 | | | | |

| | | | | | |
|---|---|---|---|---|---|
| Whole cohort | .712( .647-.776) | 50% (37.6%-62.4%) | 92.4%( 86%-96.5%) | 79.1%( 64%-90%) | 76.2%( 68.4%-82.9%) |
| Group1 < 30 years | | | | | |
| Group2 30-39 years | .786 (.588-.984) | 57.1%( 18.4%-90.1%) | 100% (86.8%-100%) | 100% (39.8%-100%) | 89.7%( 72.6%-97.8%) |
| Group3 40-49 years | .582 (.447-.717) | 20% (2.52%-55.6%) | 96.4%( 81.7%-99.9%) | 66.7%( 9.43%-99.2%) | 77.1% (59.9%-89.6%) |
| Group4 50-59 years | .712 (.6 -.823) | 54.8% (36%-72.7%) | 87.5%( 67.6%-97.3%) | 85% (62.1%-96.8%) | 60% (42.1%-76.1%) |
| Group4 >60 years | .758 (.621-.894) | 68.8% (41.3%-89%) | 82.8%( 64.2%-94.2%) | 68.8%( 41.3%-89%) | 82.8% (64.2%-94.2%) |
| | | | | | |
| Cut off | 4.2 | | | | |
| Whole cohort | .701 (.637-.765) | 47.1% (34.8%-59.6%) | 93.2% (87.1%-97%) | 80% (64.4%-90.9%) | 75.3%( 67.5%-82.1%) |
| Group1 < 30 years | | | | | |
| Group2 30-39 years | .714 (.516-.912) | 42.9%( 9.9%-81.6%) | 100% (86.8%-100%) | 100% (29.2%-100%) | 86.7%( 69.3%-96.2%) |
| Group3 40-49 years | .582 (.447-.717) | 20% (2.52%-55.6%) | 96.4%( 81.7%-99.9%) | 66.7%( 9.43%-99.2%) | 77.1%( 59.9%-89.6%) |
| Group4 50-59 years | .712 (.6 -.823) | 54.8% (36%-72.7%) | 87.5%( 67.6%-97.3%) | 85% (62.1%-96.8%) | 60% (42.1%-76.1%) |
| Group4 >60 years | .744 (.605-.882) | 62.5%( 35.4%-84.8%) | 86.2%( 68.3%-96.1%) | 71.4%( 41.9%-91.6%) | 80.6%( 62.5%-92.5%) |
| | | | | | |
| Cut off | 4.3 | | | | |
| Whole cohort | .703 (.64-.765) | 45.6% (33.5%-58.1%) | 94.9% (89.3%-98.1%) | 83.8%( 68%-93.8%) | 75.2%( 67.4%-81.9%) |
| Group1 < 30 years | | | | | |
| Group2 30-39 years | .714 (.516-.912) | 42.9%( 9.9%-81.6%) | 100% (86.8%-100%) | 100%( 29.2%-100%) | 86.7%( 69.3%-96.2%) |
| Group3 40-49 years | .582 (.447-.717) | 20% (2.52%-55.6%) | 96.4% (81.7%-99.9%) | 66.7%( 9.43%-99.2%) | 77.1%( 59.9%-89.6%) |
| Group4 50-59 years | .716 (.611-.822) | 51.6%( 33.1%-69.8%) | 91.7%( 73%-99%) | 88.9%( 65.3%-98.6%) | 59.5% (42.1%-75.2%) |
| Group4 >60 years | .761 (.626-.896) | 62.5% (35.4%-84.8%) | 89.7%( 72.6% 97.8%) | 76.9%( 46.2%-95%) | 81.3%( 63.6%-92.8%) |
| | | | | | |
| Cut off | 4.4 | | | | |
| Whole cohort | .707 (.644-.769) | 45.6% (33.5%-58.1%) | 95.8% (90.4%-98.6%) | 86.1% (70.5%-95.3%) | 75.3%( 67.6%-82%) |
| Group1 < 30 years | | | | | |

| | | | | | |
|---|---|---|---|---|---|
| Group2 30-39 years | .714 (.516-.912) | 42.9% (9.9%-81.6%) | 100%( 86.8%-100%) | 100%(29.2%-100%) | 86.7%( 69.3%-96.2%) |
| Group3 40-49 years | .582 (.447-.717) | 20% (2.52%-55.6%) | 96.4%( 81.7%-99.9%) | 66.7% (9.43%-99.2%) | 77.1%( 59.9%-89.6%) |
| Group4 50-59 years | .737 (.639-.836) | 51.6%( 33.1%-69.8%) | 95.8% (78.9%-99.9%) | 94.1% (71.3%-99.9%) | 60.5% (43.4%-76%) |
| Group4 >60 years | .761 (.626-.896) | 62.5%( 35.4%-84.8%) | 89.7%( 72.6%-97.8%) | 76.9% (46.2%-95%) | 81.3%( 63.6%-92.8%) |
| | | | | | |
| Cut off | 4.5 | | | | |
| Whole cohort | .689( .628-.75) | 41.2%( 29.4%-53.8%) | 96.6% (91.5%-99.1%) | 87.5%( 71%-96.5%) | 74% (66.4%-80.8%) |
| Group1 < 30 years | | | | | |
| Group2 30-39 years | .714 (.516-.912) | 42.9%( 9.9%-81.6%) | 100%( 86.8%-100%) | 100% (29.2%-100%) | 86.7%( 69.3%-96.2%) |
| Group3 40-49 years | .582 (.447-.717) | 20% (2.52%-55.6%) | 96.4%( 81.7%-99.9%) | 66.7% (9.43%-99.2%) | 77.1%( 59.9%-89.6%) |
| Group4 50-59 years | .721 (.623-.819) | 48.4%( 30.2%-66.9%) | 95.8%( 78.9%-99.9%) | 93.8%( 69.8%-99.8%) | 59% (42.1%-74.4%) |
| Group4 >60 years | .716 (.581-.85) | 50% (24.7%-75.3%) | 93.1%( 77.2%-99.2%) | 80% (44.4%-97.5%) | 77.1%( 59.9%-89.6%) |
| | | | | | |
| Cut off | 4.6 | | | | |
| Whole cohort | .674 (.614-.735) | 38.2% (26.7%-50.8%) | 96.6%( 91.5%-99.1%) | 86.7% (69.3%-96.2%) | 73.1%( 65.4%-79.9%) |
| Group1 < 30 years | | | | | |
| Group2 30-39 years | .643 (.462-.824) | 28.6% (3.67%-71%) | 100% (86.8%-100%) | 100% (15.8%-100%) | 83.9%( 66.3%-94.5%) |
| Group3 40-49 years | .582 (.447-.717) | 20% (2.52%-55.6%) | 96.4%( 81.7%-99.9%) | 66.7%( 9.43%-99.2%) | 77.1% (59.9%-89.6%) |
| Group4 50-59 years | .705( .607-.803) | 45.2% 27.3%-64%) | 95.8%( 78.9%-99.9%) | 93.3% (68.1%-99.8%) | 57.5% (40.9%-73%) |
| Group4 >60 years | .716 (.581-.85) | 50% (24.7%-75.3%) | 93.1% (77.2%-99.2%) | 80% (44.4%-97.5%) | 77.1% (59.9%-89.6%) |
| | | | | | |
| Cut off | 4.7 | | | | |
| Whole cohort | .652( .593-.711) | 33.8%( 22.8%-46.3%) | 96.6% (91.5%-99.1%) | 85.2%( 66.3%-95.8%) | 71.7%( 64%-78.5%) |
| Group1 < 30 years | | | | | |
| Group2 30-39 years | .571 (.431-.711) | 14.3% (.361%-57.9%) | 100%( 86.8%-100%) | 100% (2.5%-100%) | 81.3% (63.6%-92.8%) |
| Group3 40-49 years | .582 (.447-.717) | 20% (2.52%-55.6%) | 96.4% (81.7%-99.9%) | 66.7% (9.43%-99.2%) | 77.1% (59.9%-89.6%) |

| | | | | | |
|---|---|---|---|---|---|
| Group4 50-59 years | .689 (.592-.786) | 41.9%( 24.5%-60.9%) | 95.8%( 78.9%-99.9%) | 92.9% (66.1%-99.8%) | 56.1% (39.7%-71.5%) |
| Group4 >60 years | .684 (.55 -.818) | 43.8%( 19.8%-70.1%) | 93.1%( 77.2%-99.2%) | 77.8%( 40%-97.2%) | 75% (57.8%-87.9%) |
| | | | | | |
| Cut off | 4.8 | | | | |
| Whole cohort | .656 (.598-.715) | 33.8% (22.8%-46.3%) | 97.5% (92.7%-99.5%) | 88.5%( 69.8%-97.6%) | 71.9% (64.2%-78.7%) |
| Group1 < 30 years | | | | | |
| Group2 30-39 years | .571 (.431-.711) | 14.3%( .361%-57.9%) | 100% (86.8%-100%) | 100% (2.5%-100%) | 81.3%( 63.6%-92.8%) |
| Group3 40-49 years | .6( .469-.731) | 20% (2.52%-55.6%) | 100% (87.7%-100%) | 100% (15.8%-100%) | 77.8% (60.8%-89.9%) |
| Group4 50-59 years | .689 (.592-.786) | 41.9% (24.5%-60.9%) | 95.8%( 78.9%-99.9%) | 92.9%( 66.1%-99.8%) | 56.1%( 39.7%-71.5%) |
| Group4 >60 years | .684 (.55-.818) | 43.8%( 19.8%-70.1%) | 93.1%( 77.2%-99.2%) | 77.8%( 40%-97.2%) | 75% (57.8%-87.9%) |
| | | | | | |
| Cut off | 4.9 | | | | |
| Whole cohort | .665( .608-.722) | 33.8% (22.8%-46.3%) | 99.2%( 95.4%-100%) | 95.8%( 78.9%-99.9%) | 72.2%( 64.7%-79%) |
| Group1 < 30 years | | | | | |
| Group2 30-39 years | .571 (.431-.711) | 14.3%( .361%-57.9%) | 100% (86.8%-100%) | 100% (2.5%-100%) | 81.3% (63.6%-92.8%) |
| Group3 40-49 years | .6 (.469 -.731) | 20% (2.52%-55.6%) | 100%( 87.7%-100%) | 100%( 15.8%-100%) | 77.8%( 60.8%-89.9%) |
| Group4 50-59 years | .689 (.592-.786) | 41.9%( 24.5%-60.9%) | 95.8% (78.9%-99.9%) | 92.9%( 66.1%-99.8%) | 56.1% (39.7%-71.5%) |
| Group4 >60 years | .719 (.593-.844) | 43.8%( 19.8%-70.1%) | 100% (88.1%-100%) | 100% (59%-100%) | 76.3% (59.8%-88.6%) |
| | | | | | |
| Cut off | 5.0 | | | | |
| Whole cohort | .662( .606-.718) | 32.4% (21.5%-44.8%) | 100% (96.9%-100%) | 100%( 84.6%-100%) | 72% (64.4%-78.7%) |
| Group1 < 30 years | | | | | |
| Group2 30-39 years | .571 .431 .711 | 14.3% (.361%-57.9%) | 100% (86.8%-100%) | 100% (2.5%-100%) | 81.3% (63.6%-92.8%) |
| Group3 40-49 years | .6 (.469-.731) | 20% (2.52%-55.6%) | 100%( 87.7%-100%) | 100% (15.8%-100%) | 77.8% (60.8%-89.9%) |
| Group4 50-59 years | .694 (.606-.781) | 38.7%( 21.8%-57.8%) | 100% (85.8%-100%) | 100%( 73.5%-100%) | 55.8%( 39.9%-70.9%) |
| Group4 >60 years | .719 (.593-.844) | 43.8% (19.8%-70.1%) | 100% (88.1%-100%) | 100%( 59%-100%) | 76.3% (59.8%-88.6%) |

********************************Palmdiff***********************************

**Diagnostic accuracy of palmdiff, All cutoff values, (male participants).**

| Age group | ROC | Sensitivity | Specificity | PPV | NPV |
|---|---|---|---|---|---|
| Cut off | 0.4 | | | | |
| Whole cohort | .81 (.75 -.87) | 83.1% (71.7%- 91.2%) | 78.9%( 70%- 86.1%) | 70.1% (58.6%- 80%) | 88.7%( 80.6%- 94.2%) |
| Group1 < 30 years | .705 (.408- 1) | 50% (6.76%- 93.2%) | 90.9% (58.7%- 99.8%) | 66.7%( 9.43%- 99.2%) | 83.3% (51.6%- 97.9%) |
| Group2 30-39 years | .938 (.87- 1) | 100% (63.1%- 100%) | 87.5%( 67.6%- 97.3%) | 72.7%( 39%- 94%) | 100% (83.9%- 100%) |
| Group3 40-49 years | .764 (.604- .923) | 72.7% (39%- 94%) | 80% (59.3%- 93.2%) | 61.5%( 31.6%- 86.1%) | 87% (66.4%- 97.2%) |
| Group4 50-59 years | .752 (.628- .875) | 80.8% (60.6%- 93.4%) | 69.6% (47.1%- 86.8%) | 75% (55.1%- )89.3%) | 76.2% (52.8%- 91.8%) |
| Group4 >60 years | .834 .(728- .94) | 93.8%( 69.8%- 99.8%) | 73.1% (52.2%- 88.4%) | 68.2% (45.1%- 86.1%) | 95% (75.1%- 99.9%) |
| | | | | | |
| Cut off | 0.5 | | | | |
| Whole cohort | .762 (.696- .828) | 66.2% (53.4%- 77.4%) | 86.2% (78.3%- 92.1%) | 74.1%( 61%- 84.7%) | 81% (72.7%- 87.7%) |
| Group1 < 30 years | 0.75 (0.467- 1) | 50% (6.76%- 93.2%) | 100% (71.5%- 100%) | 100% (15.8%- 100%) | 84.6% (54.6%-98.1%) |
| Group2 30-39 years | .813 (0.633- 0.992) | 62.5% (24.5%- 91.5%) | 100% (85.8%- 100%) | 100% (47.8%- 100%) | 88.9% (70.8%-97.6%) |
| Group3 40-49 years | .784 .(627- .94) | 72.7% (39% -94%) | 84% (63.9%- 95.5%) | 66.7% (34.9%- 90.1%) | 87.5% (67.6%- 97.3%) |
| Group4 50-59 years | .68 (.55 - .809) | 57.7% (36.9%- 76.6%) | 57.7% (36.9%- 76.6%) | 75% (50.9%- 91.3%) | 62.1%( 42.3%- 79.3%) |
| Group4 >60 years | .791 (.662- .92) | 81.3% (54.4%- 96%) | 76.9% (56.4%- 91%) | 68.4%( 43.4%- 87.4%) | 87% (66.4%- 97.2%) |
| Cut off | 0.6 | | | | |
| Whole cohort | .759 (.693- .824) | 60% (47.1%- 72%) | 91.7%( 84.9%- 96.2%) | 81.3% (67.4%- 91.1%) | 79.4%( 71.2%- 86.1%) |
| Group1 < 30 years | .625 (.38 - .87) | 25% (.631%- 80.6%) | 100% (71.5%- 100%) | 100%( 2.5%- 100%) | 78.6%( 49.2%- 95.3%) |
| Group2 30-39 years | .75 (.565- .935) | 50% (15.7%- 84.3%) | 100% (85.8%- 100%) | 100% (39.8%- 100%) | 85.7%( 67.3%- 96%) |
| Group3 40-49 years | .804 (.651- .956) | 72.7% (39% -94%) | 88% (68.8%- 97.5%) | 72.7% (39%- 94%) | 88% (68.8%- 97.5%) |
| Group4 50-59 years | .685( .564- .805) | 50% (29.9%- 70.1%) | 87% (66.4%- 97.2%) | 81.3% (54.4%- 96%) | 60.6% (42.1%- 77.1%) |
| Group4 >60 years | .849( .732- .965) | 81.3% (54.4%- 96%) | 88.5% (69.8%- 97.6%) | 81.3% (54.4%- 96%) | 88.5% (69.8%- 97.6%) |

| | | | | | |
|---|---|---|---|---|---|
| | | | | | |
| Cut off | 0.7 | | | | |
| Whole cohort | .733 (.667 - .798) | 53.8% (41%-66.3%) | 92.7%( 86%-96.8%) | 81.4% (66.6%-91.6%) | 77.1% (68.9%-84%) |
| Group1 < 30 years | .625 (.38 - .87) | 25% (.631%-80.6%) | 100%( 71.5%-100%) | 100%( 2.5%-100%) | 78.6%( 49.2%-95.3%) |
| Group2 30-39 years | .688 (.508-.867) | 37.5% (8.52%-75.5%) | 100% (85.8%-100%) | 100% (29.2%-100%) | 82.8% (64.2%-94.2%) |
| Group3 40-49 years | .733 (.569-.896) | 54.5% (23.4% -83.3%) | 92% (74 99%-) | 75% (34.9%-96.8%) | 82.1% (63.1%-93.9%) |
| Group4 50-59 years | .666 (.545-.786) | 46.2% (26.6%-66.6%) | 87% (66.4%-97.2%) | 80% (51.9%-95.7%) | 58.8% (40.7%-75.4%) |
| Group4 >60 years | .849 (.732-.965) | 81.3% (54.4%-96%) | 88.5% (69.8%-97.6%) | 81.3% (54.4%-96%) | 88.5% (69.8%-97.6%) |
| | | | | | |
| Cut off | 0.8 | | | | |
| Whole cohort | .702 (.636-.768) | 47.7%( 35.1%-60.5%) | 92.7%( 86%-96.8%) | 79.5%( 63.5%-90.7%) | 74.8% (66.6%-81.9%) |
| Group1 < 30 years | | | | | |
| Group2 30-39 years | .688 (.508-.867) | 37.5% (8.52%-75.5%) | 100% (85.8%-100%) | 100%( 29.2%-100%) | 82.8% (64.2%-94.2%) |
| Group3 40-49 years | .687 (.524-.851) | 45.5% (16.7%-76.6%) | 92% (74 99%-) | 71.4% (29%-96.3%) | 79.3% (60.3%-92%) |
| Group4 50-59 years | .646 (.527-.766) | 42.3% (23.4%-63.1%) | 87% (66.4%-97.2%) | 78.6% (49.2%-95.3%) | 57.1% (39.4%-73.7%) |
| Group4 >60 years | .817 .(691-.944) | 75%( 47.6%-92.7%) | 88.5% (69.8% -97.6%) | 80%( 51.9%-95.7%) | 85.2% (66.3%-95.8%) |
| | | | | | |
| Cut off | 0.9 | | | | |
| Whole cohort | .703 (.639-.768) | 46.2% (33.7%-59%) | 46.2%( 33.7%-59%) | 83.3% (67.2%-93.6%) | 74.6% (66.5%-81.7%) |
| Group1 < 30 years | | | | | |
| Group2 30-39 years | .688( .508-.867) | 37.5% (8.52%-75.5%) | 100% (85.8%-100%) | 100% (29.2%-100%) | 82.8% (64.2%-94.2%) |
| Group3 40-49 years | .642( .483-.8) | 36.4% (10.9%-69.2%) | 92% (74%-99%) | 66.7%( 22.3%-95.7%) | 76.7% (57.7%-90.1%) |
| Group4 50-59 years | .668( .555-.781) | 42.3% (23.4%-63.1%) | 91.3% (72% -98.9%) | 84.6% (54.6%-98.1%) | 58.3% (40.8%-74.5%) |
| Group4 >60 years | .837( .715-.958) | 75% (47.6%-92.7%) | 92.3% (74.9%-99.1%) | 85.7% (57.2%-98.2%) | 85.7% (67.3%-96%) |
| | | | | | |
| Cut off | 1.0 | | | | |
| Whole cohort | .677 (.614-.74) | 40% (28% -52.9%) | 95.4% (89.6%-98.5%) | 83.9%( 66.3%-94.5%) | 72.7% (64.7%-79.8%) |

| | | | | | |
|---|---|---|---|---|---|
| Group1 < 30 years | | | | | |
| Group2 30-39 years | .688 (.508-.867) | 37.5%( 8.52%-75.5%) | 100%( 85.8%-100%) | 100% (29.2%-100%) | 82.8%( 64.2%-94.2%) |
| Group3 40-49 years | .662 (.508-.816) | 36.4% (10.9%-69.2%) | 96% (79.6%-99.9%) | 80% (28.4%-99.5%) | 77.4% (58.9%-90.4%) |
| Group4 50-59 years | .649 (.537-.761) | 38.5%( 20.2%-59.4%) | 91.3% (72% -98.9%) | 83.3% (51.6%-97.9%) | 56.8%( 39.5%-72.9%) |
| Group 2+3+4 | | | | | |
| Group4 >60 years | .743 (.607-.879) | 56.3% (29.9%-80.2%) | 92.3% (74.9%-99.1%) | 81.8%( 48.2%-97.7%) | 77.4% (58.9%-90.4%) |
| | | | | | |
| Cut off | 1.1 | | | | |
| Whole cohort | 95.4%(89.6%-98.5%) | 38.5% (26.7%-51.4%) | 95.4%( 89.6%-98.5%) | 83.3% (65.3%-94.4%) | 72.2% (64.2%-79.4%) |
| Group1 < 30 years | | | | | |
| Group2 30-39 years | .688 (.508-.867) | 37.5%( 8.52%-75.5%) | 100% (85.8%-100%) | 100% (29.2%-100%) | 82.8%( 64.2%-94.2%) |
| Group3 40-49 years | .662( .508 -.816) | 36.4% (10.9%-69.2%) | 96% (79.6%-99.9%) | 80% (28.4%-99.5%) | 77.4% (58.9%-90.4%) |
| Group4 50-59 years | .649 (.537-.761) | 38.5%( 20.2%-59.4%) | 91.3% (72%-98.9%) | 83.3% (51.6%-97.9%) | 56.8% (39.5%-72.9%) |
| Group4 >60 years | .712 (.575-.848) | 50% (24.7%-75.3%) | 92.3% (74.9%-99.1%) | 80% (44.4%-97.5%) | 75% (56.6%-88.5%) |
| | | | | | |
| Cut off | 1.2 | | | | |
| Whole cohort | .648 (.588-.707) | 32.3% (21.2%-45.1%) | 97.2%( 92.2%-99.4%) | 87.5% (67.6%-97.3%) | 70.7% (62.7%-77.8%) |
| Group1 < 30 years | | | | | |
| Group2 30-39 years | .688( .508-.867) | 37.5% (8.52%-75.5%) | 100% (85.8%-100%) | 100% (29.2%-100%) | 82.8%( 64.2%-94.2%) |
| Group3 40-49 years | .616( .473-.76) | 27.3% (6.02% -61%) | 96% (79.6%-99.9%) | 75% (19.4%-99.4%) | 75% (56.6%-88.5%) |
| Group4 50-59 years | 0.651 (0.549-0.754) | 34.6% (17.2%- 55.7%) | 95.7% (78.1%- 99.9%) | 90% (55.5%- 99.7%) | 56.4% (39.6%-72.2%) |
| Group4 >60 years | .668 (.54-.796) | 37.5% (15.2%-64.6%) | 96.2% (80.4%-99.9%) | 85.7% (42.1%-99.6%) | 71.4%( 53.7%-85.4%) |
| | | | | | |
| Cut off | 1.3 | | | | |
| Whole cohort | .64 (.581-.699) | 30.8% (19.9%-43.4%) | 97.2% (92.2%-99.4%) | 87% (66.4%-97.2%) | 70.2% (62.2%-77.4%) |
| Group1 < 30 years | | | | | |

| | | | | | |
|---|---|---|---|---|---|
| Group2 30-39 years | .688 (.508-.867) | 37.5% (8.52%-75.5%) | 100%( 85.8%-100%) | 100% (29.2%-100%) | 82.8%( 64.2%-94.2%) |
| Group3 40-49 years | .616 (.473-.76) | 27.3% (6.02%-61%) | 96% (79.6%-99.9%) | 75% (19.4%-99.4%) | 75% (56.6%-88.5%) |
| Group4 50-59 years | .632( .532-.732) | 30.8%( 14.3% - 51.8%) | 95.7% (78.1% - 99.9%) | 88.9% (51.8%-99.7%) | 55% (38.5%-70.7%) |
| Group4 >60 years | .668( .54-.796) | 37.5% (15.2%-64.6%) | 96.2%( 80.4%-99.9%) | 85.7% (42.1%-99.6%) | 71.4% (53.7%-85.4%) |
| | | | | | |
| Cut off | 1.4 | | | | |
| Whole cohort | .64 (.581-.699) | 30.8% (19.9%-43.4%) | 97.2% (92.2%-99.4%) | 87%( 66.4%-97.2%) | 70.2%( 62.2%-77.4%) |
| Group1 < 30 years | | | | | |
| Group2 30-39 years | .688 (.508-.867) | 37.5%( 8.52%-75.5%) | 100%( 85.8%-100%) | 100%( 29.2%-100%) | 82.8%( 64.2% -94.2%) |
| Group3 40-49 years | .616 (.473-.76) | 27.3% (6.02%-61%) | 96% (79.6%-99.9%) | 75% (19.4%-99.4%) | 75% (56.6%-88.5%) |
| Group4 50-59 years | .632 (.532-.732) | 30.8%( 14.3%-51.8%) | 95.7% (78.1%-99.9%) | 88.9%( 51.8%-99.7%) | 55% (38.5%-70.7%) |
| Group4 >60 years | .668 (.54-.796) | 37.5%( 15.2%-64.6%) | 96.2% (80.4%-99.9%) | 85.7% (42.1%-99.6%) | 71.4% (53.7%-85.4%) |
| | | | | | |
| Cut off | 0.15 | | | | |
| Whole cohort | .617( .561-.673) | 26.2%( 16% - 38.5%) | 97.2%( 92.2%-99.4%) | 85% (62.1%-96.8%) | 68.8%( 60.9%-76%) |
| Group1 < 30 years | | | | | |
| Group2 30-39 years | .625 (.465-.785) | 25% (3.19%-65.1%) | 100%( 85.8%-100%) | 100% )15.8%-100%) | 80% (61.4%-92.3%) |
| Group3 40-49 years | .616 (.473-.76) | 27.3%( 6.02%-61%) | 96% (79.6%-99.9%) | 75% (19.4%-99.4%) | 75%( 56.6%-88.5%) |
| Group4 50-59 years | .594 (.501-.687) | 23.1%( 8.97%-43.6%) | 95.7%( 78.1%-99.9%) | 85.7% (42.1%-99.6%) | 52.4%( 36.4%-68%) |
| Group4 >60 years | .668 (.54-.796) | 37.5% (15.2%-64.6%) | 96.2% (80.4%-99.9%) | 85.7% (42.1%-99.6%) | 71.4% (53.7%-85.4%) |
| | | | | | |
| Cut off | 0.16 | | | | |
| Whole cohort | .586 (.535-.638) | 20%( 11.1%-31.8%) | 97.2%( 92.2%-99.4%) | 81.3% (54.4%-96%) | 67.1%( 59.2%-74.3%) |
| Group1 < 30 years | | | | | |
| Group2 30-39 years | .563( .44-.685) | 12.5%( .316%-52.7%) | 100% (85.8%-100%) | 100%( 2.5%-100%) | 77.4% (58.9%-90.4%) |
| Group3 40-49 years | .616 (.473-.76) | 27.3% (6.02%-61%) | 96% (79.6%-99.9%) | 75% (19.4%-99.4%) | 75%( 56.6%-88.5%) |

| | | | | | |
|---|---|---|---|---|---|
| Group4 50-59 years | .555 (.473-.638) | 15.4%( 4.36%-34.9%) | 95.7% (78.1%-99.9%) | 80% (28.4%-99.5%) | 50% (34.6%-65.4%) |
| Group4 >60 years | .637 (.514-.76) | 31.3% (11%-58.7%) | 96.2%( 80.4%-99.9%) | 83.3%( 35.9%-99.6%) | 69.4%( 51.9%-83.7%) |
| Cut off | 0.17 | | | | |
| Whole cohort | .583( .534-.632) | 18.5%( 9.92%-30%) | 98.2% (93.5%-99.8%) | 85.7%( 57.2%-98.2%) | 66.9%( 59%-74.1%) |
| Group1 < 30 years | | | | | |
| Group2 30-39 years | .563( .44-.685) | 12.5% (.316%-52.7%) | 100% (85.8%-100%) | 100%( 2.5%-100%) | 77.4% (58.9%-90.4%) |
| Group3 40-49 years | .616 (.473-.76) | 27.3%( 6.02%-61%) | 96% (79.6%-99.9%) | 75% (19.4%-99.4%) | 75% (56.6%-88.5%) |
| Group4 50-59 years | .558 (.495-.62) | 11.5%( 2.45%-30.2%) | 100% (85.2%-100%) | 100% (29.2%-100%) | 50% (34.9%-65.1%) |
| Group4 >60 years | .637 (.514-.76) | 31.3% (11%-58.7%) | 96.2%( 80.4%-99.9%) | 83.3% (35.9%-99.6%) | 69.4% (51.9%-83.7%) |
| | | | | | |
| Cut off | 0.18 | | | | |
| Whole cohort | .537 (.499-.575) | 9.23%( 3.46%-19%) | 98.2% (93.5%-99.8%) | 75% (34.9%-96.8%) | 64.5% (56.7%-71.7%) |
| Group1 < 30 years | | | | | |
| Group2 30-39 years | .563 (.44-.685) | 12.5% (.316%-52.7%) | 100%( 85.8%-100%) | 100% (2.5%-100%) | 77.4%( 58.9%-90.4%) |
| Group3 40-49 years | .616 (.473-.76) | 27.3%( 6.02%-61%) | 96% (79.6%-99.9%) | 75% (19.4%-99.4%) | 75% (56.6%-88.5%) |
| Group4 50-59 years | .519 (.482-.557) | 3.85% (.0973%-19.6%) | 100%( 85.2%0-100%) | 100%( 2.5%-100%) | 47.9%( 33.3%-62.8%) |
| Group4 >60 years | .512 (.44-.584) | 6.25% (.158%-30.2%) | 96.2% (80.4%-99.9%) | 50% (1.26%-98.7%) | 62.5% (45.8%-77.3%) |
| | | | | | |

******************************Thumbdiff*******************************

**Diagnostic accuracy of thumbdiff, All cutoff values, (male participants).**

| Age group | ROC | Sensitivity | Specificity | PPV | NPV |
|---|---|---|---|---|---|
| Cut off | 0.5 | | | | |
| Whole cohort | .705( .645-.764) | 89.6% (79.7%-95.7%) | 51.4% (41.7%-61%) | 52.6% (43.1%-62.1%) | 89.1% 78.8% 95.5% |
| Group1 < 30 years | .764 (.48- 1) | 75% (19.4%-99.4%) | 77.8%( 40%-97.2%) | 60%( 14.7%-94.7%) | 87.5%( 47.3%-99.7%) |
| Group2 30-39 years | .771( .615 -.927) | 87.5% (47.3%-99.7%) | 66.7%( 44.7%-84.4%) | 46.7%( 21.3%-73.4%) | 94.1% (71.3%-99.9%) |
| Group3 40-49 years | .635 (.5 -.77) | 90% (55.5%-99.7%) | 37% (19.4%-57.6%) | 34.6% (17.2%-55.7%) | 90.9%( 58.7%-99.8%) |
| Group4 50-59 years | .637( .516-.757) | 85.7% (67.3%-96%) | 41.7% (22.1%-63.4%) | 63.2% (46% -78.2%) | 71.4%( 41.9%-91.6%) |
| Group4 >60 years | .759 (.663-.855) | 100%( 80.5%-100%) | 51.9% (31.9%-71.3%) | 56.7% (37.4%-74.5%) | 100% (76.8%-100%) |
| Cut off | 0.6 | | | | |
| Whole cohort | .741 (.678-.803) | 85.1% (74.3%-92.6%) | 63.1% (53.4%-72%) | 58.2%( 47.8%-68.1%) | 87.5% (78.2%-93.8%) |
| Group1 < 30 years | 0.875 (0.63- 1.0) | 75% (19.4%- 99.4%) | 100% ( 66.4%- 100%) | 100% ( 29.2%- 100%) | 90% (55.5%-99.7%) |
| Group2 30-39 years | .75 (.567-.933) | 75% (34.9%-96.8%) | 75% (53.3%-90.2%) | 50% (21.1%-78.9%) | 90% (68.3%-98.8%) |
| Group3 40-49 years | .691 .(554-.828) | 90% (55.5%-99.7%) | 48.1% (28.7%-68.1%) | 39.1% (19.7%-61.5%) | 92.9%( 66.1%-99.8%) |
| Group4 50-59 years | .705 (.58-.831) | 78.6% (59%-91.7%) | 62.5%( 40.6%-81.2%) | 71% (52%-85.8%) | 71.4% (47.8%-88.7%) |
| Group4 >60 years | .778 (.682-.873) | 100% (80.5%-100%) | 55.6% (35.3%-74.5%) | 58.6% (38.9%-76.5%) | 100% (78.2%-100%) |
| | | | | | |
| Cut off | 0.7 | | | | |
| Whole cohort | .762 (.697-.827) | 77.6% (65.8%-86.9%) | 74.8% (65.6%-82.5%) | 65%( 53.5%-75.3%) | 84.7%( 76%-91.2%) |
| Group1 < 30 years | .75 (.467- 1) | 50% (6.76%-93.2%) | 100% 6(6.4%-100%) | 100% (15.8%-100%) | 81.8% (48.2%-97.7%) |
| Group2 30-39 years | .792 (.614-.969) | 75% (34.9%-96.8%) | 83.3% (62.6%-95.3%) | 60% (26.2%-87.8%) | 90.9% (70.8%-98.9%) |
| Group3 40-49 years | .77 (.615 -.926) | 80% (44.4%-97.5%) | 74.1% (53.7%-88.9%) | 53.3% (26.6%-78.7%) | 90.9% (70.8%-98.9%) |
| Group4 50-59 years | .729 .(605-.853) | 75% (55.1%-89.3%) | 70.8% (48.9%-87.4%) | 75% (55.1%-89.3%) | 70.8%( 48.9%-87.4%) |
| Group4 >60 years | .756( .634-.878) | 88.2% (63.6%-98.5%) | 63% (42.4%-80.6%) | 60% (38.7%-78.9%) | 89.5%( 66.9%-98.7%) |
| | | | | | |

| | | | | | |
|---|---|---|---|---|---|
| Cut off | 0.8 | | | | |
| Whole cohort | .77 .(705-.834) | 74.6% (62.5%-84.5%) | 79.3% (70.5%-86.4%) | 68.5% (56.6%-78.9%) | 83.8%( 75.3%-) 90.3% |
| Group1 < 30 years | .75( .467- 1) | 50% (6.76%-93.2%) | 100% (66.4%-100%) | 100% (15.8%-100%) | 81.8% (48.2%-97.7%) |
| Group2 30-39 years | .813 (.638-.987) | 75% (34.9%-96.8%) | 87.5% (67.6%-97.3%) | 66.7% (29.9%-) 92.5% | 91.3% (72%-98.9%) |
| Group3 40-49 years | .807 (.657-.958) | 80% (44.4%-97.5%) | 81.5% (61.9%-93.7%) | 61.5% (31.6%-86.1%) | 91.7% (73%-99%) |
| Group4 50-59 years | .714( .589-.839) | 67.9%( 47.6%-84.1%) | 75%( 53.3%-90.2%) | 76% (54.9%-90.6%) | 66.7%( 46%-83.5%) |
| Group4 >60 years | .775( .654-.895) | 88.2% (63.6%-98.5%) | 66.7% (46%-83.5%) | 62.5%( 40.6%-81.2%) | 90%( 68.3%-98.8%) |
| | | | | | |
| Cut off | 0.9 | | | | |
| Whole cohort | .758( .692-.824) | 68.7% (56.2%-) 79.4% | 82.9%( 74.6%-89.4%) | 70.8%( 58.2%-81.4%) | 81.4%( 73%-88.1%) |
| Group1 < 30 years | .75( .467- 1) | 50% (6.76%-93.2%) | 100% (66.4%-)100%) | 100% (15.8%-100%) | 81.8%( 48.2%-97.7%) |
| Group2 30-39 years | .792( .608-.976) | 62.5% (24.5%-91.5%) | 95.8% (78.9%-99.9%) | 83.3% (35.9%-99.6%) | 88.5%( 69.8%-97.6%) |
| Group3 40-49 years | .807 (.657-.958) | 80%( 44.4%-97.5%) | 81.5% (61.9%-93.7%) | 61.5% (31.6%-86.1%) | 91.7%( 73%-99%) |
| Group4 50-59 years | .699( .575-.823) | 60.7%( 40.6%-78.5%) | 79.2% (57.8%-92.9%) | 77.3% (54.6%-92.2%) | 63.3% (43.9%-80.1%) |
| Group4 >60 years | .764( .635-.892) | 82.4%( 56.6%-96.2%) | 70.4% (49.8%-86.2%) | 63.6% (40.7%-82.8%) | 86.4% (65.1%-97.1%) |
| | | | | | |
| Cut off | 1.0 | | | | |
| Whole cohort | .756 (.691-.821) | 61.2% (48.5%-72.9%) | 90.1% (83%-94.9%) | 78.8%( 65.3%-88.9%) | 79.4% (71.2%-86.1%) |
| Group1 < 30 years | .75( .467- 1) | 50%( 6.76%-93.2%) | 100%( 66.4%-100%) | 100% (15.8%-100%) | 81.8%( 48.2%-97.7%) |
| Group2 30-39 years | .667 (.483-.851) | 37.5%( 8.52%-75.5%) | 95.8% (78.9%-99.9%) | 75% (19.4%-99.4%) | 82.1% (63.1%-93.9%) |
| Group3 40-49 years | .776( .611-.94) | 70% (34.8%-93.3%) | 85.2%( 66.3%-95.8%) | 63.6% (30.8%-89.1%) | 88.5% (69.8%-97.6%) |
| Group4 50-59 years | 0.747 (0.644-0.85) | 53.6% (33.9%- 72.5%) | 95.8% (78.9%- 99.9%) | 93.8% (69.8%- 99.8%) | 63.9% (46.2%-79.2%) |
| Group 2+3+4 | | | | | |
| Group4 >60 years | .819( .7-.939) | 82.4% (56.6%-96.2%) | 81.5%( 61.9%-93.7%) | 73.7%( 48.8%-90.9%) | 88%( 68.8%-97.5%) |
| | | | | | |
| Cut off | 1.1 | | | | |
| Whole cohort | .736 (.67-.801) | 55.2% (42.6%-67.4%) | 91.9%( 85.2%-96.2%) | 80.4% (66.1%-90.6%) | 77.3% (69.2%-84.1%) |

| | | | | | |
|---|---|---|---|---|---|
| Group1 < 30 years | .75( .467- 1) | 50%( 6.76%- 93.2%) | 100%( 66.4%- 100%) | 100% (15.8%- 100%) | 81.8% (48.2%- 97.7%) |
| Group2 30-39 years | 0.688 (0.508- 0.867) | 37.5% (8.52%- 75.5%) | 100% (85.8%-100%) | 100% (29.2%- 100%) | 82.8% (64.2%-94.2%) |
| Group3 40-49 years | .676 (.499- .853) | 50% (18.7%- 81.3%) | 85.2% (66.3%- 95.8%) | 55.6%( 21.2%- 86.3%) | 82.1% (63.1%- 93.9%) |
| Group4 50-59 years | .729 (.626- .832) | 50% (30.6%- 69.4%) | 95.8%( 78.9%- 99.9%) | 93.3%( 68.1%- 99.8%) | 62.2% (44.8%- 77.5) |
| Group4 >60 years | .808( .684- .933) | 76.5% (50.1%- 93.2%) | 85.2% (66.3%- 95.8%) | 76.5% (50.1%- 93.2%) | 85.2% (66.3%- 95.8%) |
| | | | | | |
| Cut off | 1.2 | | | | |
| Whole cohort | .719 (.655- .783) | 49.3% (36.8%- 61.8%) | 94.6% (88.6%- 98%) | 84.6% (69.5%- 94.1%) | 75.5%( 67.5%- 82.4%) |
| Group1 < 30 years | .625 (.38 - .87) | 25% (.631% - 80.6%) | 100%( 66.4%- 100%) | 100%( 2.5%- 100%) | 75% (42.8%- 94.5%) |
| Group2 30-39 years | .688 .(508- .867) | 37.5% (8.52%- 75.5%) | 100%( 85.8%- 100%) | 100%( 29.2%- 100%) | 82.8%( 64.2%- 94.2%) |
| Group3 40-49 years | .613 (.455- .771) | 30% (6.67%- 65.2%) | 92.6% (75.7%- 99.1%) | 60% (14.7%- 94.7%) | 78.1% (60%- 90.7%) |
| Group4 50-59 years | 0.729 (0.626- 0.832) | 50% (30.6%- 69.4%) | 95.8% (78.9%- 99.9%) | 93.3% (68.1%- 99.8%) | 62.2% (44.8%- 77.5%) |
| | | | | | |
| Group4 >60 years | .797 (.67 - .924) | 70.6% (44%- 89.7%) | 88.9%( 70.8%- 97.6%) | 80% (51.9%- 95.7%) | 82.8% (64.2%- 94.2%) |
| | | | | | |
| Cut off | 1.3 | | | | |
| Whole cohort | .704( .641 - .768) | 46.3% (34%- 58.9%) | 94.6%( 88.6%- 98%) | 83.8% (68%- 93.8%) | 74.5% (66.4%- 81.4%) |
| Group1 < 30 years | | | | | |
| Group2 30-39 years | .688 (.508- .867) | 37.5% (8.52%- 75.5%) | 100%( 85.8%- 100%) | 100%( 29.2%- 100%) | 82.8% (64.2%- 94.2%) |
| Group3 40-49 years | .613 (.455- .771) | 30% (6.67%- 65.2%) | 92.6% (75.7%- 99.1%) | 60% (14.7%- 94.7%) | 78.1%( 60% - 90.7%) |
| Group4 50-59 years | .729 .(626 - .832) | 50% (30.6%- 69.4%) | 95.8%( 78.9%- 99.9%) | 93.3%( 68.1%- 99.8%) | 62.2% (44.8%- 77.5%) |
| Group4 >60 years | .768( .636- .9) | 64.7% (38.3%- 85.8%) | 88.9% (70.8%- 97.6%) | 78.6% (49.2%- 95.3%) | 80% (61.4%- 92.3%) |
| | | | | | |
| Cut off | 1.4 | | | | |
| Whole cohort | .701 .(638- .764) | 44.8% (32.6%- 57.4%) | 95.5%( 89.8%- 98.5%) | 85.7% (69.7%- 95.2%) | 74.1%( 66.1%- 81.1%) |
| Group1 < 30 years | | | | | |

| | | | | | |
|---|---|---|---|---|---|
| Group2 30-39 years | .688 (.508-.867) | 37.5%( 8.52%-75.5%) | 100%( 85.8%-100%) | 100% (29.2%-100%) | 82.8% (64.2%-94.2%) |
| Group3 40-49 years | .581( .446-.717) | 20% (2.52%-55.6%) | 96.3% (81%-99.9%) | 66.7% (9.43%-99.2%) | 76.5% (58.8%-89.3%) |
| Group4 50-59 years | .729( .626-.832) | 50% (30.6%-69.4%) | 95.8% (78.9%-99.9%) | 93.3% (68.1%-99.8%) | 62.2%( 44.8%-77.5%) |
| Group4 >60 years | .768( .636-.9) | 64.7% (38.3%-85.8%) | 88.9% 70.8%-97.6%) | 78.6% (49.2%-95.3%) | 80%( 61.4%-92.3%) |
| | | | | | |
| Cut off | 1.5 | | | | |
| Whole cohort | .688 (.627-.749) | 40.3% (28.5%-53%) | 97.3% (92.3%-99.4%) | 90% (73.5%-97.9%) | 73% (65.1%-79.9%) |
| Group1 < 30 years | | | | | |
| Group2 30-39 years | .688 (.508-.867) | 37.5% (8.52%-75.5%) | 100% (85.8%-100%) | 100% (29.2%-100%) | 82.8% (64.2%-94.2%) |
| Group3 40-49 years | .6 (.469-.731) | 20% (2.52%-55.6%) | 100% (87.2%-100%) | 100% (15.8%-100%) | 77.1% (59.9%-89.6%) |
| Group4 50-59 years | .711 (.609-.814) | 46.4% (27.5%-66.1%) | 95.8% (78.9%-99.9%) | 92.9% (66.1%-99.8%) | 60.5% (43.4%-76%) |
| Group4 >60 years | .728 (.595-.86) | 52.9% (27.8%-77%) | 92.6% (75.7%-99.1%) | 81.8% (48.2%-97.7%) | 75.8% (57.7%-88.9%) |
| | | | | | |
| | | | | | |
| Cut off | 1.6 | | | | |
| Whole cohort | .681 (.62-.741) | 38.8% (27.1%-51.5%) | 97.3% (92.3%-99.4%) | 89.7% (72.6%-97.8%) | 72.5% (64.6%-79.5%) |
| Group1 < 30 years | | | | | |
| Group2 30-39 years | .688 (.508-.867) | 37.5% (8.52%-75.5%) | 100% (85.8%-100%) | 100% (29.2%-100%) | 82.8% (64.2%-94.2%) |
| Group3 40-49 years | .6 (.469-.731) | 20% (2.52%-55.6%) | 100% (87.2%-100%) | 100% (15.8%-100%) | 77.1% (59.9%-89.6%) |
| Group4 50-59 years | .693 (.592-.795) | 42.9% (24.5%-62.8%) | 95.8% (78.9%-99.9%) | 92.3% (64%-99.8%) | 59% (42.1%-74.4%) |
| Group4 >60 years | .728 (.595-.86) | 52.9% (27.8%-77%) | 92.6% (75.7%-99.1%) | 81.8% (48.2%-97.7%) | 75.8% (57.7%-88.9%) |
| | | | | | |
| Cut off | 1.7 | | | | |
| Whole cohort | .673 (.613-.733) | 37.3% (25.8%-50%) | 97.3% (92.3%-99.4%) | 89.3% (71.8%-97.7%) | 72% (64.1%-79%) |
| Group1 < 30 years | | | | | |
| Group2 30-39 years | .688 (.508 .867) | 37.5% (8.52%-75.5%) | 100% (85.8%-100%) | 100% (29.2%-100%) | 82.8% (64.2%-94.2%) |
| Group3 40-49 years | .6 (.469-.731) | 20% (2.52%-55.6%) | 100% (87.2%-100%) | 100% (15.8%-100%) | 77.1% (59.9%-89.6%) |

| | | | | | |
|---|---|---|---|---|---|
| Group4 50-59 years | .676 (.575-.776) | 39.3% (21.5%-59.4%) | 95.8% (78.9%-99.9%) | 91.7% (61.5%-99.8%) | 57.5% (40.9%-73%) |
| Group4 >60 years | .728 (.595-.86) | 52.9% (27.8%-77%) | 92.6% (75.7%-99.1%) | 81.8% (48.2%-97.7%) | 75.8% (57.7%-88.9%) |
| | | | | | |
| | | | | | |
| Cut off | 1.8 | | | | |
| Whole cohort | .648 (.59-.705) | 31.3% (20.6%-43.8%) | 98.2% (93.6%-99.8%) | 91.3% (72%-98.9%) | 70.3% (62.5%-77.4%) |
| Group1 < 30 years | | | | | |
| Group2 30-39 years | .688 (.508-.867) | 37.5% (8.52%-75.5%) | 100% (85.8%-100%) | 100% (29.2%-100%) | 82.8% (64.2%-94.2%) |
| Group3 40-49 years | .6 (.469-.731) | 20% (2.52%-55.6%) | 100% (87.2%-100%) | 100% (15.8%-100%) | 77.1% (59.9%-89.6%) |
| Group4 50-59 years | .64 (.543-.737) | 32.1% (15.9%-52.4%) | 95.8% (78.9%-99.9%) | 90% (55.5%-99.7%) | 54.8% (38.7%-70.2%) |
| Group4 >60 years | .687 (.561-.813) | 41.2% (18.4%-67.1%) | 96.3% (81%-99.9%) | 87.5% (47.3%-99.7%) | 72.2% (54.8%-85.8%) |
| | | | | | |
| Cut off | 1.9 | | | | |
| Whole cohort | .648 (.59-.705) | 31.3% (20.6%-43.8%) | 98.2% (93.6%-99.8%) | 91.3% (72%-98.9%) | 70.3% (62.5%-77.4%) |
| Group1 < 30 years | | | | | |
| Group2 30-39 years | .688 (.508-.867) | 37.5% (8.52%-75.5%) | 100% (85.8%-100%) | 100% (29.2%-100%) | 82.8% (64.2%-94.2%) |
| Group3 40-49 years | .6 (.469-.731) | 20% (2.52%-55.6%) | 100% (87.2%-100%) | 100% (15.8%-100%) | 77.1% (59.9%-89.6%) |
| Group4 50-59 years | .64 (.543-.737) | 32.1% (15.9%-52.4%) | 95.8% (78.9%-99.9%) | 90% (55.5%-99.7%) | 54.8% (38.7%-70.2%) |
| Group4 >60 years | .687 (.561-.813) | 41.2% (18.4%-67.1%) | 96.3% (81%-99.9%) | 87.5% (47.3%-99.7%) | 72.2% (54.8%-85.8%) |
| | | | | | |
| Cut off | 2.0 | | | | |
| Whole cohort | .633 (.577-.689) | 28.4% (18%-40.7%) | 98.2% (93.6%-99.8%) | 90.5% (69.6%-98.8%) | 69.4% (61.6%-76.5%) |
| Group1 < 30 years | | | | | |
| Group2 30-39 years | .625 (.465-.785) | 25% (3.19%-65.1%) | 100% (85.8%-100%) | 100% (15.8%-100%) | 80% (61.4%-92.3%) |
| Group3 40-49 years | .6 (.469-.731) | 20% (2.52%-55.6%) | 100% (87.2%-100%) | 100% (15.8%-100%) | 77.1% (59.9%-89.6%) |
| Group4 50-59 years | .64 (.543-.737) | 32.1% (15.9%-52.4%) | 95.8% (78.9%-99.9%) | 90% (55.5%-99.7%) | 54.8% (38.7%-70.2%) |
| Group4 >60 years | .658 (.535-.781) | 35.3% (14.2%-61.7%) | 96.3% (81%-99.9%) | 85.7% (42.1%-99.6%) | 70.3% (53%-84.1%) |

| | | | | | |
|---|---|---|---|---|---|
| | | | | | |
| Cut off | 2.2 | | | | |
| Group4 >60 years | .658 (.535-.781) | 35.3% (14.2%-61.7%) | 96.3% (81%-99.9%) | 85.7% (42.1%-99.6%) | 70.3% (53%-84.1%) |
| | | | | | |
| Cut off | 2.5 | | | | |
| Group4 >60 years | .647 (.535-.759) | 29.4% (10.3%-56%) | 100% (87.2%-100%) | 100% (47.8%-100%) | 69.2% (52.4%-83%) |

***************************Ringdiff*****************************

**Diagnostic accuracy of ringdiff, All cutoff values, (male participants).**

| Age group | ROC | Sensitivity | Specificity | PPV | NPV |
|---|---|---|---|---|---|
| Cut off | 0.4 | | | | |
| Whole cohort | .754 .(683 - .824) | 75%( 61.6%- 85.6%) | 75.7% (66.3%-) 83.6% | 62.7% (50% - 74.2%) | 84.8% (75.8%- 91.4%) |
| Group1 < 30 years | .764 (.48- 1) | 75% (19.4%- 99.4%) | 77.8%( 40%- 97.2%) | 60% (14.7%- 94.7%) | 87.5%( 47.3%- 99.7%) |
| Group2 30-39 years | .795( .602- .988) | 71.4% (29%- 96.3%) | 87.5% (67.6%- 97.3%) | 62.5% (24.5%- 91.5%) | 91.3% (72%- 98.9%) |
| Group3 40-49 years | .741 (.569- .914) | 70% (34.8%- 93.3%) | 78.3%( 56.3%- 92.5%) | 58.3% (27.7%- 84.8%) | 85.7% (63.7%- 97%) |
| Group4 50-59 years | .728 (.599- .856) | 76% (54.9%- 90.6%) | 69.6%( 47.1%- 86.8%) | 73.1% (52.2% - 88.4%) | 72.7% (49.8%- 89.3%) |
| Group4 >60 years | .733( .571- .896) | 80% (44.4%- 97.5%) | 66.7% (44.7%- 84.4%) | 50% (24.7%- 75.3%) | 88.9% (65.3%- 98.6%) |
| | | | | | |
| Cut off | 0.5 | | | | |
| Whole cohort | .752 (.68- .824) | 67.9% (54%- 79.7%) | 82.5%( 73.8%- 89.3%) | 67.9% (54%- 79.7%) | 82.5% (73.8%- 89.3%) |
| Group1 < 30 years | .75 (0.467- 1) | 50% (6.76% -93.2%) | 100% ( 66.4%- 100%) | 100% (15.8%- 100%) | 81.8% (48.2%- 97.7%) |
| Group2 30-39 years | .765 (.563- .967) | 57.1% (18.4%- 90.1%) | 95.8% (78.9%- 99.9%) | 80% (28.4%- 99.5%) | 88.5%( 69.8%- 97.6%) |
| Group3 40-49 years | .785 (.619- .95) | 70% (34.8%- 93.3%) | 87% (66.4%- 97.2%) | 70% (34.8%- 93.3%) | 87% (66.4%- 97.2%) |
| Group4 50-59 years | .688 (.554- .822) | 68% (46.5%- 85.1%) | 69.6% (47.1%- 86.8%) | 70.8% (48.9%- 87.4%) | 66.7%( 44.7%- 84.4%) |
| Group4 >60 years | .754( .594- .914) | 80%( 44.4%- 97.5%) | 70.8% (48.9%- 87.4%) | 53.3% 26.6% 78.7% | 89.5% (66.9%- 98.7%) |
| Cut off | 0.6 | | | | |
| Whole cohort | .71 (.637- .78) | 53.6% (39.7%- 67%) | 88.3%( 80.5%- 93.8%) | 71.4%( 55.4% - 84.3%) | 77.8%( 69.2%- 84.9%) |
| Group1 < 30 years | .625 (.38 - .87) | 25%( .631%- 80.6%) | 100% (66.4%- 100%) | 100%( 2.5%- 100%) | 75%( 42.8%- 94.5%) |
| Group2 30-39 years | .714 (.516- .912) | 42.9% (9.9% -81.6%) | 100% ( 85.8%- 100%) | 100% (29.2%- 100%) | 85.7% (67.3%- 96%) |
| Group3 40-49 years | .757 (.586- .927) | 60% (26.2%- 87.8%) | 91.3% (72%- 98.9%) | 75% (34.9%- 96.8%) | 84% (63.9%- 95.5%) |
| Group4 50-59 years | .673 (.546- .801) | 52% (31.3%- 72.2%) | 82.6% (61.2%- 95%) | 76.5% (50.1%- 93.2%) | 61.3%( 42.2%- 78.2%) |
| Group4 >60 years | .725 (.551- .899) | 70% (34.8%- 93.3%) | 75% (53.3%- 90.2%) | 53.8%( 25.1%- 80.8%) | 85.7% (63.7%- 97%) |

| | | | | | |
|---|---|---|---|---|---|
| | | | | | |
| Cut off | 0.7 | | | | |
| Whole cohort | .701 (.629-.774) | 50% (36.3% - 63.7%) | 90.3% (82.9%-95.2%) | 73.7% (56.9%-86.6%) | 76.9% (68.3%-84%) |
| Group1 < 30 years | .625 .(38 - .87) | 25% (.631%-80.6%) | 100% (66.4%-100%) | 100% (2.5%-100%) | 75%( 42.8%-94.5%) |
| Group2 30-39 years | .643( .462 - .824) | 28.6% (3.67%-71%) | 100%( 85.8%-100%) | 100% (15.8%-100%) | 82.8% (64.2%-94.2%) |
| Group3 40-49 years | .707 (.533-.88) | 50% (18.7%-81.3%) | 91.3% (72%-98.9%) | 71.4% (29%-96.3%) | 80.8%( 60.6%-93.4%) |
| Group4 50-59 years | .673 (.546-.801) | 52% (31.3%-72.2%) | 82.6% (61.2%-95%) | 76.5% (50.1%-93.2%) | 61.3%( 42.2%-78.2%) |
| Group4 >60 years | .767 (.599-.935) | 70% (34.8%-93.3%) | 83.3%( 62.6%-95.3%) | 63.6% (30.8%-89.1%) | 87% (66.4%-97.2%) |
| | | | | | |
| Cut off | 0.8 | | | | |
| Whole cohort | .697( .626 - .769) | 48.2% (34.7%-62%) | 91.3% (84.1%-95.9%) | 75%( 57.8%-87.9%) | 76.4% (67.9%-83.6%) |
| Group1 < 30 years | .625 (.38-.87) | 25% .(631%-80.6%) | 100% (66.4%-100%) | 100% (2.5%-100%) | 75% (42.8%-94.5%) |
| Group2 30-39 years | .643( .462-.824) | 28.6% (3.67%-71%) | 100%( 85.8%-100%) | 100% (15.8%-100%) | 82.8%( 64.2% -94.2% |
| Group3 40-49 years | .678 (.513-.844) | 40% (12.2%-73.8%) | 95.7% (78.1%-99.9%) | 80% (28.4%-99.5%) | 78.6%( 59%-91.7%) |
| Group4 50-59 years | .673( .546-.801) | 52% (31.3%-72.2%) | 82.6% (61.2%-95%) | 76.5% (50.1%-93.2%) | 61.3%( 42.2%-78.2%) |
| Group4 >60 years | .767 (.599-.935) | 70% (34.8%-93.3%) | 83.3% (62.6%-95.3%) | 63.6% (30.8%-89.1%) | 87% (66.4%-97.2%) |
| | | | | | |
| Cut off | 0.9 | | | | |
| Whole cohort | .667 (.599-.736) | 39.3% (26.5%-53.2%) | 94.2% (87.8%-97.8%) | 78.6%( 59%-91.7%) | 74% (65.7%-81.3%) |
| Group1 < 30 years | .625 (.38-.87) | 25% (.631%-80.6%) | 100% (66.4%-100%) | 100%( 2.5%-100%) | 75% (42.8%-94.5%) |
| Group2 30-39 years | .643 (.462-.824) | 28.6% (3.67%-71%) | 100% (85.8%-100%) | 100% (15.8%-100%) | 82.8% (64.2%-94.2%) |
| Group3 40-49 years | .65 (.5 -.8) | 30% ( 6.67%- 65.2%) | 100% (85.2%-100%) | 100% (29.2%- 100%) | 76.7% ( 57.7%-90.1%) |
| Group4 50-59 years | .675 (.553-.797) | 48% (27.8%-68.7%) | 87% (66.4%-97.2%) | 80% (51.9%-95.7%) | 60.6% (42.1%-77.1%) |
| Group4 >60 years | .638 (.464-.811) | 40% (12.2%-73.8%) | 87.5% (67.6%-97.3%) | 57.1% (18.4%-90.1%) | 77.8% (57.7%-91.4%) |
| | | | | | |
| Cut off | 1.0 | | | | |
| Whole cohort | .663 (.596-.731) | 37.5%( 24.9%-51.5%) | 95.1% (89%-98.4%) | 80.8% (60.6%-93.4%) | 73.7% (65.3%-80.9%) |

| | | | | | |
|---|---|---|---|---|---|
| Group1 < 30 years | .625 (.38-.87) | 25% (.631%-80.6%) | 100% (66.4%-100%) | 100% (2.5%-100%) | 75%( 42.8% -94.5%) |
| Group2 30-39 years | .643 (.462-.824) | 28.6% (3.67%-71%) | 100%( 85.8%-100%) | 100% (15.8%-100%) | 82.8% (64.2%-94.2%) |
| Group3 40-49 years | .6 (.469 -.731) | 20% (2.52%-55.6%) | 100% (85.2%-100%) | 100% (15.8%-100%) | 74.2% (55.4%-88.1%) |
| Group4 50-59 years | .697 (.581-.813) | 48% (27.8%-68.7%) | 91.3%( 72%-98.9%) | 85.7% (57.2%-98.2%) | 61.8% (43.6%-77.8%) |
| Group4 >60 years | .638( .464-.811) | 40% (12.2%-73.8%) | 87.5% (67.6%-97.3%) | 57.1% (18.4%-90.1%) | 77.8% (57.7%-91.4%) |
| | | | | | |
| Cut off | 1.1 | | | | |
| Whole cohort | .654 (.588-.721) | 35.7% (23.4%-49.6%) | 95.1% (89%-98.4%)) | 80% (59.3%-93.2%) | 73.1% (64.8%-80.4%) |
| Group1 < 30 years | .625 (.38 -.87) | 25% (.631%-80.6%) | 100%( 66.4%-100% | 100% (2.5%-100%) | 75%( 42.8%-94.5%) |
| Group2 30-39 years | .643 (.462-.824) | 28.6% (3.67%-71%) | 100%( 85.8%-100%) | 100% (15.8% -100%) | 82.8% (64.2%-94.2%) |
| Group3 40-49 years | .6( .469-.731) | 20% (2.52%-55.6%) | 100% (85.2%-100%) | 100% (15.8% -100%) | 74.2% (55.4%-88.1%) |
| Group4 50-59 years | .697( .581-.813) | 48% (27.8%-68.7%) | 91.3% (72%-98.9%) | 85.7%( 57.2%-98.2%) | 61.8% (43.6%-77.8%) |
| Group4 >60 years | .588 (.423-.752) | 30% (6.67%-65.2%) | 87.5% (67.6%-97.3%) | 50% (11.8%-88.2%) | 75% (55.1%-89.3%) |
| | | | | | |
| Cut off | 1.2 | | | | |
| Whole cohort | .645 (.579-.711) | 33.9% (21.8% -47.8%) | 95.1% (89%-98.4%) | 79.2%( 57.8%-92.9%) | 72.6% (64.3%-79.9%) |
| Group1 < 30 years | .625 (.38 -.87) | 25% (.631%-80.6%) | 100%( 66.4%-100%) | 100% (2.5%-100%) | 75% (42.8%-94.5%) |
| Group2 30-39 years | .643 (.462-.824) | 28.6% (3.67% -71%) | 100%( 85.8%-100%) | 100% (15.8%-100%) | 82.8% (64.2%-94.2%) |
| Group3 40-49 years | .6 (.469-.731) | 20% (2.52%-55.6%) | 100% (85.2%-100%) | 100% (15.8%-100%) | 74.2% (55.4%-88.1%) |
| Group4 50-59 years | .677 (.561 -.792) | 44% (24.4%-65.1%) | 91.3% (72% -98.9%) | 84.6% (54.6%-98.1%) | 60% (42.1%-76.1%) |
| Group4 >60 years | .588 (.423-.75) | 30%( 6.67%-65.2%) | 87.5% (67.6%-97.3%) | 50% (11.8%-88.2%) | 75% (55.1%-89.3%) |
| | | | | | |
| Cut off | 1.3 | | | | |
| Whole cohort | .641 (.577- .706) | 32.1% (20.3%- 46%) | 96.1% (90.4%- 98.9%) | 81.8% (59.7%- 94.8%) | 72.3% (64%- 79.6%) |
| Group1 < 30 years | .625 (.38-.87) | 25% (.631% -80.6%) | 100%( 66.4%-100%) | 100% (2.5%-100%) | 75%( 42.8%-94.5%) |
| Group2 30-39 years | .643 (.462-.824) | 28.6% (3.67%-71%) | 100% (85.8%-100%) | 100%( 15.8%-100%) | 82.8% (64.2%-94.2%) |

| | | | | | |
|---|---|---|---|---|---|
| Group3 40-49 years | .6 (.469- .731) | 20% (2.52%- 55.6%) | 100%( 85.2%- 100%) | 100% (15.8%- 100%) | 74.2% (55.4%- 88.1%) |
| Group4 50-59 years | .657 (.542- .771) | 40% (21.1% -61.3%) | 91.3% (72%- 98.9%) | 83.3% (51.6%- 97.9%) | 58.3% (40.8%- 74.5%) |
| Group4 >60 years | .608 ( .448- .768) | 30% (6.67%- 65.2%) | 91.7% (73%- 99%) | 60% (14.7%- 94.7%) | 75.9% (56.5%- 89.7%) |
| | | | | | |
| Cut off | 1.4 | | | | |
| Whole cohort | .632 (.569- .696) | 30.4% (18.8%- 44.1%) | 96.1% (90.4%- 98.9%) | 81% (58.1%- 94.6%) | 71.7% (63.5%- 79.1%) |
| Group1 < 30 years | .625 (.38- .87) | 25% (.631%- 80.6%) | 100%( 66.4%- 100%) | 100% (2.5%- 100%) | 75% (42.8%- 94.5%) |
| Group2 30-39 years | .643 (.462 - .824) | 28.6% (3.67% - 71%) | 100% (85.8%- 100%) | 100% (15.8%- 100%) | 82.8%( 64.2%- 94.2%) |
| Group3 40-49 years | .6( .469- .731) | 20%( 2.52%- 55.6%) | 100% (85.2%- 100%) | 100% (15.8%- 100%) | 74.2% (55.4%- 88.1%) |
| Group4 50-59 years | .657 (.542- .771) | 40% (21.1%- 61.3%) | 91.3% (72% - 98.9%) | 83.3% (51.6%- 97.9%) | 58.3% (40.8%- 74.5%) |
| Group4 >60 years | .558 (.416- .701) | 20%( 2.52%- 55.6%) | 91.7% (73%- 99%) | 50% (6.76%- 93.2%) | 73.3% (54.1%- 87.7%) |
| | | | | | |
| Cut off | 0.15 | | | | |
| Whole cohort | .624 (.564- .684) | 26.8%( 15.8%- 40.3%) | 98.1%( 93.2%- 99.8%) | 88.2%( 63.6%- 98.5%) | 71.1% (62.9%- 78.4%) |
| Group1 < 30 years | | | | | |
| Group2 30-39 years | .643 (.462- .824) | 28.6%( 3.67%- 71%) | 100% (85.8%- 100%) | 100% (15.8%- 100%) | 82.8% (64.2%- 94.2%) |
| Group3 40-49 years | .6 (.469- .731) | 20% (2.52%- 55.6%) | 100% (85.2%- 100%) | 100% (15.8%- 100%) | 74.2% (55.4%- 88.1%) |
| Group4 50-59 years | .678 (.571- .785) | 40% (21.1%- 61.3%) | 95.7% (78.1%- 99.9%) | 90.9% (58.7%- 99.8%) | 59.5%( 42.1%- 75.2%) |
| Group4 >60 years | .529( .423- .635) | 10% (.253%- 44.5%) | 95.8% (78.9%- 99.9%) | 50% (1.26%- 98.7%) | 71.9% (53.3%- 86.3%) |
| Cut off | 0.16 | | | | |
| Whole cohort | .624 (.564- .684) | 26.8% (15.8%- 40.3%) | 98.1% (93.2%- 99.8%) | 88.2% (63.6%- 98.5%) | 71.1% (62.9%- 78.4%) |
| Group1 < 30 years | | | | | |
| Group2 30-39 years | .643 (.462- .824) | 28.6% (3.67%- 71%) | 100% (85.8%- 100%) | 100% (15.8%- 100%) | 82.8%( 64.2%0 94.2% |
| Group3 40-49 years | .6 (.469- .731) | 20% (2.52%- 55.6%) | 100%( 85.2%- 100%) | 100% (15.8%- 100%) | 74.2% (55.4%- 88.1%) |
| Group4 50-59 years | .678( .571- .785) | 40% (21.1%- 61.3%) | 95.7%( 78.1%- 99.9%) | 90.9% (58.7%- 99.8%) | 59.5% (42.1%- 75.2%) |

| | | | | | |
|---|---|---|---|---|---|
| Group4 >60 years | .529( .423-.635) | 10% (.253%-44.5%) | 95.8%( 78.9%-99.9%) | 50% (1.26%-98.7%) | 71.9% (53.3%-86.3%) |
| | | | | | |
| Cut off | 0.17 | | | | |
| Whole cohort | .615 (.557-.674) | 25% (14.4%-38.4%) | 98.1% (93.2%-99.8%) | 87.5%( 61.7%-98.4%) | 70.6% (62.4%-77.9%) |
| Group1 < 30 years | | | | | |
| Group2 30-39 years | .643( .462-.824) | 28.6% (3.67%-71%) | 100% (85.8%-100%) | 100% (15.8%-100%) | 82.8%( 64.2%-94.2%) |
| Group3 40-49 years | .6 (.469-.731) | 20% (2.52%-55.6%) | 100%( 85.2%-100%) | 100% (15.8%-100%) | 74.2% (55.4%-88.1%) |
| Group4 50-59 years | .678 (.571-.785) | 40% (21.1%-61.3%) | 95.7% (78.1%-99.9%) | 90.9% (58.7%-99.8%) | 59.5%( 42.1%-75.2%) |
| Group4 >60 years | .479 (.438-.52) | 0% (0%- 30.8%) | 95.8% (78.9%-99.9%) | 0%( 0% - 97.5%) | 69.7%( 51.3%-84.4%) |
| | | | | | |
| | | | | | |

**********************************CSI**********************************

**Diagnostic accuracy of combined sensory index (CSI), All cutoff values, (male participants).**

| Age group | ROC | Sensitivity | Specificity | PPV | NPV |
|---|---|---|---|---|---|
| Cut off | 1.0 | | | | |
| Whole cohort | .78( .721-.839) | 90.3% (80.1% - 96.4%) | 65.7%( 55.6%-74.8%) | 61.5%( 50.8%-71.6%) | 91.8%( 83%-96.9%) |
| Group1 < 30 years | .819 (.551-1) | 75% (19.4%-99.4%) | 88.9% (51.8%-99.7%) | 75% (19.4%-99.4%) | 88.9%( 51.8%-99.7%) |
| Group2 30-39 years | .909 (.827-.992) | 100%( 63.1%-100%) | 81.8% (59.7%-94.8%) | 66.7% (34.9%-90.1%.) | 100% (81.5%-100%) |
| Group3 40-49 years | .733 (.571-.896) | 80% (44.4%-97.5%) | 80%( 44.4%-97.5%) | 50% (24.7%-75.3%) | 88.9% (65.3%-98.6%) |
| Group4 50-59 years | .713( .588-.837) | 88% (68.8%-97.5%) | 54.5% (32.2%-75.6%) | 68.8% (50%-83.9%) | 80% (51.9%-95.7%) |
| Group4 >60 years | .76 (.66- .86) | 100% (78.2%-100%) | 52% (31.3%-72.2%) | 55.6% (35.3%-74.5%) | 100% (75.3%-100%) |
| Cut off | 1.1 | | | | |
| Whole cohort | .787 (.726-.847) | 88.7% (78.1%-95.3%) | 68.6%( 58.7%-77.5%) | 63.2% (52.2%-73.3%) | 90.9%( 82.2%-96.3%) |
| Group1 < 30 years | .819( .551-1) | 75% (19.4%-99.4%) | 88.9%( 51.8%-99.7%) | 75% (19.4%-99.4%) | 88.9%( 51.8%-99.7%) |
| Group2 30-39 years | .932( .858-1) | 100%( 63.1%-100%) | 86.4%( 65.1%-97.1%) | 72.7%( 39%-94%) | 100% (82.4%-100%) |
| Group3 40-49 years | .733 (.571-.896) | 80% (44.4%-97.5%) | 66.7% (44.7%-84.4%) | 50%( 24.7%-75.3%) | 88.9% (65.3%-98.6%) |
| Group4 50-59 years | .715( .587-.844) | 84% (63.9%-95.5%) | 59.1% (36.4%-79.3%) | 70% (50.6%-85.3%) | 76.5% (50.1%-93.2%) |
| Group4 >60 years | .78 (.681-.879) | 100% (78.2%-100%) | 56% (34.9%-75.6%) | 57.7%( 36.9%-76.6%) | 100% (76.8%-100%) |
| Cut off | 1.2 | | | | |
| Whole cohort | .806 (.747-.865) | 88.7%( 78.1%-95.3%) | 72.5% 62.8% 80.9% | 66.3%( 55.1%-76.3%) | 91.4%( 83%-96.5%) |
| Group1 < 30 years | .875 (.63 -1) | 75% (19.4%- 99.4%) | 100% (66.4%- 100%) | 100% (29.2%- 100%) | 90% (55.5%-99.7%) |
| Group2 30-39 years | .955 (.893-1) | 100% (63.1%-100%) | 90.9% (70.8%-98.9%) | 80% (44.4%-97.5%) | 100% (83.2%-100%) |
| Group3 40-49 years | .754 (.594-.914) | 80% (44.4%-97.5%) | 70.8%( 48.9%-87.4%) | 53.3%( 26.6%-78.7%) | 89.5%( 66.9%-98.7%) |
| Group4 50-59 years | .738 (.612-.865) | 84%( 63.9%-95.5%) | 63.6% (40.7%-82.8%) | 72.4% (52.8%-87.3%) | 77.8%( 52.4%-93.6%) |
| Group4 >60 years | .78( .681-.879) | 100% (78.2%-100%) | 56% (34.9%-75.6%) | 57.7%( 36.9% - 76.6%) | 100% (76.8%-100%) |
| Cut off | 1.3 | | | | |

| | | | | | |
|---|---|---|---|---|---|
| Whole cohort | .815 (.755-.875) | 85.5% (74.2%-93.1%) | 77.5%( 68.1%-85.1%) | 69.7%( 58.1%-79.8%) | 89.8%( 81.5%-95.2%) |
| Group1 < 30 years | .875 (.63 -1) | 75% (19.4% -99.4%) | 100%( 66.4%-100%) | 100% (29.2%-100%) | 90% (55.5%-99.7%) |
| Group2 30-39 years | .915 (.784 -1) | 87.5% (47.3%-99.7%) | 95.5% (77.2%-99.9%) | 87.5% (47.3%-99.7%) | 95.5% (77.2%-99.9%) |
| Group3 40-49 years | .796 (.641-.951) | 80% (44.4%-97.5%) | 79.2%( 57.8%-92.9%) | 61.5% (31.6%-86.1%) | 90.5%( 69.6%-98.8%) |
| Group4 50-59 years | .741 (.613-.869) | 80%( 59.3%-93.2%) | 68.2%( 45.1%-86.1%) | 74.1%( 53.7%-88.9%) | 75%( 50.9%-91.3%) |
| Group4 >60 years | .8 (.702 -.898) | 100%( 78.2%-100%) | 60% (38.7%-78.9%) | 60%( 38.7%-78.9%) | 100%( 78.2%-100%) |
| Cut off | 1.4 | | | | |
| Whole cohort | .792( .728-.857) | 79% (66.8% -88.3%) | 79.4% (70.3%-86.8%) | 70%( 57.9%-80.4%) | 86.2% (77.5%-92.4%) |
| Group1 < 30 years | .875 (.63 -1) | 75% (19.4%-99.4%) | 100% (66.4%-100%) | 100% (29.2%-100%) | 90%( 55.5%-99.7%) |
| Group2 30-39 years | .79 (.605-.975) | 62.5% (24.5%-91.5%) | 95.5% (77.2% -99.9%) | 83.3% (35.9%-99.6%) | 87.5% (67.6%-97.3%) |
| Group3 40-49 years | .817 (.665-.968) | 80%( 44.4%-97.5%) | 83.3% (62.6%-95.3%) | 66.7% (34.9%-90.1%) | 90.9%( 70.8%-98.9%) |
| Group4 50-59 years | .724( .593-.855) | 72% (50.6%-87.9%) | 72.7%( 49.8%-89.3%) | 75% (53.3%-90.2%) | 69.6%( 47.1%-86.8%) |
| Group4 >60 years | .8 .(702-.898) | 100%( 78.2%-100%) | 60% (38.7% -78.9%) | 60% (38.7%-78.9%) | 100% (78.2%-100%) |
| Cut off | 1.5 | | | | |
| Whole cohort | .789 (.724-.854) | 77.4%( 65%-87.1%) | 80.4%( 71.4%-87.6%) | 70.6%( 58.3%-81%) | 85.4%( 76.7%-91.8%) |
| Group1 < 30 years | .875 (.63 -1) | 75%( 19.4%-99.4%) | 100%( 66.4%-100%) | 100%( 29.2%-100%) | 90%( 55.5%-99.7%) |
| Group2 30-39 years | .813 ( .633- .992) | 62.5% (24.5%- 91.5%) | 100% (84.6%- 100%) | 100% (47.8%- 100%) | 88% (68.8%-97.5%) |
| Group3 40-49 years | .817 (.665-.968) | 80% (44.4%-97.5%) | 83.3% (62.6%-95.3%) | 66.7%( 34.9%-90.1%) | 90.9%( 70.8%-98.9%) |
| Group4 50-59 years | .704 (.57 -.837) | 68% (46.5%-) 85.1% | 72.7% (49.8%-89.3%) | 73.9%( 51.6%-89.8%) | 66.7% (44.7%-84.4%) |
| Group4 >60 years | .8 (.702-.898) | 100% (78.2%-100%) | 60%( 38.7%-78.9%) | 60% (38.7%-78.9%) | 100%( 78.2%-100%) |
| Cut off | 1.6 | | | | |
| Whole cohort | .765 (.697-.833) | 72.6%( 59.8% -83.1%) | 80.4% (71.4%-87.6%) | 69.2%( 56.6%-80.1%) | 82.8% (73.9%-89.7%) |
| Group1 < 30 years | .75 (.467- 1) | 50%( 6.76%-93.2%) | 100% (66.4%-100%) | 100% (15.8%-100%) | 81.8% (48.2%-97.7%) |
| Group2 30-39 years | .813 (.633-.992( | 62.5% (24.5%-91.5%) | 100%( 84.6% -100%) | 100% (47.8%-100%) | 88% (68.8%-97.5%) |

| | | | | | |
|---|---|---|---|---|---|
| Group3 40-49 years | .817( .665-.968) | 80%( 44.4%-97.5%) | 83.3% (62.6%-95.3%) | 66.7%( 34.9%-90.1%) | 90.9%( 70.8%-98.9%) |
| Group4 50-59 years | .684 (.548-.819) | 64% (42.5% -82%) | 72.7%( 49.8%-89.3%) | 72.7%( 49.8%-89.3%) | 64%( 42.5%-82%) |
| Group4 >60 years | .767 (.649-.884) | 93.3%( 68.1%-99.8%) | 60% (38.7%-78.9%) | 58.3% (36.6%-77.9%) | 93.8%( 69.8%-99.8%) |
| Cut off | 1.7 | | | | |
| Whole cohort | .767 .(699 -.835) | 71% (58.1%-81.8%) | 82.4% 7(3.6%-89.2%) | 71% (58.1%-81.8%) | 82.4% (73.6%-89.2%) |
| Group1 < 30 years | .625 (.38 -.87) | 25% (.631%-80.6%) | 100% (66.4%-100%) | 100%( 2.5%-100%) | 75%( 42.8%-94.5%) |
| Group2 30-39 years | .813( .633-.992) | 62.5% (24.5%-91.5%) | 100% (84.6%-100%) | 100% (47.8%-100%) | 88%( 68.8%-97.5%) |
| Group3 40-49 years | .817 (.665-.968) | 80% (44.4%-97.5%) | 83.3%( 62.6%-95.3%) | 66.7% (34.9%-90.1%) | 90.9% (70.8%-98.9%) |
| Group4 50-59 years | .684( .548-.819) | 64% (42.5% -82%) | 72.7% (49.8%-89.3%) | 72.7%( 49.8%-89.3%) | 64% (42.5%-82%) |
| Group4 >60 years | .807( .693-.921) | 93.3%( 68.1%-99.8%) | 68%( 46.5%-85.1%) | 63.6% (40.7%-82.8%) | 94.4%( 72.7%-99.9%) |
| Cut off | 2.0 | | | | |
| Whole cohort | .772 (.705-.839) | 66.1%( 53%-77.7%) | 88.2%( 80.4%-93.8%) | 77.4%( 63.8%-87.7%) | 81.1%( 72.5%-87.9%) |
| Group1 < 30 years | .625 (.38-.87) | 25% (.631%-80.6%) | 100%( 66.4%-100%) | 100%( 2.5%-100%) | 75%( 42.8%-94.5%) |
| Group2 30-39 years | .688 (.508 -.867) | 37.5% (8.52%-75.5%) | 100%( 84.6%-100%) | 100% (29.2%-100%) | 81.5% (61.9%-93.7%) |
| Group3 40-49 years | .817 (.665-.968) | 80% (44.4%-97.5%) | 83.3% (62.6%-95.3%) | 66.7%( 34.9%-90.1%) | 90.9%( 70.8%-98.9%) |
| Group4 50-59 years | .732( .609-.854) | 60% (38.7%-78.9%) | 86.4%( 65.1%-97.1%) | 83.3% (58.6%-96.4%) | 65.5%( 45.7%-82.1%) |
| Group4 >60 years | .867( .763-.97) | 93.3% (68.1%-99.8%) | 80%( 59.3%-93.2%) | 73.7%( 48.8%-90.9%) | 95.2%( 76.2%-99.9%) |
| Cut off | 2.5 | | | | |
| Whole cohort | .74 .(673-.807) | 54.8% (41.7%-67.5%) | 93.1% (86.4%-97.2%) | 82.9% (67.9%-92.8%) | 77.2%( 68.8%-84.3%) |
| Group1 < 30 years | .625 (.38-.87) | 25% (.631%-80.6%) | 100%( 66.4%-100%) | 100% (2.5%-100%) | 75%( 42.8%-94.5%) |
| Group2 30-39 years | .688 (.508-.867) | 37.5% (8.52%-75.5%) | 100%( 84.6%-100%) | 100% (29.2%-100%) | 81.5% (61.9%-93.7%) |
| Group3 40-49 years | .658 (.489-.828) | 40% (12.2%-73.8%) | 91.7% (73%-99%) | 66.7% (22.3%-95.7%) | 78.6% (59%-91.7%) |
| Group4 50-59 years | .735( .618-.851) | 56% (34.9%-75.6%) | 90.9% (70.8% -98.9%) | 87.5% (61.7%-98.4%) | 64.5% (45.4%-80.8%) |
| Group4 >60 years | .84 (.717-.963) | 80%( 51.9%-95.7%) | 88%( 68.8%-97.5%) | 80% (51.9%-95.7%) | 88%( 68.8%-97.5%) |
| Cut off | 3.5 | | | | |

| | | | | | |
|---|---|---|---|---|---|
| Whole cohort | .695 (.631-.759) | 41.9%( 29.5%-55.2%) | 97.1% (91.6%-99.4%) | 89.7%( 72.6%-97.8%) | 73.3% (65%-80.6%) |
| Group1 < 30 years | | | | | |
| Group2 30-39 years | .688 (.508-.867) | 37.5%( 8.52%-75.5%) | 100% (84.6%-100%) | 100%( 29.2%-100%) | 81.5% (61.9%-93.7%) |
| Group3 40-49 years | .65 (.5 -.8) | 30% ( 6.67%- 65.2%) | 100% (85.8%- 100%) | 100% (29.2%- 100%) | 77.4% (58.9%-90.4%) |
| Group4 50-59 years | .697 (.588-.806) | 44% (24.4%-65.1%) | 95.5%( 77.2%-99.9%) | 91.7%( 61.5%-99.8%) | 60% (42.1%-76.1%) |
| Group4 >60 years | .76 (.621-.899) | 60%( 32.3%-83.7%) | 92% (74% -99%) | 81.8% (48.2%-97.7%) | 79.3% (60.3%-92%) |
